# Facile Synthesis of Hemin Derivatives with Modulated Aggregation Behaviour and Enhanced Nitric‐Oxide Scavenging Properties as New Therapeutics for Breast Cancer

**DOI:** 10.1002/smsc.202400237

**Published:** 2024-07-16

**Authors:** Amir M. Alsharabasy, Durgadas Cherukaraveedu, Jonas Warneke, Ziyan Warneke, José Ramón Galán‐Mascarós, Sharon A. Glynn, Pau Farràs, Abhay Pandit

**Affiliations:** ^1^ CÚRAM SFI Research Centre for Medical Devices University of Galway H91 W2TY Galway Ireland; ^2^ Wilhelm‐Ostwald‐Institut für Physikalische und Theoretische Chemie Universität Leipzig 04103 Leipzig Germany; ^3^ Leibniz Institute of Surface Engineering (IOM) Permoserstraße 15 04318 Leipzig Germany; ^4^ Institute of Chemical Research of Catalonia (ICIQ) The Barcelona Institute of Science and Technology (BIST) Av. Països Catalans 16 ES‐43007 Tarragona Spain; ^5^ Catalan Institution for Research and Advanced Studies (ICREA) Passeig Lluis Companys 16 ES‐08007 Barcelona Spain; ^6^ Discipline of Pathology Lambe Institute for Translational Research School of Medicine University of Galway H91 YR71 Galway Ireland; ^7^ School of Biological and Chemical Sciences Ryan Institute University of Galway H91 TK33 Galway Ireland

**Keywords:** aggregation, hemin, metastasis, nitric oxide, nityrosylation, triple‐negative breast cancer, tyrosine

## Abstract

Nitric oxide (^•^NO) plays various pathophysiological roles in breast cancer, significantly influencing the migration of tumour cells through concentration gradients. Therefore, modulating ^•^NO levels via selective scavenging presents a promising approach to treating aggressive ^•^NO‐dependent cancers, such as triple‐negative breast cancer (TNBC). Hemin emerges as a potential scavenger of ^•^NO; however, its metalloporphyrin molecules tend to aggregate in physiological solutions, which limits its biomedical applications. To address this, a modification strategy is employed to minimize aggregation and protect against physiological oxidative degradation while preserving ^•^NO‐scavenging properties. This is achieved through a simple chemical transformation that involves hemin conjugation to aromatic residues, tyrosine, and tyramine via carbodiimide reactions. These derivatives exhibit altered electronic properties and oxidation potential compared to hemin, alongside reduced aggregation tendencies and retained ^•^NO‐binding affinity in aqueous solutions. Furthermore, depending on the type of hemin derivative, there is an associated inhibition of TNBC cell migration. These model hemin compounds demonstrate varying ^•^NO‐binding affinities and resistance levels to oxidative degradation and aggregation, offering insights into the design of ^•^NO‐scavenging molecules with enhanced properties for cancer treatment.

## Graphical abstract

Scheme depicting the influence of nitric oxide (^*•*^NO) with/without hemin or hemin derivatives on cell migration (measured by scratch assay) and the impact of conjugation to aromatic residues on aggregation, oxidative degradation, and ^*•*^NO‐mediated cellular uptake. Atoms are color‐coded: carbon (gray), oxygen (red), nitrogen (blue), and iron (blue violet).

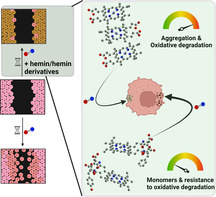

## 1 Introduction

Hemin is a coordinate complex of iron (Fe(III)) and protoporphyrin IX (PPIX), and is considered the oxidized form of heme, which serves as the prosthetic groups in various vital proteins and enzymatic systems.^[^ ^1^ ^]^ These include hemoglobin, myoglobin, and various peroxidases and cytochromes.^[^ ^2^, ^3^ ^]^ This functionality relates to the tetrapyrrole scaffold coordinated to central iron, where the former works as a redox‐active ligand with an electron‐rich *π*‐system.^[^ ^4^ ^]^ This coordination facilitates the oxidation/reduction reactions of the central metal, responsible for its multiple catalytic functions.^[^ ^4^ ^]^ However, considering the biomedical applications of hemin, its molecules suffer from the dimerization and further aggregation in aqueous solutions. These reactions are promoted by the formed oxo‐bridges between the central metal atoms, *π*–*π* stacking of the porphyrin rings, polarizability of the peripheral substituents, electrostatic and hydrophobic interactions.^[^ ^5^ ^]^

Nitric oxide (^•^NO) is one of the significant pro‐oxidant molecules affecting the tumour biology, where it can act as both pro‐ and anti‐tumorigenic transmitter depending on its dose and duration of exposure. For instance, ^•^NO plays vital roles in modulating the cellular transformation, invasion, and metastasis.^[^ ^6^ ^]^ These roles are mediated by its influence on certain signaling cascades in the cancer cells, or on specific structural and matricellular proteins.^[^ ^7^, ^8^ ^]^ Hence, the inhibition of ^•^NO synthesis has been proposed as a potential strategy for the treatment of breast cancer.^[^ ^9^, ^10^, ^11^ ^]^ However, another approach for decreasing the levels of intracellular ^•^NO depends on the scavenging of its excessive concentrations, without inhibition of the activity of ^•^NO synthases. We examined before how hemin can scavenge ^•^NO, implicate nitration of cellular proteins,^[^ ^12^ ^]^ and inhibit the ^•^NO‐promoted cancer cell migration.^[^ ^13^ ^]^ Moreover, the inhibitory effects of hemin for the ^•^NO‐induced fragmentation of hyaluronic acid were reported.^[^ ^14^ ^]^ However, to develop a sustainable ^•^NO‐scavenging mechanism in physiological solutions and address the challenges associated with hemin aggregation, structural modification is necessary.

**Scheme 1:**
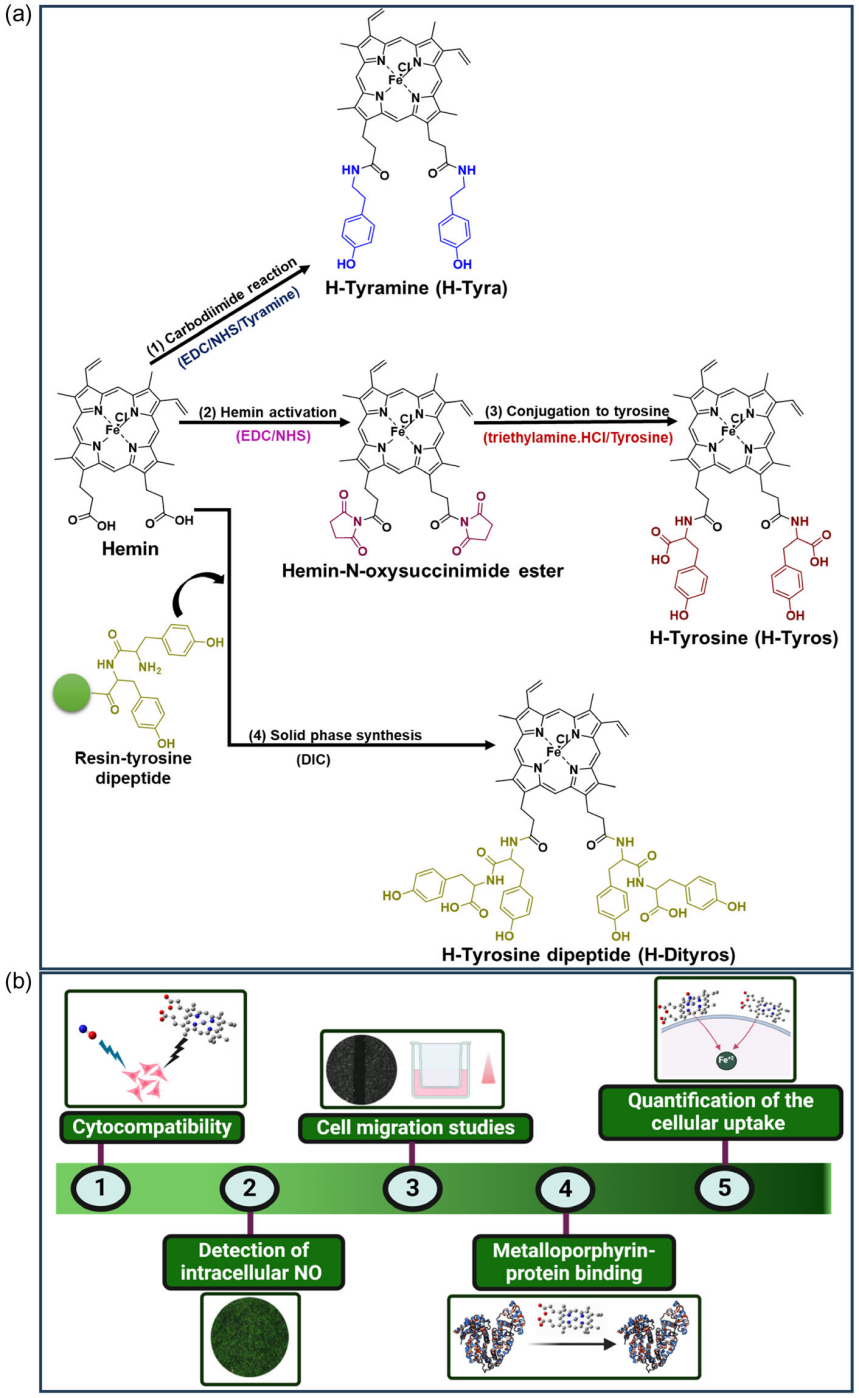
a) General procedures for hemin modification, including conjugation to tyramine by carbodiimide reaction (1), activation and formation of hemin‐N‐oxysuccinimide ester (2), followed by conjugation to tyrosine (3), and conjugation to tyrosine dipeptide through solid‐phase peptide synthesis (4). b) The in vitro assessment of different compounds involving testing the cytocompatibility, detecting intracellular ^*•*^NO, cell migration studies, evaluating metalloporphyrin‐protein binding and quantifying cellular uptake.

Previously, we described a strategy for hemin modification via cross‐metathesis reaction and conjugation to styrene.^[^ ^15^ ^]^ In this study, we further modified hemin by conjugating it to tyrosine and tyramine through carbodiimide reaction and to tyrosine dipeptide via solid‐phase peptide synthesis. The resulting derivatives’ structural properties were evaluated, alongside characterization of their magnetic properties in monomer and aggregate forms. Subsequently, the affinity of each compound toward ^*•*^NO was assessed through electrochemical detection, and the complexation reactions were examined using density functional theory (DFT) at the quantum mechanics level. Additionally, the binding affinity between each hemin derivative and bovine serum albumin (BSA) as a model protein was investigated. Following this, the cytocompatibility of these compounds against triple‐negative breast cancer (TNBC) cell lines was evaluated, and their cellular uptake was quantified through measurement of cellular iron content. Furthermore, intracellular ^*•*^NO levels were monitored after treating normal and inducible nitric oxide synthase (iNOS)‐transduced cells with different compounds in the presence or absence of extracellular ^*•*^NO. Finally, the downstream effects of ^*•*^NO scavenging were examined by measuring cell migration in response to ^*•*^NO in the presence or absence of the different compounds using a scratch assay. A scheme depicting the general synthesis procedures for various hemin derivatives and the in vitro testing methods employed is presented in **Scheme** **1**.

## 2 Results and Discussion

Various metal‐based compounds have been reported in the literature as ^*•*^NO‐scavenging moieties, including complexes based on ruthenium, titanium, cobalt, and copper.^[^ ^16^, ^17^, ^18^, ^19^ ^]^ Additionally, non‐metal‐based ^*•*^NO‐scavenging structures have been developed, showing potential in ameliorating ^*•*^NO‐induced inflammation.^[^ ^20^, ^21^ ^]^ In this study, hemin was employed for ^*•*^NO‐scavenging, leveraging the naturally reported ^*•*^NO‐binding affinity of heme within myoglobin and hemoglobin protein moieties.^[^ ^22^, ^23^, ^24^ ^]^ Furthermore, NO‐ferroheme species have been proposed as signaling model compounds in vasculature.^[^ ^25^ ^]^ Additionally, nitrosylated hemin‐loaded nanocubes were utilized for controlled delivery of NO.^[^ ^26^ ^]^

### 2.1 Hemin Modification

**Figure 1:**
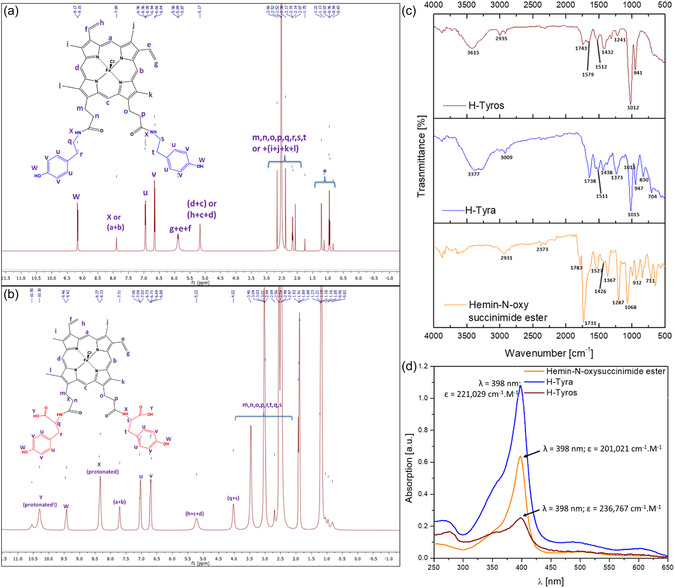
a,b) ^1^H NMR (500 MHz, DMSO‐d6, 25 °C) spectra of H‐Tyra and H‐Tyros, respectively. *solvent impurities. c) ATR‐FTIR spectra for hemin‐N‐oxysuccinimide ester, H‐Tyra and H‐Tyros. d) UV‐Vis spectra for all compounds in DMSO.

Different hemin derivatives were synthesized and purified by column chromatography. The nuclear magnetic resonance (NMR) spectrum of hemin relates to a six‐coordinate high‐spin system. This was consistent with the results obtained before,^[^ ^27^ ^]^ and explained in our previous work.^[^ ^15^ ^]^ However, all the ^1^H‐NMR spectra of hemin derivatives were observed within a narrow detection range (**Figure** **1**a,b). These differences relate to either one or more of the following factors: 1) effects of the substituent groups and whether they work as electron‐withdrawing or donating groups; 2) the paramagnetic properties of the central Fe atoms and the strength of anti‐ferromagnetic exchange coupling between them; 3) basicity of N‐atoms in the pyrrole rings; and/or 4) possible formation of oxygen‐bridges between the monomeric units.

Hemin‐tyramine (H‐Tyra) and hemin‐tyrosine (H‐Tyros) were synthesized via a conventional EDC/NHS‐based carbodiimide reaction, by a method published by Okorochenkov et al.^[^ ^28^ ^]^ with some modifications. EDC was used to activate the –COOH groups of hemin in DMSO only, and the conjugation with tyramine and tyrosine was performed in one and two steps, respectively. For the precipitation of the final products, a mixture of diethyl ether and saturated NaCl solution was added. Here, the ether displaces water from DMSO, causing an increase in the water‐to‐DMSO ratio as an example of the Gibbs triangle of three solvents. This facilitated the precipitation of the final compound in the presence of NaCl, which was then washed and purified. The yield of H‐Tyra was 80%. The structure of the separated H‐Tyra was confirmed by NMR spectroscopy (Figure 1a). A comparison between the UV‐Vis and FTIR spectra of activated hemin, H‐Tyra, H‐Tyros is in Figure 1c,d, while the FTIR spectrum of H‐Dityros is in Figure S1a, Supporting Information. The FTIR spectra of tyramine and tyrosine are in Figure S2a,b, Supporting Information.

Since tyrosine's solubility in different solvents is limited and because there is a need to perform the carbodiimide reaction in the lowest amounts of DMSO, various solvent mixtures were tested for dissolving the amino acid. The final solvent mixture consisted of 90% DMSO, 8.33% HCl (37%) and 1.67% double distilled water. Hence, a maximum concentration of 100 mg mL^−1^ of tyrosine was achieved following the dissolution at room temperature for a few minutes. This reached 400 mg mL^−1^ with heating in a water bath at 90 °C. The pH of the final solution was 1.44, and minute quantities were used for the conjugation reaction. In contrast to H‐Tyra, the yield of H‐Tyros was 70% due to the two‐step reaction. NMR spectroscopy confirmed the structure of H‐Tyros derivative, with the presence of key signals at 9.44 and 10.3 ppm corresponding to the –OH hydrogen atoms beside hydrogens of the main porphyrin ring (Figure 1b). In contrast to H‐Tyros, the bands in the ^1^H NMR spectrum of H‐Dityros could not be fully distinguished (Figure S1b, Supporting Information).

### 2.2 Aggregation and Gas Phase Reactivity

**Figure 2:**
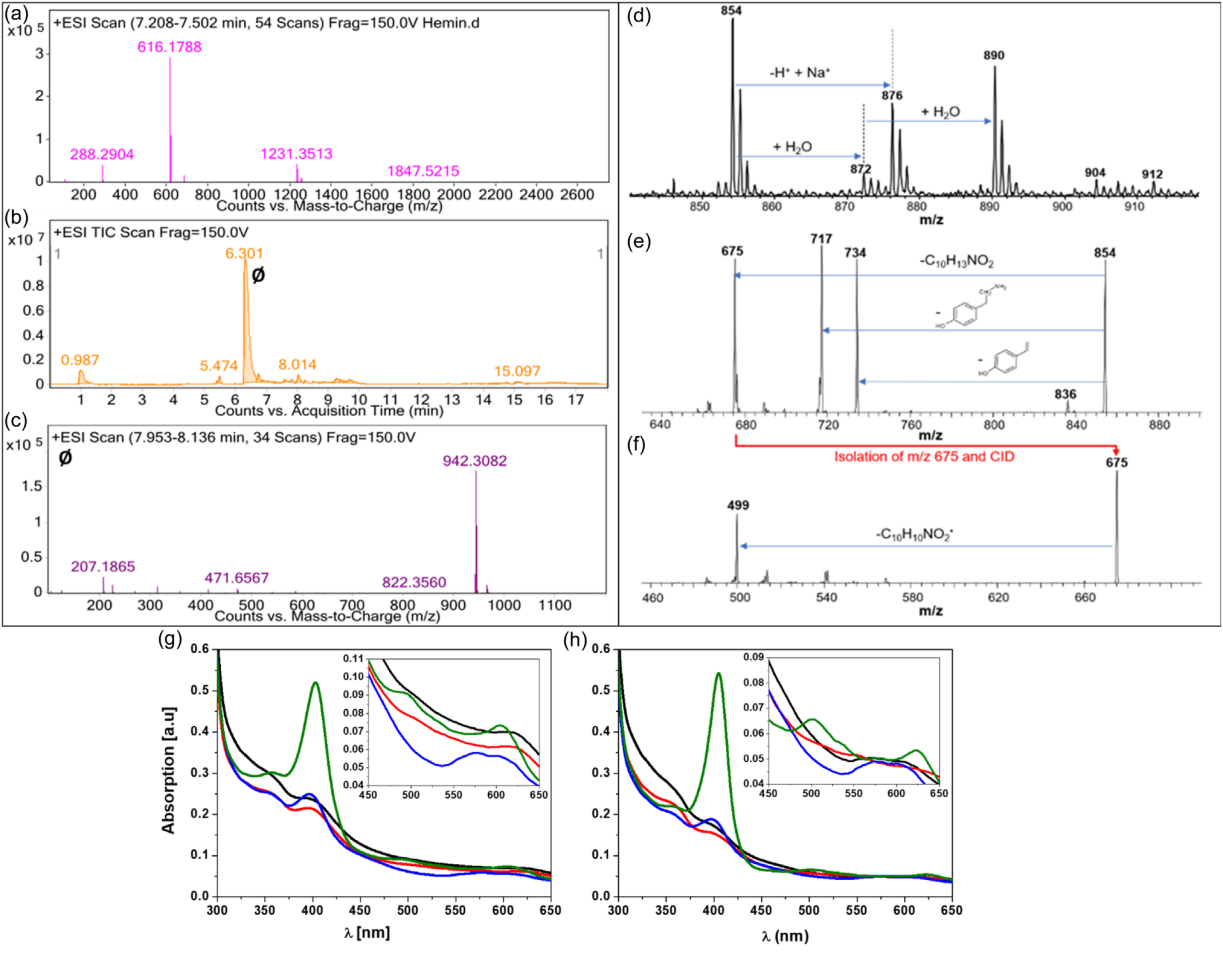
a) The measured mass spectrum of hemin from LC‐MS. b) Reverse‐phase HPLC separation of H‐Tyros (*Ø*) and c) the corresponding mass spectra from LC‐MS. d) ESI mass spectra of H‐Tyra [C_50_H_50_FeN_6_O_4_]^+^ (*m*/*z* 854). e) Isolation and fragmentation of the molecular ion of tyramine. f) isolation and further fragmentation of the fragment ion at *m*/*z* 675. g,h) UV‐Vis spectrum of H‐Tyra (g) and H‐Tyros (h) in 100% DMSO (green line), FBS‐free RPMI (black line), phosphate buffer (red line) and pyridine/NaOH solutions (blue line). Inset: the spectra within the range of 450–650 nm. Results are presented as mean values, *n* = 3.

Using LC‐MS, the hemin ion was detected at m/z 616, corresponding to [C_34_H_32_FeN_4_O_4_]^+^), with other three relatively weak peaks at: 1) *m*/*z* 288.29; 2) *m*/*z* 1232.35: assigned to the hemin dimer ion [2M‐H]^+^ via coordination of –COO^−^ group in one molecule with the central iron in another hemin molecule^[^ ^29^ ^]^; and 3) *m*/*z* 1847.52: assigned to the hemin trimer ion [3M‐2H]^+^ (**Figure** **2**a). A detailed explanation of the aggregation mechanism of hemin was reported before.^[^ ^15^ ^]^ The MW of the H‐Tyros derivative was confirmed with the detection of an abundant signal at m/z 942.3 for [C_52_H_50_FeN_6_O_8_]^+^ (Figure 2b,c). However, the MW of neither H‐Tyra nor H‐Dityros could be verified using LC‐MS, but only using direct injection Electrospray‐Ionization (ESI‐MS).

ESI is a valuable method for transferring molecular ions, like hemin and its derivatives, into the gas phase of mass spectrometers. Here, an ion with a selected m/z value can be isolated in an ion trap and then fragmented using collision‐induced dissociation (CID) to study the fragmentation of this ion. This provides information on the intrinsic properties of the ions without the influence of solvents or counterions. Hemin fragments by successive elimination of two CH_2_COOH^•^ units resulting in an ion at *m*/*z* 498. The molecular ion of H‐Tyra was observed at *m*/*z* 854. Abundant ions at larger *m*/*z* were assigned to aggregates with water, and the exchange of protic H^+^ by Na^+^ (a common phenomenon in ESI‐MS), see Figure 2d. CID of the H‐Tyra molecular ion resulted in the decomposition of the Tyramine units. The cleavage of the C—C bond between α‐ and b‐carbon next to the carbonyl group was also observed in this derivative similar to the unmodified hemin ion. However, first, an even electron neutral loss of C_10_H_13_NO_2_ was observed (Figure 2e), followed by a radical loss of C_10_H_10_NO_2_^•^ (Figure 2f). Therefore, the resulting fragment at *m*/*z* 499 formed by the loss of both side chains contains an additional H‐atom compared to the fragment formed from CID of the free hemin. These fragments are therefore of different spin multiplicity. These results were confirmed using a different ion trap instrument, comparing the fragmentation of hemin and H‐Tyra.

In contrast to the LC‐MS analysis of H‐Tyros, the ion could not be detected using ESI‐MS, likely due to the instability of the product (Figure S3, Supporting Information). This hindered CID studies and indicates that the decomposition of H‐Tyros occurred at a rate much faster than that of H‐Tyra. Also, the MW of H‐Dityros could not be verified using ESI‐MS.

The aggregation of different compounds was then tested using UV‐Vis spectroscopy in the following solutions: 1) DMSO, as the main organic solvent of all compounds; 2) phosphate buffer and DMEM culture medium, as the aqueous solutions used in the ^•^NO‐measurement experiments and cell culture; and 3) pyridine/NaOH solution, employed for inducing the formation of μ‐oxo bridges between the central iron atoms.^[^ ^30^, ^31^ ^]^ Each compound showed a particular spectrum in each solvent, with a high similarity between those in the buffer and those in the culture medium. In DMSO, hemin and other various metalloporphyrin molecules exist mainly in monomeric form, characterized by a sharp Söret band at around 400 nm and weak bands within the range of 500–600 nm (data not shown). However, these molecules tend to aggregate in aqueous solutions, with formation of *π*–*π* stacks between the monomeric units in addition to μ‐oxo bridges between the central metals.^[^ ^32^ ^]^ In case of hemin, the formed dimers and trimers have different electronic distribution within the whole porphyrin structure, compared to that of the monomeric molecules. This causes a blue shifting of the Söret band, with a decrease in the intensity, in combination with broader charge transfer bands, as we reported before.^[^ ^15^ ^]^ In phosphate buffer, for example, this blue shifting was prominent in the case of hemin, followed by H‐Tyra, with a slight shift in H‐Tyros (Figure 2g,h; **Table** **1**), but with no differences in the case of H‐Dityros (Figure S4a, Supporting Information). In pyridine/NaOH solution, a drop in intensity of the Söret band was observed owing to the enhanced deprotonation of the axial water ligand on hematin. Moreover, the blue shifting was like that in phosphate buffer in the case of all compounds. In addition, the spectrum within the charge transfer region, as a signature for the μ‐oxo dimers, showed differences in the detected peaks in case of all compounds except for H‐Dityros (Table 1).

**Table 1:** Wavelengths of the Soret band and the main bands in the charge transfer region in nm.

	100% DMSO		Phosphate buffer		Pyridine/NaOH	
	Soret band	Charge transfer region	Soret band	Charge transfer region	Soret band	Charge transfer region
Hemin	406	572, 602	394	608	396	574, 602
H‐Tyra	402	490, 604	395	612	396	574, 602
H‐Tyros	404	502, 622	400	–	398	574, 602
H‐Dityros	398	602	398	–	396	594

To further confirm the formation of dimers, the NMR spectra of hemin, H‐Tyros and H‐Dityros were measured. In the case of hemin in NaOD/pyridine‐d_5_/D_2_O solvent, the ^1^H‐NMR spectrum was detected within the normal spectral range with less line broadening (Figure S5, Supporting Information) than that in DMSO‐d_6_ only (Figure 1b). However, in the case of H‐Tyros, the peaks of phenyl hydrogens were additionally detected, with a better resolution of the peaks than in hemin (Figure S6, Supporting Information). These observations in the ^1^H‐NMR spectra corresponding to the μ‐oxo dimer were suggested to be caused by an anti‐ferromagnetic coupling between the two Fe(III) centres across the O‐bridge,^[^ ^30^, ^31^ ^]^ which were absent in the spectra in DMSO‐d_6_. Furthermore, the bands of H‐Dityros could not be fully distinguished (Figure S7, Supporting Information). In the case of hemin^[^ ^32^ ^]^ and other metalloporphyrins,^[^ ^33^ ^]^ several studies utilizing magnetic susceptibility measurements, UV‐Vis spectroscopy, and quantum mechanics calculations have demonstrated that dimerization causes antiferromagnetic exchange coupling. This results in a ^1^H‐NMR spectrum more typical of Fe(II) complexes in aqueous solutions. These results indicate that these compounds in different solvents showed comparable speciation patterns based on their chemical structures and solution composition. However, the effects of compound concentration, temperature and pH of solution were not covered in the current study as they are outside its focus. The effects of these patterns on the ^•^NO‐scavenging by each compound will be covered in the next section.

### 2.3 Quantum Mechanics Calculations

The main minimum state following the complexation of central Fe(III) with ^•^NO is characterized by a linear Fe—NO bond, formed by transfer of the unpaired electron from ^•^N to Fe(III).^[^ ^34^, ^35^ ^]^ The electronic configuration in that case was described as [Fe(II)–NO]^+^ (S = 0), characterized by the formation of low spin Fe(II).^[^ ^36^ ^]^ However, this linearity is distorted under certain conditions resulting in lower binding affinity between the central iron and ^•^NO. This is due to the anti‐ferromagnetic coupling between the unpaired electron of ^•^NO and low‐spin Fe(III), preventing its reduction, with a possible release of ^•^NO.^[^ ^37^, ^38^, ^39^ ^]^ Moreover, owing to the probable excitation of the [Fe(II)–NO]^+^ state, the Fe‐porphyrin‐NO complexation in that case can convert into the [Fe(III)–NO] state, with a relatively higher dissociation along the Fe–NO coordinate.^[^ ^35^, ^40^ ^]^ However, studying of electronic configuration under the latter case was out of the scope of this work.

Previously, we reported the structural parameters from the optimized geometries of hemin and hemin‐styrene conjugate before and after complexation with ^•^NO.^[^ ^15^, ^41^ ^]^ The calculated parameters in case of H‐Tyra and H‐Tyros are in **Table** **2**. Nitrogen atom of ^•^NO was starred to be distinguished from the N atoms of the pyrrole rings. The XYZ files in the case of H‐Tyra and H‐Tyros are summarized in Table S1 and S3, Supporting Information, respectively, while Table S2 and S4, Supporting Information, summarize the results in case of geometries with ^•^NO complexation, respectively. For the optimized geometries of all compounds, the Fen*—O* units were linear with similar Fe—N* and N*—O* bond lengths as well as ∠(N—Fe—N) and the energy of binding with ^•^NO (Table 2). In addition, there were no significant differences between these values and those of hemin (data not shown). Furthermore, the ∠(Fe–N*=O*) relates to a nearly linear Fe–N*=O* conformation in all structures. This was accompanied with out of the porphyrin‐plane movement of the Fe atom toward the ^•^NO, with an increase in bond length between Fe and each N‐atom of the pyrrole rings in all ^•^NO‐complexes. Explanation of the underlying molecular changes was reported before.^[^ ^42^, ^43^ ^]^ In brief, the partially occupied *π*_g_* orbital in ^•^NO has lower energy placing it closer to *d*‐orbitals of Fe,^[^ ^44^, ^45^ ^]^ allowing the flow of electronic charge from ^•^NO to Fe‐PPIX,^[^ ^43^ ^]^ with stabilization of the bonding between the orbitals in both atoms.^[^ ^42^ ^]^ Hence, the configuration in case of Fe(III)‐NO at *M* = 4 spin multiplicity was expressed as [Fe(II)–NO]^+^. However, in the case of [Fe(III))–NO] with a bent Fe)–N)–O geometry, one orbital of ^•^NO will have *π*_y_* character, with involving of the other orbital in *σ*‐bonding with the *d*_*z*2_ orbital of Fe.^[^ ^42^ ^]^ This latter case was out of the scope of the current work.

**Table 2:** Comparison between some of the structural parameters in the optimized geometries of H‐Tyra and H‐Tyros complexes before and after complexation with ^•^NO and the accompanied computed binding energy in terms of thermal energy (E) based on the [Fe(II)–NO]^+^ (*S* = 0) electronic state calculated at the level of DFT/B3LYP/Gen ECP *in vacuo*. The basis sets: LANL2DZ for Fe atom; 6−31 + G(d,p) for H atom, 6‐311 + G(d,p) for C, O, N, and Cl atoms. The energies in parenthesis are for the reduced Fe(II)‐porphyrin form corresponding to each main compound.

	H‐Tyra		H‐Tyros	
	Fe(III)	[Fe(II)‐NO] ^+^	Fe(III)	[Fe(II)‐NO] ^+^
R (Fe–Cl) [Å]	2.320		2.290	
R (Fe–N*) [Å]	–	1.602	–	1.602
R (O*–N*) [Å]	–	1.134	–	1.134
R (Fe–N) [Å]	2.008, 2.021, 2.021, 2.017	2.004, 1.993, 1.995, 1.993	2.023, 2.023, 2.019, 2.018	1.988, 1.991, 1.992, 2.002
∠ (N–Fe–Cl) [°]	97.31, 99.00, 97.27, 99.67	–	97.83, 98.30, 98.76, 98.17	–
∠ (Fe–N*–O*) [°]	–	179.77	–	179.35
∠ (N–Fe–N*) [°]	–	97.24, 101.00, 97.42, 101.10	–	98.06, 100.5, 97.97, 100.49
∠ (N–Fe–N) [°]	165.42, 161.34	165.35, 157.88	163.41, 163.53	163.97, 159.00
Dipole moment [Debye]	4.89	16.06	13.94	19.56
Polarizability [a.u]	817.51	798.18	857.21	509.29
*E* [kcal mol^−1^]	−1 975 634 (−1 686 818)	−1 768 211	−2 212 345 (−1 923 526)	−2 004 928
Δ*E* [kcal mol^−1^]		−190.3		−192.37

Table 2 summarizes the calculated binding energies of all molecular structures to ^•^NO. Generally, these were calculated *in vacuo* using Equation (1), corresponding to Reaction (1). The calculated Δ*E* in case of hemin complexed with ^•^NO was −190.09 Kcal mole^−1^, which was similar to that in case of H‐Tyra, with slight higher binding affinity in case of H‐Tyros.

(R1) $$\text{Hemin}\left(\text{Fe}\left(\text{III}\right)\right)\;+\text{ NO}\to {\left[\text{Hemin}-\text{NO}\right]}^{+}$$

(1)

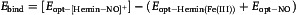




Next, analysis of the NBO charge, natural electron configuration (NEC) and orbital analysis was performed toward understanding of the influence of hemin conjugation to both tyramine and tyrosine residues on the electronic properties and charge contributions of central iron before and after adduction with ^•^NO. In all optimized geometries, the atoms had an overall closed‐shell states under restricted calculations. Upon complexation with ^•^NO, the NPA charge of the central Fe atom decreased owing to the back donation of the unpaired electron in the π*(NO) to one of its d orbitals.^[^ ^36^, ^42^ ^]^ The charge dropped from 0.862 to 0.272 after formation of hemin‐NO complex ([Fe(II)–NO]^+^), similar to the changes in case of both H‐Tyra and H‐Tyros (Table S5, Supporting Information). Similarly, the overall charge of ^•^NO was nearly the same among all tested complexes.

The NEC and natural atomic orbitals (NAOs) were then investigated for a better understanding of the intramolecular interactions upon complexation of each compound with ^•^NO. The calculated NEC in the orbitals of the main atoms in each compound are in Table S6 and S8, Supporting Information. For all complexes with ^•^NO, the total occupation of d‐orbitals in the central Fe is generally higher than that in case of the parent compounds, with equal values among both compound geometries (Table S7, Supporting Information), similar to that of hemin‐NO complex, which reached 6.84. However, the occupancies of the orbitals in N‐atoms of the pyrrole rings decreased following complexation with ^•^NO (Table S8, Supporting Information). Moreover, the electron occupancy in the Fe—N* *σ*‐bonding and charge contributions were the same among all complexes (Table S9 and S10, Supporting Information). Here, two main MOs were formed via *π*‐back bonding interactions between the *d*_*yz*_ and *d*_*xz*_ orbitals of Fe and empty π* orbitals of the NO^+^ ligand. For further analysis of the results, the percentages of the NBO on Fe and N* atoms in the H‐Tyra and H‐Tyros, complexed with ^•^NO were analyzed. These percentages were similar to that of hemin, reported previously.^[^ ^15^, ^41^ ^]^ For instance, in both compounds, these percentages were 64% and 36% on Fe and N* atoms, respectively, in the first bonding orbital (BD2), but were 62% and 38% in the second bonding orbital (BD2), respectively (Table S9 and S10, Supporting Information). Of a note, the calculation of the π*_*d*_*yz*_, and π*_*d*_*xz*_ anti‐bonding orbitals (BD*) confirm these strong interactions between the central Fe and ^•^NO (Table S11 and S12, Supporting Information). Similar charge contributions were reported.^[^ ^34^, ^35^ ^]^

We already reported the details of change in occupancy from the bonding to the antibonding orbitals in the case of hemin.^[^ ^15^, ^41^ ^]^ Here, the electron delocalization in case of H‐Tyra and H‐Tyros showed some similarities to that of hemin, confirming the back‐donation of electrons from d‐orbitals of the metal centre to N* in ^•^NO. However, the electron delocalization was found to be from BD(2) to BD* of Fe–N1 and Fe–N3, and from BD(3) to BD* of Fe–N2 and Fe–N4, with less stabilization energies in case of H‐Tyros complexed with ^•^NO than in hemin and H‐Tyra (Table S13 and S14, Supporting Information). Moreover, the LP*‐NBO orbital displayed several interactions with the BD* orbitals of Fe–N* and Fe–N, which were similar among the different complexes. In addition, some variation in the stabilization energies at the BD_Fe–N_à * BD*_Fe–N*_ interactions were detected, with a higher stabilization in case of H‐Tyra than H‐Tyros and hemin.

Figure S8 and S9, Supporting Information, compare the highest occupied molecular orbital (HOMO) and lowest unoccupied molecular orbital (LUMO) following binding of H‐Tyra and H‐Tyros to ^•^NO, respectively. An increase in the *E*_g_ values was observed in hemin, followed by H‐Tyra and finally H‐Tyros. Moreover, the complexation at the [Fe(II)–NO]^+^ state resulted in a significantly lower energy gap (*E*_g_) between each HOMO/LUMO pair than in the uncomplexed geometries.

### 2.4 Electronic and Magnetic Characterization

**Figure 3:**
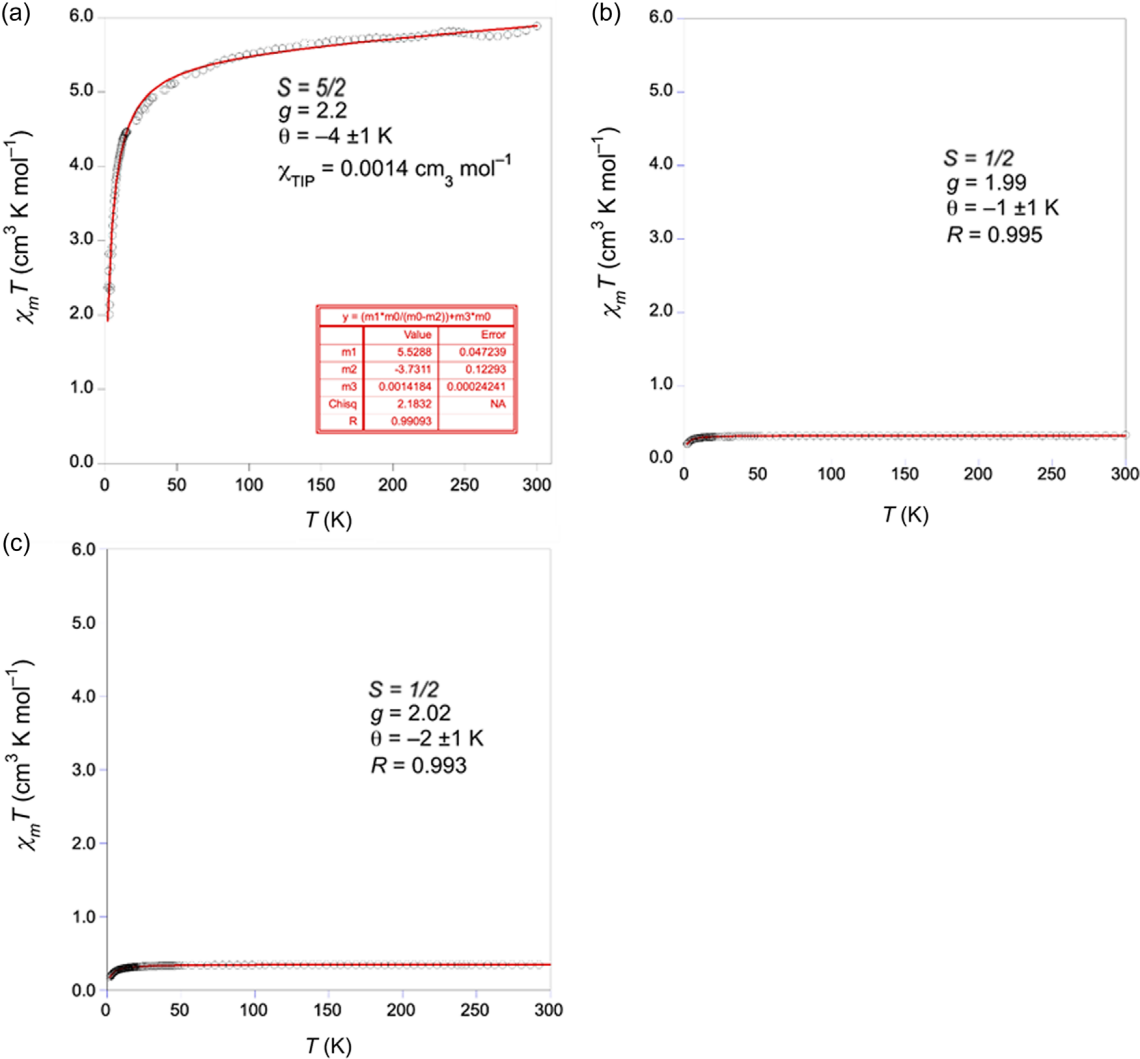
Thermal dependence of the *χ*_*m*_ *T* product for the a) hemin, b) H‐Tyra, and c) H‐Tyros including the parameters extracted from the fitting of the magnetic data.

To gather additional information on the electronic configuration of these complexes, we collected electron spin resonance (ESR) data for H‐Tyra, H‐Tyros, and H‐Dityros and compared with those of hemin. Our findings on the electronic properties of hemin were reported before.^[^ ^15^ ^]^ Here, at 10 K, all compounds showed the typical ESR spectra for low spin Fe(III) hemin complexes, once the data was collected in diluted samples (Table S15, Supporting Information). This confirms that the ground state for all complexes is analogous, with analogous electronic structure. However, at room temperature, the ESR data shows clear differences between them (Figure S10, Supporting Information). Hemin and activated hemin showed highly anisotropic spectra with wide signals, characteristic of a metal‐based spin (Table S15, Supporting Information), which were similar to the results reported before^[^ ^46^ ^]^ and corresponded to the high spin heme‐Fe(III). However, the rest of the series showed more simple spectra, with a dominated isotropic signal near *g* = 2, indicating the participation of an organic radical character, which could be clearly detected in case of H‐Dityros (Figure S10c, Supporting Information). However, the ESR from H‐Tyra and H‐Tyros could not be collected at RT due to their very high‐water content. To investigate their electronic configuration, the magnetic susceptibility data were collected, and compared to that of hemin. As illustrated before,^[^ ^15^ ^]^ the *χ*_*m*_ *T* product for the hemin at room temperature was 5.65 cm^3^ K mol^−1^, which agrees with the Fe(III) high spin center (*S* = 5/2) (**Figure** **3**a). On the contrary, the *χ*_*m*_ *T* product for H‐Tyra and H‐Tyros at room temperature were around 0.37 cm^3^ K mol^−1^, corresponding to a *S* = ½ (Figure 3b,c). These results confirm the organic radical character of these species and the change in their electronic properties after conjugation to tyramine and tyrosine. Moreover, in contrast to the activated hemin species, these final hemin derivatives are characterized by strong charge delocalization, with a dominated organic radical character (*S* = 1/2) and a low spin Fe(II) center (*S* = 0) at room temperature. Collectively, these findings explain the differences in the ^1^H‐NMR spectra of the different compounds.

### 2.5 NO Measurements

**Figure 4:**
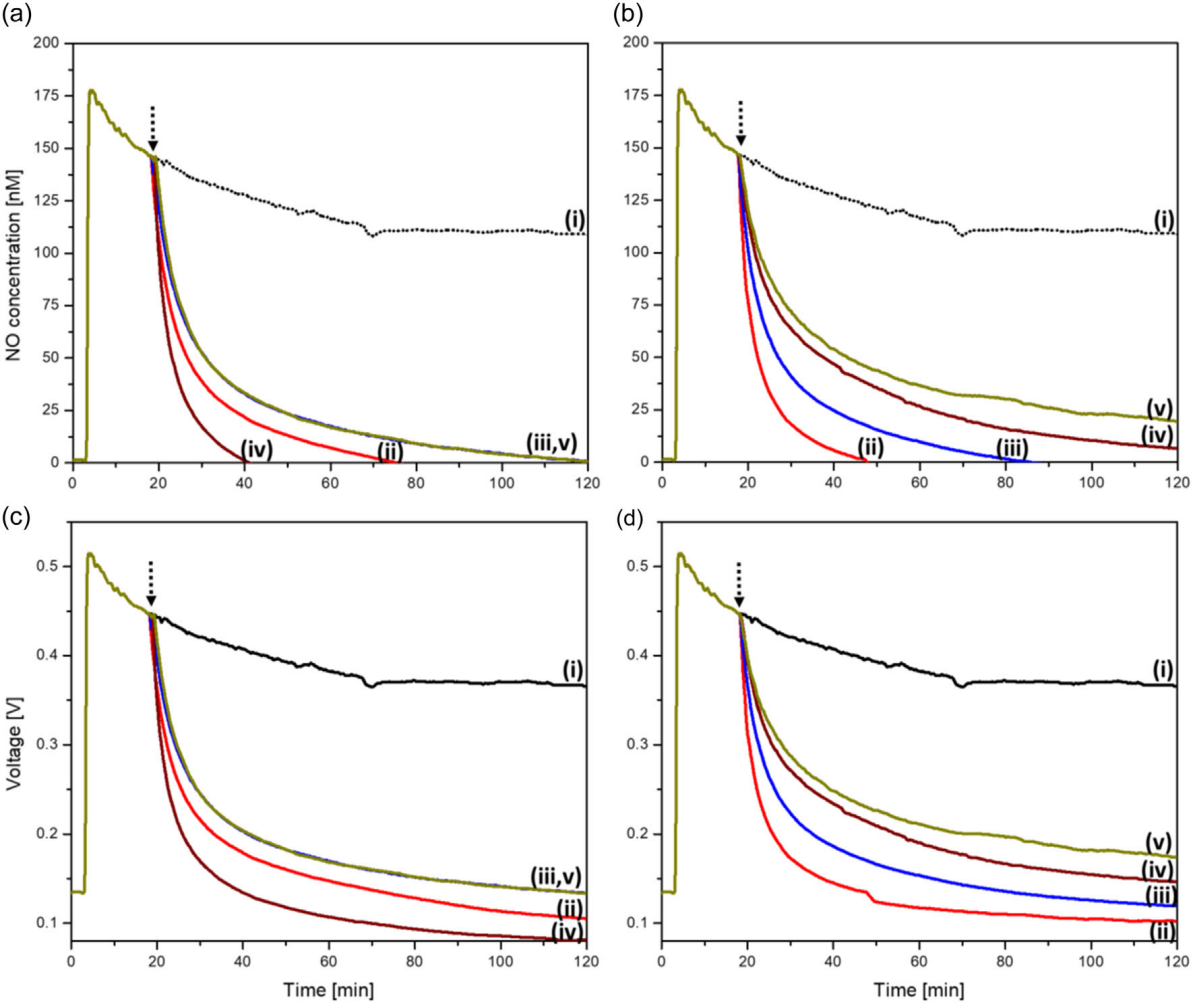
Temporal changes in a,b) ^•^NO levels following the injection of 300 μM DETA‐NO only into FBS‐containing medium (Black colour, i), or after the subsequent addition of hemin (red colour, ii), H‐Tyra (blue colour, iii), H‐Tyros (wine colour, iv), or H‐Dityros (dark yellow colour, v) at the concentrations: a) 4 μM and b) 8 μM, indicated by the short dashed arrow. c,d) The accompanied the voltage signal at 4 and 8 μM, respectively. Results are presented as mean values, *n* = 3.

(Z)‐1‐[N‐(2‐aminoethyl)‐N‐(2‐ammonioethyl)amino]diazen‐1‐ium‐1,2‐diolate (DETA‐NO) was employed as the ^•^NO‐donor, where the degradation of each mol yields 2 moles of ^•^NO, with a reported half‐life of around 20 h at 37 °C in 100 mM phosphate buffer.^[^ ^47^ ^]^^•^NO levels in the culture medium were measured using the electrochemical detection method, where DETA‐NO, once injected causes a rapid increase in the voltage readings. These readings are then translated into measured quantities of ^•^NO using a standard curve. Figure S11, Supporting Information, shows the change in voltage reading corresponding to different added concentrations of S‐nitroso‐N‐acetyl penicillamine (SNAP) and one of the finally generated standard curves employed in detecting ^•^NO in the testing medium. For evaluating the ^•^NO‐scavenging efficiency of the different compounds, they were introduced into the solution after DETA‐NO injection, and this caused a decay in the recorded voltage with a certain rate. Hence, the final ^•^NO‐concentration in solution will depend on the tested compound and its final concentration. We previously reported these changes in the case of hemin.^[^ ^12^, ^15^ ^]^ Variations in the ^•^NO‐scavenging efficiency was observed among the different compounds, with a potential of all compounds to quench the levels of ^•^NO in solution. For instance, H‐Tyros showed the highest efficiency at 4 μM, followed by hemin with similar affinity of H‐Tyra and H‐Dityros toward ^•^NO (**Figure** **4**a,c, Table S16, Supporting Information). However, hemin at 8 μM showed the highest ^•^NO‐scavenging efficiency, followed by H‐Tyra, then H‐Tyros and finally H‐Dityros (Figure 4b,d, Table S16, Supporting Information). The corresponding decrease in mathematical area under the ^•^NO curves is illustrated in Table S16, Supporting Information. The specificity of these changes to the ^•^NO‐scavenging by different compounds was confirmed by recording the voltage signals in the absence of ^•^NO‐donor. Here, all compounds, once injected caused slight changes in the signals within the range of 0–0.008 V, which were nearly constant overtime (Figure S12a–c, Supporting Information), confirming their specific functionality against ^•^NO. Moreover, the release of ^•^NO from DETA‐NO, newly added to incubated culture medium containing each of these compounds, was evaluated. The release was insensitive to hemin solution incubated for 16 h, while the longer incubation periods caused a significant inhibition of ^•^NO‐release (data not shown).

The influence of culture medium components, temperature and the possible aggregation of the different compounds in the aqueous solution on their ability to scavenge ^•^NO was then investigated. Each compound was diluted to 20, 100, and 1000 μM in the FBS‐containing medium and incubated for 16 and 40 h at 37 °C. This was followed by the testing of the scavenging efficiency of the concentration 8 μM from each solution for ^•^NO released from 300 μM DETA‐NO. The ^•^NO‐scavenging efficiency of freshly diluted H‐Tyra in the medium was inhibited with prolonged incubation for 16 h (Figure S13, Supporting Information), similar to our previously reported results with hemin.^[^ ^15^ ^]^ This relates to the enhanced aggregation of hemin in the medium, as explained previously in the dimerization study section. However, some changes were observed, especially at the incubated 20 μM solutions of H‐Tyros, where a decrease in ^•^NO concentration was observed following the injection of 8 μM (Figure S14, Supporting Information). Moreover, H‐Dityros showed a relatively higher ^•^NO‐scavenging in case of incubation at 20 μM (Figure S15, Supporting Information). However, all compounds diluted at concentrations higher than 20 μM did not show any ability to scavenge ^•^NO in the medium. This indicates a probable aggregation, which was proportional to the concentration of these metalloporphyrins molecules, which impact their functionality.

**Figure 5:**
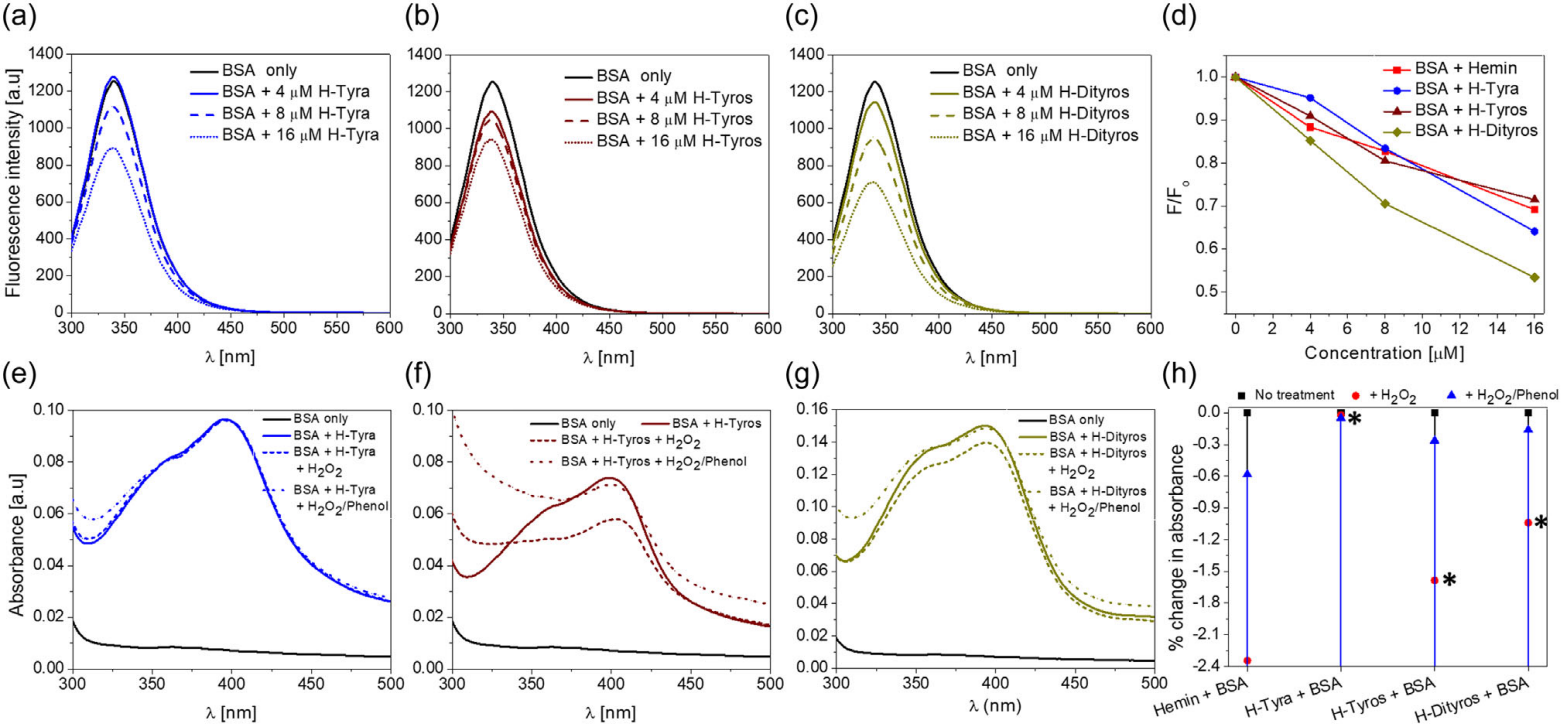
a–c) The fluorescence quenching curves of BSA, mixed with 4, 8 and 16 μM H‐Tyra, H‐Tyros and H‐Dityros, respectively, and incubated at 37 °C for 20 min, and d) shows the accompanied change in the fluorescence intensity of the peak at 340 nm versus the tested concentrations. e–g) UV–vis spectra of BSA before and after mixing with H‐Tyra, H‐Tyros and H‐Dityros, respectively, and incubation at 37 °C for 20 min in the presence/absence of H_2_O_2_ or H_2_O_2_/phenol mixture, and h) shows the accompanied percentage of change in absorbance of the Söret band. Results are presented as mean values, *n* = 3. *, *P* < 0.05 versus the hemin/H_2_O_2_‐containing group using a two‐tailed unpaired student *t*‐test.

One of the probabilities for the observed drop in the ^•^NO‐scavenging ability of these compounds relates to the interaction of their molecules with the serum components of the culture medium. To test that, each compound was mixed with BSA, and the fluorescence was measured after incubation at 37 °C for 20 min. Following excitation at 279 nm, the maximum fluorescence was detected at 340 nm, corresponding mainly to the tryptophan residues in the BSA structure.^[^ ^48^ ^]^ However, dependent on the type and concentration, the mixing of each compound with BSA caused a decrease in the fluorescence intensity with different levels (**Figure** **5**a–c). H‐Dityros showed the highest quenching effects of fluorescence, compared to the other compounds (Figure 5d), indicating its highest affinity toward the indole groups in the BSA structure. Moreover, at equimolar concentrations of BSA and each compound, BSA protected the bound molecules against the oxidative effects of H_2_O_2_, with the highest effects in case of H‐Tyra, followed by H‐Dityros, and finally H‐Tyros (Figure 5e–g). These protective effects were generally higher than those in the case of BSA–hemin interactions (Figure 5h), and they relate mainly to the higher hydrophobic properties of the other compounds. Similar observations were reported before.^[^ ^49^ ^]^ In addition, considering the functionality of phenol to donate electron density via the resonance effect, the peroxidative catalytic functions of hemin were found to be improved once conjugated to tyrosine and tyrosine dipeptide. Hence, phenol protected these compounds in complex with BSA against the oxidative effects of H_2_O_2_. However, in contrast to the other compounds, H‐Tyra, beside its inhibitory effects for the oxidative actions of H_2_O_2_, it was not sensitive to both H_2_O_2_ and H_2_O_2_/phenol mixtures.

### 2.6 In vitro study

#### 2.6.1 Intracellular ^*•*^ *NO Levels*

**Figure 6:**
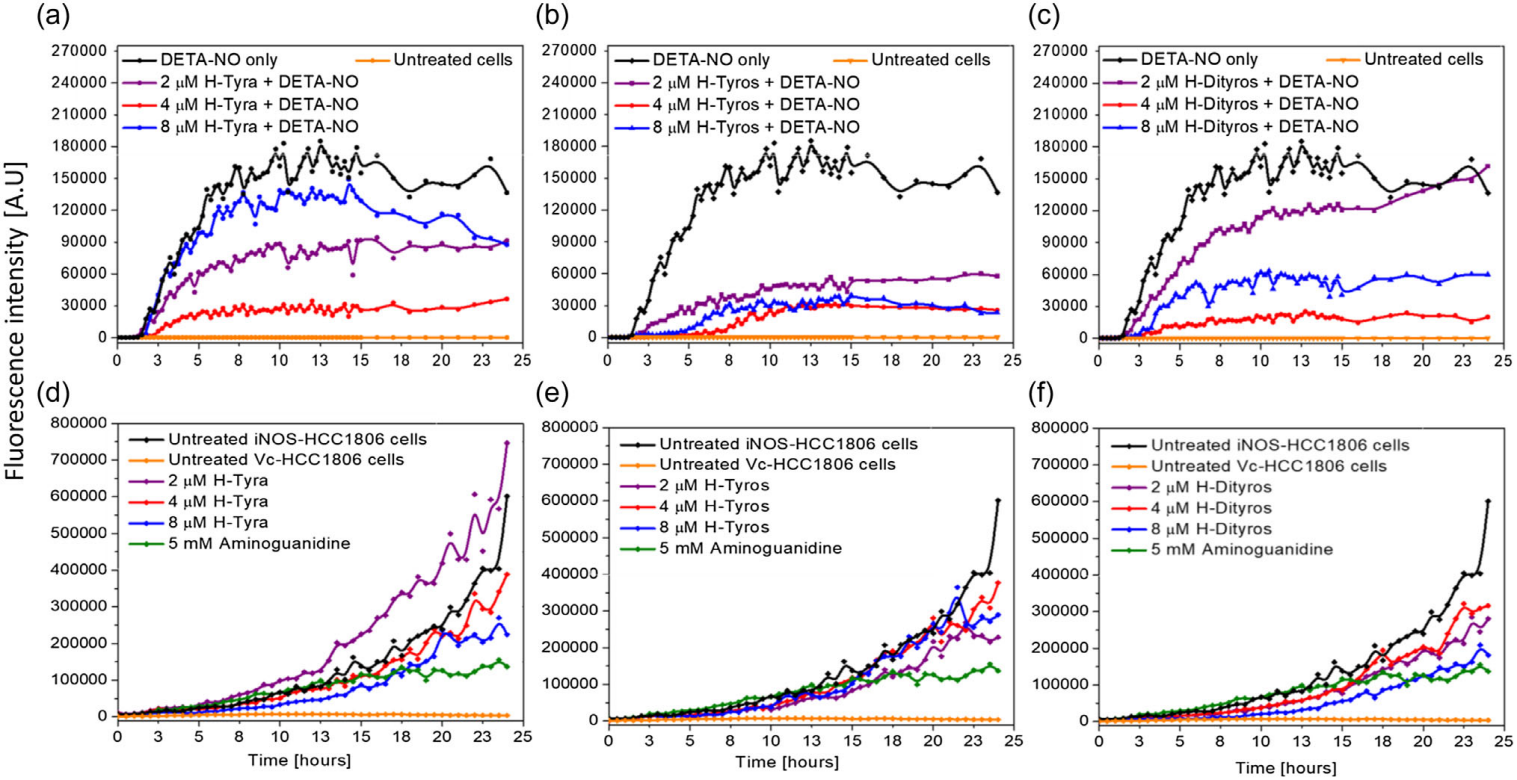
a–c) Kinetics of the changes in levels of intracellular ^•^NO, detected by DAF‐FM‐ fluorescence in MDA‐MB‐231 cells treated with either 300 μM DETA‐NO only (black colour), and/or with either a) H‐Tyra, b) H‐Tyros, or c) H‐Dityros. The DAF‐FM fluorescence in the untreated cells is expressed by the orange colour. d–f) Kinetics of the changes in levels of intracellular ^•^NO in iNOS‐transduced HCC1806 cells treated with either d) H‐Tyra, e) H‐Tyros or f) H‐Dityros. The DAF‐FM fluorescence in the untreated iNOS‐ and empty vector‐transduced cells is shown in black and orange colours, respectively, while the fluorescence of aminoguanidine‐treated iNOS transduced cells is in olive colour. The tested concentrations of compounds were 2 (purple colour), 4 (red colour), and 8 μM (blue colour). Results are presented as mean values, *n* = 3.

We previously reported the anti‐migration effects of hemin on TNBC MDA‐MB‐231, with a detailed study for its molecular effects.^[^ ^13^ ^]^ Here, detection of the intracellular ^•^NO levels was performed using DAF‐FM, as outlined before.^[^ ^14^ ^]^ The MDA‐MB‐231 cell treatment with DETA‐NO caused an enhancement in the intracellular ^•^NO levels, detected by DAF‐FM probe, and the accompanied fluorescence was inhibited in cells treated with the different compounds. Video S1 and S2, Supporting Information, show the changes in untreated and DETA‐NO treated cells, respectively. Similar to the results of the electrochemical detection of ^•^NO, this fluorescence quenching depended on the type and concentration of the tested compound. These changes in the case of hemin were reported before.^[^ ^15^ ^]^ However, here, H‐Tyros, showed the highest efficiency in inhibiting the ^•^NO‐fluorescence intensity compared to the other compounds (**Figure** **6**a–c; Video S3–S5, Supporting Information). Moreover, the increase in concentration of these compounds had a negative impact on the ^•^NO‐scavenging efficiency, except in the case of H‐Tyros, where this efficiency was proportional to its concentration. Video S6–S8, Supporting Information, show these changes in case of cells, treated with 2, 4, and 8 μM H‐Tyros, respectively, while Video S9–S11, Supporting Information, show these changes in case of cell treatment with 2, 4, and 8 μM H‐Dityros, respectively. However, it is noteworthy mentioning that these fluorescence quenching effects were particular to the interactions of these compounds with ^•^NO, where, in the absence of ^•^NO‐donor, slight side effects on fluorescence of the ^•^NO‐probe itself were detected (Figure S16a–c, Supporting Information).

Although the different compounds showed enhancement in the DAF‐FM fluorescence, this was negligible compared to the fluorescence in the presence of DETA‐NO (Figure S41, Supporting Information). A decrease in the concentration of nitrite in the culture medium accompanied the decline in levels of intracellular ^•^NO. This followed the treatment with DETA‐NO in combination with all compounds compared to those treated with DETA‐NO only (Figure S17a–e, Supporting Information). Furthermore, slight differences in nitrite concentrations were observed among the tested concentrations of each compound simultaneously. These occurred where the measured nitrite originates from the remaining uncaptured ^•^NO and nitrite released following the reduction of the nitrosylated complexes. Therefore, the measurement of nitrite concentration indicates the levels of ^•^NO, as a supporting measurement for the ^•^NO‐fluorescence measurements.

While the previous measurements relied on ^•^NO delivered extracellularly, it was important to study the effects of these compounds in case of ^•^NO produced intracellularly. Here, iNOS‐transduced HCC1806 cells were employed, and the DAF‐FM fluorescence was measured following cell treatment with different concentrations of the tested compounds. ^•^NO produced by the iNOS‐transduced cells caused a gradual enhancement in the fluorescence overtime, with kinetics different from that in the case of extracellular application of ^•^NO (Video S12, Supporting Information). In comparison, the enhanced fluorescence in case of vehicle‐transduced cells was relatively negligible, which confirms the specificity of the DAF fluorescence to the intracellular ^•^NO (Video S13, Supporting Information). However, the cell treatment with the different compounds caused changes in the generated fluorescence, with a significant decrease in fluorescence intensity after 15 h of cell treatment. Here, while both H‐Tyra and H‐Tyros showed similar inhibitory effects for the fluorescence generation (Figure 6d,e; Video S14–S19, Supporting Information), H‐Dityros showed the highest fluorescence quenching effects (Figure 6f). For instance, the effects of 8 μM H‐Dityros were similar to those of aminoguanidine, employed as an iNOS enzyme inhibitor (Video S20–S23, Supporting Information). These effects relate to the ^•^NO‐scavenging ability of the different compounds, which are governed by ability of their molecules to permeate through the cell membrane. The cellular uptake of the different compounds will be discussed later.

#### 2.6.2 Cell Migration

**Figure 7:**
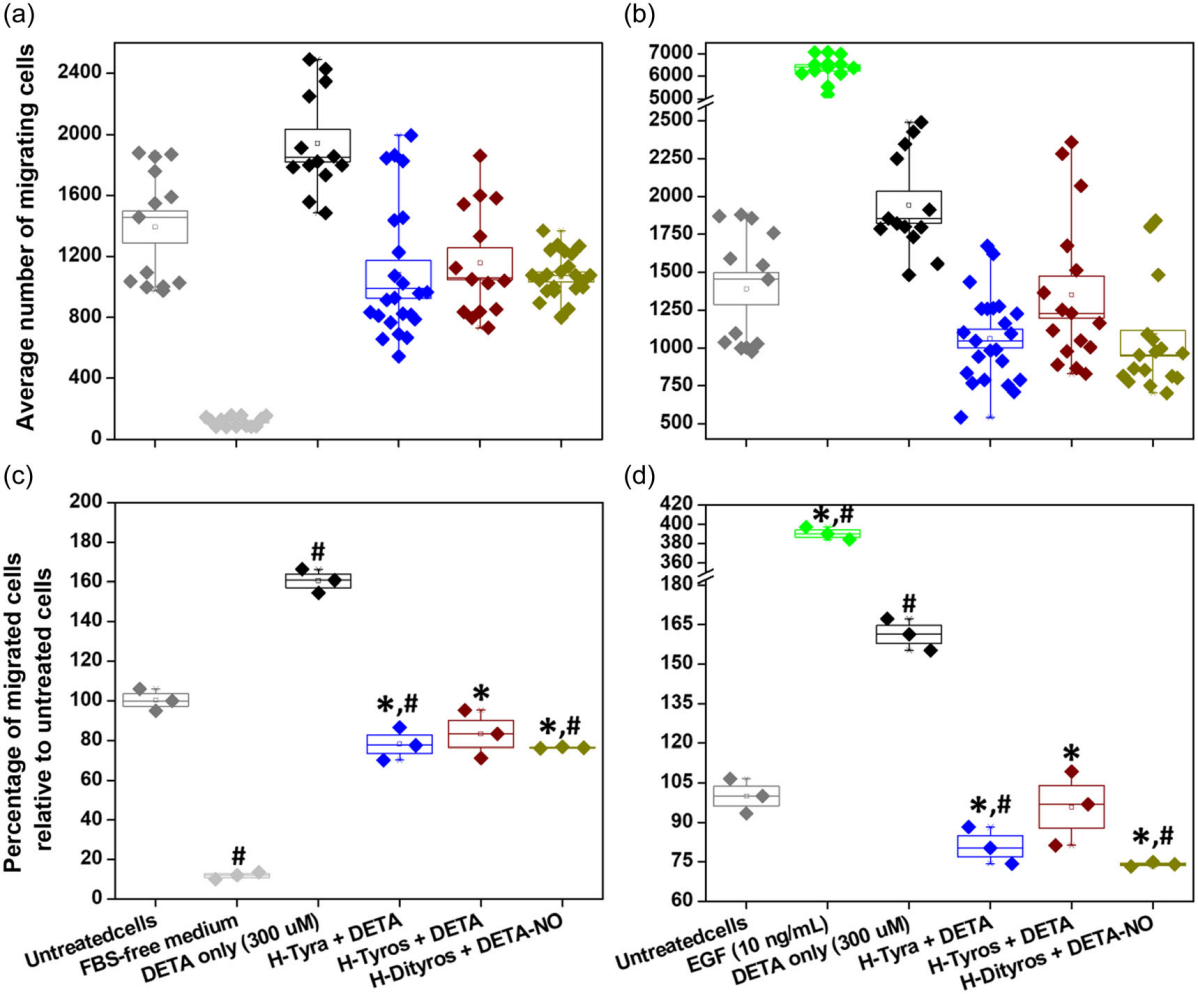
The migration of MDA‐MB‐231 cells through the transwell membranes towards the chemoattractant containing DETA‐NO only (300 μM) or in combination with H‐Tyra, H‐Tyros, or H‐Dityros, tested at the concentrations a,c) 4 and b,d) 8 μM . (a,b) Box‐whisker blot showing the distribution of the counted migrated cells. (c,d) Percentage of migrated cells normalized to the count in the untreated cells (migrated toward the FBS‐containing medium only). Results are presented as mean ± s.d, *n* = 3. ^*,#^, *P* < 0.05 versus the DETA‐NO only‐treated and untreated cells left to migrate for 24 h using a two‐tailed unpaired student *t*‐test.

We previously reported on the influence of varying concentrations of DETA‐NO on MDA‐MB‐231 cell migration, and how hemin can modify these effects through ^•^NO scavenging.^[^ ^13^ ^]^ Here, the influence of ^•^NO from 300 μM DETA‐NO on cell migration was investigated both in the presence and absence of various tested compounds using transwell cell migration and scratch assays. In the transwell assay, cells generally migrate toward the lower side of the membrane because of a chemoattractant. Initially, the effects of different chemoattractant profiles were examined. When FBS‐containing RPMI medium was placed in the lower chamber, it enhanced the migration of MDA‐MB‐231 cells, referred to as untreated cells (**Figure** **7**a,b). However, the absence of FBS in the medium significantly inhibited cell migration (Figure 7a,c). In contrast, the addition of either EGF or DETA‐NO to the lower chamber promoted cell migration to varying extents. Interestingly, the presence of any tested compound in the FBS‐containing medium counteracted the effects of DETA‐NO, leading to inhibited cell migration with differing efficiencies. For example, the tested hemin derivatives substantially inhibited ^•^NO‐induced migration (Figure 7c,d). Furthermore, the inhibitory effects of all compounds were similar at both tested concentrations of 4 and 8 μM.

**Figure 8:**
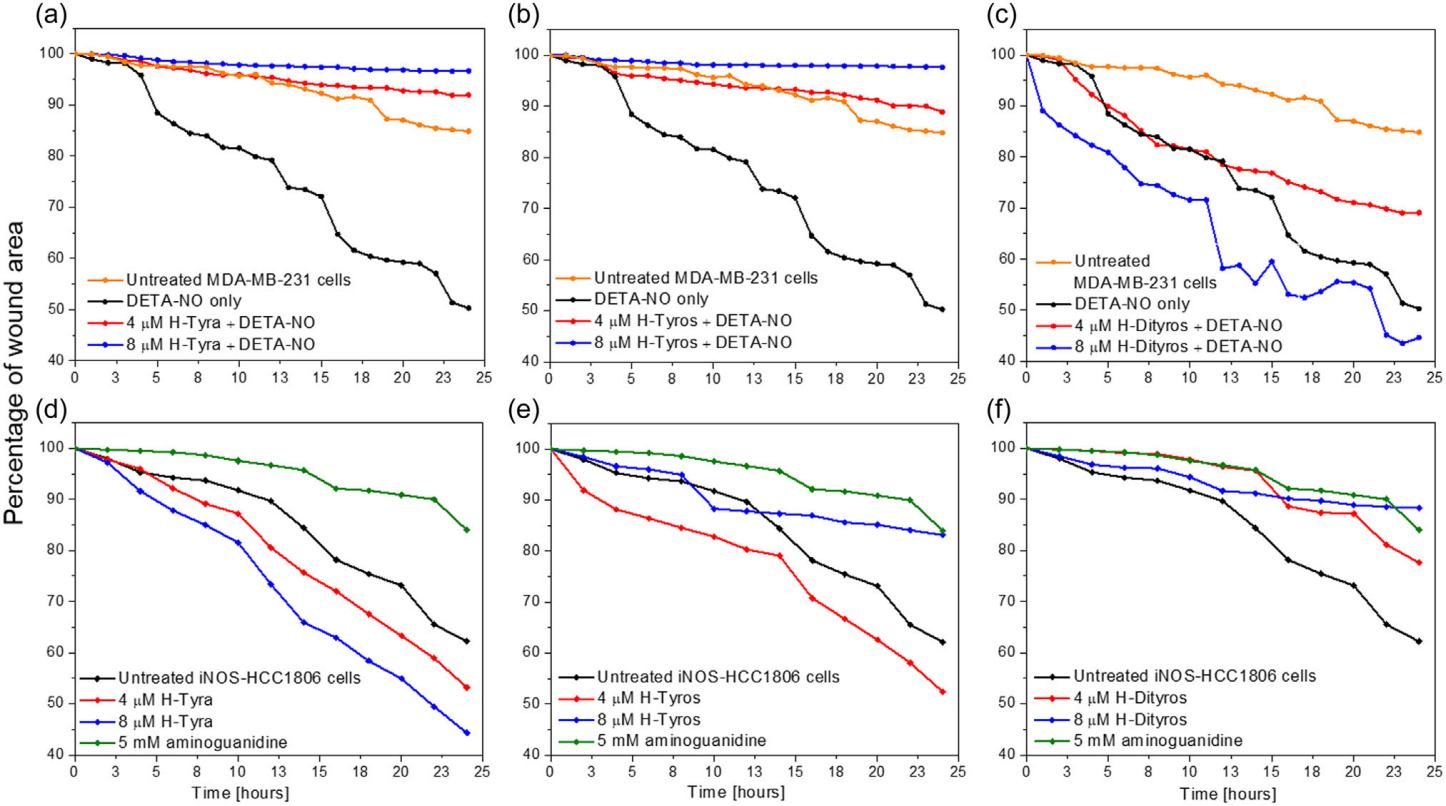
a–c) The effects of DETA‐NO only (300 μM) and in combination with a) H‐Tyra, b) H‐Tyros, or c) H‐Dityros on the migration of MDA‐MB‐231 cell, measured by scratch assay. The orange colour represents the migration of untreated cells. d–f) The effects of d) H‐Tyra, e) H‐Tyros, or f) H‐Dityros on the migration of iNOS‐transduced HCC1806 cells, and compared with those of 5 mM aminoguanidine (olive colour) and untreated cells (black colour). The tested concentrations of compounds were 2 (purple colour), 4 (red colour) and 8 μM (blue colour). Results are presented as mean values, *n* = 3.

In case of scratch assay, DETA‐NO enhanced the cell migration to close the gap generated by scratch generation, while the concomitant cell treatment with either H‐Tyra and H‐Tyros at both tested concentrations inhibited these effects (**Figure** **8**a,b). In contrast, H‐Dityros at 4 μM showed significant inhibition of ^•^NO‐induced cell migration only after 12 h of cell treatment, but with promotion of ^•^NO effects at 8 μM (Figure 8c). However, these effects were different from the behaviour of iNOS‐transduced HCC1806 cells, where H‐Tyra, at both tested concentrations, and 4 μM H‐Tyros enhanced the cell migration (Figure 8d,e). Nevertheless, 8 μM H‐Tyros and H‐Dityros at both tested concentrations inhibited that (Figure 8f). Similar to the effects of these compounds on the intracellular ^•^NO levels, their cellular uptake also plays a role in how they modulate the cell migration and their interactions with certain metabolic pathways. To evaluate that, the intracellular iron was quantified following the treatment of MDA‐MB231 cells with each compound in the presence and absence of ^•^NO, as well as the treatment of iNOS‐transduced HCC1806.

Previously, we reported how ^•^NO could promote the cellular uptake of hemin by MDA‐MB‐231 cells.^[^ ^12^ ^]^ While the levels of intracellular iron were 5.61 ± 0.85 and 3.89 ± 0.25 pmol/10 000 MDA‐MB‐231, treated with 4 and 8 μM hemin, these levels increased to be 5.36 ± 0.75 and 4.67 ± 0.67 pmol/10000 cells, respectively in the presence of 300 μM DETA‐NO, as we reported before.^[^ ^15^ ^]^ Similarly, the levels of intracellular uptake of H‐Tyra, H‐Tyros and H‐Dityros were enhanced significantly in the presence of ^•^NO, with H‐Tyros causing the highest increase in the cellular iron concentration (Table S17, Supporting Information). Moreover, the aggregation of the metalloporphyrin molecules hinders their internalization, which explains the lower iron levels in case of 8 than 4 μM H‐Tyra and insignificant differences at the tested concentrations of H‐Tyros and H‐Dityros. Accordingly, those compounds with more resistance to aggregation will be expected to have a higher affinity toward ^•^NO, which can enhance their cellular uptake, as shown in Table S17, Supporting Information. This is what was observed.

In the case of iNOS‐transduced HCC1806 cells, we reported before intracellular levels of iron of 5.74 ± 2.24 and 3.33 ± 0.78 in cells treated with 4 and 8 μM hemin. However, as shown in Table S18, Supporting Information, the uptake of H‐Tyra was the highest among the tested compounds, with H‐Tyra and H‐Dityros showed similar but lower cellular internalization at 4 μM and a higher uptake level at 8 μM. These results support the previous findings and confirm the higher resistance of hemin derivatives to aggregate, accompanied with enhanced cellular uptake, which also has implications in the scavenging of intracellular ^•^NO and cell proliferation.

#### 2.6.3 Cell Viability

For evaluation whether the effects of the different compounds on cell migration relate to their cytotoxic effects, the metabolic activity of MDA‐MB‐231 cells was measured after 24, 48 and 72 h of treatment with different concentrations of H‐Tyra, H‐Tyros or H‐Dityros. Both H‐Tyra and H‐Tyros compounds maintained the metabolic activity of cells above 80% at all tested concentrations. However, while H‐Dityros showed similar effects up‐to 8 μM, the higher concentration caused a drop in the metabolic activity beginning with the first day of cell treatment (Figure S18a–c, Supporting Information). These results were also confirmed by counting of cells after treatment for 12, 24 and 48 h with different compounds (Figure S19a–c, Supporting Information). Moreover, the results of calcein AM and EthD‐1‐stained MDA‐MB‐231 cells are in Figure S20, Supporting Information.

## 3 Conclusion

Considering the link between poor prognosis in TNBC and iNOS signaling, scavenging intra‐ and extracellular nitric oxide (^•^NO) presents a potential therapeutic strategy for treating TNBC and preventing cancer metastasis. In this article, the synthesis of new hemin derivatives with ^•^NO‐scavenging capabilities was reported. First, detailed synthesis procedures to enhance the properties of hemin in aqueous solution through conjugation to aromatic residues at the terminal –CH_2_COOH via amide bond formation were developed. This modification altered the aggregation behaviour of the resulting derivatives and improved their resistance to oxidative degradation. Next, the ^•^NO‐scavenging efficiency of each compound was evaluated using electrochemical detection. The efficiency varied based on the final compound structure, aggregation behaviour, and their interactions with proteins in solution. Theoretical examinations using the electronic state [Fe(II)–NO]^+^ (*S* = 0) helped elucidate the binding mechanisms between each compound and ^•^NO. Furthermore, the effects of these compounds were studied in vitro by measuring their impact on intracellular ^•^NO levels, cell migration, and cellular uptake. While ^•^NO enhanced TNBC cell migration, its scavenging could be controlled, showing multiple effects depending on whether ^•^NO was introduced extracellularly or intracellularly. Our work provides proof of concept for a new approach targeting cancer cell migration modulation via ^•^NO‐scavenging. Some compounds showed promise for further in vivo studies using appropriate cancer models. Long‐term, and by using these compounds as anti‐metastatic agents following the same methods for hemin modification, we may be able to enhance its ^•^NO‐scavenging efficiency while modulating the physicochemical properties in aqueous solutions. We are currently investigating these approaches, and our focus is testing the in vivo activity of certain hemin derivatives. However, this study did not address the interaction of these compounds with ^•^NO in endothelial cells or their effects on tumour vascularization, which are critical aspects of tumour biology. These areas will be explored in future studies using pre‐clinical models. Additionally, the impact of ^•^NO‐scavenging on tumour‐infiltrating immune cells will be evaluated in subsequent research. Moreover, recognizing that most effective breast cancer treatments rely on combination therapies, the ^•^NO‐scavenging approach could be integrated with chemotherapeutic agents or radiotherapy. The combined effects on TNBC will be further investigated. Finally, evaluating the antitumour efficacy of candidate derivatives in a TNBC tumour‐bearing mouse model is planned to assess their potential biological activity further.

## Conflict of Interest

The authors declare no conflict of interest.

## Author Contributions

A.P. and P.F. conceived and directed the project. A.M.A. preformed the synthesis of H‐Tyra and H‐Tyros, analyzed the structures and properties of the different compounds, carried out the quantum mechanics calculations with P.F. and the in vitro testing with S.A.G. D.C. preformed the synthesis of H‐Dityros. J.W. and Z.W. performed the ESI‐MS and CID experiments for aggregation and degradation studies . J.R.G. performed the magnetic measurements. A.M.A. wrote the manuscript, which was revised by all authors.

## Supporting information

Supplementary Material

## Data Availability

The data that support the findings of this study are available on request from the corresponding author. The data are not publicly available due to privacy or ethical restrictions.
