# Supplementary material for: Facile Synthesis of Hemin Derivatives with Modulated Aggregation Behaviour and Enhanced Nitric‐Oxide Scavenging Properties as New Therapeutics for Breast Cancer
Source: Small Sci. 2024 Jul 16;4(11):2400237. doi: 10.1002/smsc.202400237 (PMC11935252; doi:10.1002/smsc.202400237)
Supplement: Supplementary file 1 — Supplementary Material [file SMSC-4-2400237-s001.zip › smsc.202400237-sup-0001-suppdata-S1.pdf]

Facile Synthesis of Hemin Derivatives with Modulated Aggregation Behaviour and Enhanced Nitric-Oxide Scavenging Properties as New Therapeutics for Breast Cancer

Amir M. Alsharabasy, Durgadas Cherukaraveedu, Jonas Warneke, Ziyan Warneke, José Ramón Galán-Mascarós, Sharon A. Glynn, Pau Farràs Abhay Pandit**

1. Experimental Section

Materials: All solvents involved in the synthesis procedures were dry. hydrochloric acid, acetic acid, DMSO, DMSO-d6, sodium deuteroxide solution (NaOD), pyridine-d5, acetonitrile (MeCn-HPLC grade), formic acid, 1-Ethyl-3-(3′-dimethylaminopropyl)carbodiimide·HCl (EDC.HCl), Diisopropyl carbodiimide (DIC), tyramine, NaCl, Triethylamine.HCl, anhydrous dichloromethane (DCM, peptide grade), DMF (peptide grade), Diisopropylethylamine (DIPEA), thionyl chloride, Chlor-Trt resin (1.1mmol/g as supplied), , Oxyma- pure, ninhydrin, Fmoc-Tyr (t-butl)-OH, ninhydrin, tifluoroactic acid (TFA), triisopropyl silane (TIS), acetonitrile (MeCn- HPLC grade), silica gel (60 A^o, 40-63 µm), thin-layer chromatography (TLC) silica gel sheets (60 A^o, 10-12 µm), sephadex G-15, RPMI-1640 medium, Dulbecco's Modified Eagle Medium (DMEM), L-glutamine, fetal bovine serum (FBS), aminoguanidine, ethidium homodimer (EthD-1), penicillin/streptomycin, paraformaldehyde (PFA), 4′,6-diamidino-2-phenylindole (DAPI) and iron assay kit (Sigma, MAK025) were obtained from Sigma-Aldrich. Ethyl acetate, anhydrous diethyl ether, HPLC grade water, anhydrous methanol (MeOH), Invitrogen™ 4-Amino-5-Methylamino-2',7'-Difluorofluorescein Diacetate (DAF-FM Diacetate), Cell-Quant™ AlamarBlue Cell Viability Reagent, and Griess assay kit (G7921), Invitrogen™ calcein AM were supplied by Fisher Scientific. N-Hydroxysuccinimide (NHS) was purchased from Carbosynth. The •NO-donors used during the study were diethylenetriamine NONOate (DETA-NO) and S-Nitroso-N-Acetyl-D,L-Penicillamine (SNAP) (Cayman Chemicals). The 2-well silicone inserts were from Ibidi, while the transwell insert (membrane 8.0 µm pores) were from Cruinn Diagnostics.

Procedures for hemin-tyramine (H-Tyra) conjugation: This was performed according to **Okorochenkov et al.**[1], with some modifications. To solid hemin (100 mg, 0.154 mmol., 1.0 eq.), DMSO (3 mL) was added under nitrogen atmosphere with stirring for 1 hour at room temperature with the exclusion of light. To this solution, EDC (147.6 mg, 0.77 mmol., 5.0 eq.) and NHS (44.4

mg, 0.386 mmol., 2.5 eq.) were added. The mixture was stirred further under nitrogen atmosphere for one hour at room temperature, then tyramine (84.6 mg, 0.616 mmol, 4 eq.) was added with stirring for a further 24 hours under the same conditions as described previously. The final product was precipitated by its addition to a saturated NaCl solution (6 mL), followed by the addition of diethyl ether (24 mL), mixing and vacuum filtration. The precipitates were washed several times by ether. This was followed by two time-washing by double distilled water to remove the precipitated NaCl crystals giving a dark brown solid with some greenish precipitates. After its drying, the solid was dissolved in methanol (100%) and filtrated. The final hemin derivative was purified using a sephadex G-15 column and methanol (100%) as the mobile phase, which was finally removed *in vacuo*. The final blackish-brown powder was characterized by HPLC, UV-vis, ^{1}H NMR, FTIR, and ESI-MS.

Procedures for synthesis of hemin-N-oxysuccinimide ester: To dried hemin (100 mg, 0.154 mmol., 1.0 eq.), DMSO (3 mL) was added under a nitrogen atmosphere with stirring for one hour at room temperature with the exclusion of light. To this solution, EDC (147.6 mg, 0.77 mmol., 5.0 eq.) and NHS (44.4 mg, 0.386 mmol., 2.5 eq.) were added. The mixture was stirred further under a nitrogen atmosphere for one hour at room temperature. The final product was precipitated by its addition to a saturated NaCl solution (6 mL), followed by adding diethyl ether (24 mL), mixing and vacuum filtration. The dark brown precipitates were washed several times by ether, followed by two time-washing by double distilled water and vacuum dried. The final blackish-brown powder was characterized by HPLC, UV-Vis, ^{1}H NMR, FTIR, and LC-MS.

Procedures for hemin-tyrosine (H-Tyros) conjugation: To dried hemin-N-oxysuccinimide ester (50 mg, 0.06 mmol., 1.0 eq.), DMSO (2 mL) was added under a nitrogen atmosphere with stirring for 30 min at room temperature with the exclusion of light**.** Tyrosine (100 mg) was dissolved in a solvent mixture of DMSO (900 µL), double distilled water (16.7 µL) and HCl (83.3 µL). Tyrosine solution (855.2 µL, 0.472 mmol., 8.0 eq.) was added to the activated hemin solution, followed by triethylamine.HCl (130 µl, 0.945 mmol, 16 eq.). The mixture was stirred for a further 24 hours under the same conditions as those described previously. The final product was precipitated as explained in the case of H-Tyra derivative, with the same purification procedures. The final dark brown powder was characterized by HPLC, UV-Vis, ^{1}H NMR, FTIR, LC-MS and ESI-MS.

Procedures for hemin-dityrosine (H-Dityros) conjugation: For resin activation, to 200 mg of Chlor-Trt resin (1.1mmol/g as supplied) in a fritted polypropylene tube, dry DCM (3 mL) were added and the resin swelled for one hour at RT. The DCM were drained and washed three times with dry DCM (3X3mL). To the resin, dry DCM (2.5 mL) and thionyl chloride (48uL) were added, and the resin were left in a tube rotator for 45 min under mild rotation. The solvents were drained and washed with dry DCM (5X3mL) and dry DMF (5X3mL) and further with dry DCM (5X5mL). To the activated resin, Fmoc-Tyr (t-butl)-OH (5mol excess, 505.5mg) were added in DMF containing DIPEA (3mol excess). The mixture was left in a tube rotator for three hours under mild rotation. The solvents were drained, and the resin were then washed with dry DMF(5X3mL) and dry DCM(5X3mL). The amino acid loaded resin were then capped with DCM:MeOH:DPEA(7:1.5:1.5) for 20 minutes (3X 20 min). The resin was then washed with dry DMF (5x3mL) and then with dry DCM (5X3mL). Fmoc- deprotection of the resin were performed with 4-methyl piperidine (20%, deprotection reagent) with dry DMF. The resin swelled in DCM after capping were retreated with deprotection reagent (2 mL) for 10 minutes. The resins were then washed with DMF (5x3mL) and then with dry DCM (5X3mL). The deprotection steps repeated two more times. From the final Fmoc- deprotected resin a small portion were then qualitatively assayed for primary amines by positive ninhydrin test.

To the tyrosine loaded resin (150 mg), Fmoc (t-butyl)-OH activated with DIC: Oxyma (1: 0.5) in dry DMF added. The resin was then left in the tube rotator for 60 minutes at RT. The solvents were then drained and washed with dry DMF (5x3 mL) and then dry DCM (5x3 mL). A small portion of the resin were qualitatively assayed for a negative ninhydrin test (no blue color). After confirmation as complete conjugation on the resin, the resin was then capped with dry acetic anhydride: DIPEA (3:2) in dry DMF (3x2 mL) for 10 minutes. The resin was then washed with dry DMF (5x3 ml) and dry DCM (5x3 mL).

The resins from the previous step were Fmoc- deprotected as described before. To the amine terminal of the resin, hemin was conjugated as follows: Hemin (0.5: 1 hemin: Resin-(Tyro)$_2$ mol ratio) was dissolved in dry DMF by slow addition of DMF and stirring under magnetic stirring. To the hemin in DMF, DIC: Oxyma (1:0.5 molar ratio) were added, and activated overnight, with the exclusion of light. The activated hemin was then slowly added into the resin containing di-tyrosine residues and were left overnight under RT, with the exclusion of light. The resin was drained off solvents and washed with dry DMF (5x4 mL) and dry DCM (5x4 mL). A small portion of the resin

was assayed for negative ninhydrin test and confirmed the complete consumption of amines on di-tyrosine on the resin. The resin was then cleaved by TFA: TIS: water mixture (95: 2.5:2.5 v/v) for 30 minutes. The excess TFA were removed under reduced vacuum and to the remaining residue, ice cold ether was added to precipitate the peptide conjugated hemin. The precipitate was collected by centrifugation (10,000 g for 10minutes at 4 ^{o}C). The precipitate collected were washed five times with cold ether. The final product were then purified by preparatory HPLC using MeCN: Water eluents (5% MeCN (5 minutes), 5%-95% MeCN (5minutes-30minutes), 5% MeCN (30minutes-40minutes). The fractions were collected, MeCN was evaporated under reduced pressure and freeze-dried The final blackish-brown powder was characterized by HPLC, UV-Vis, ^{1}H NMR, FTIR, LC-MS and ESI-MS.

Characterization of the synthesized compounds: The ultraviolet-visible absorption spectra (UV-Vis) were obtained using a Biologic MOS-500 spectrometer. Varioskan Flash microplate reader (Thermo Scientific) was employed for the other spectroscopic and fluorometric-based measurements. The Fourier-transform infrared (FTIR) spectra were recorded using an IRSpirit FTIR spectrophotometer in a transmission mode, and the data acquisition was performed using a Labsolutions IR software (version 2.25) (all from Shimadzu, Kyoto, Japan). The ^{1}H NMR spectra were obtained at 500 MHz using a Varian VNMRS500 spectrometer between 80 and -5 ppm with a relaxation delay time of 5 s at room temperature. The chemical shifts (δ) were referenced to their solvent signals and expressed in ppm following conversion to the TMS scale [DMSO-d6 (2.48) and water (3.31)]. The coupling constants (J) were expressed in Hz. Analysis of the synthesized compounds was performed using a Shimadzu, prominence LC-2030 plus HPLC instrument. A Luna® C18(2) column (5 µm particle size, 250 x 4.6 mm; cat. 00G-4252-E0) was used for separation of the analytes in the isocratic mode. The mobile phase was a liquid mixture of A+B (9:1, v/v), where: A, MeOH + DCM (9:1, v/v) and B was 3% acetic acid in HPLC grade water. The flow rate was 1 mL/min and the samples were monitored by absorbance at 220 and 400 nm using a Shimadzu photo diode array detector. The tested samples were dissolved in the mobile phase and filtered through Sarstedt Filtropur S 0.2 Syringe Filters (0.2 μm). A Shimadzu Labsolutions Lite Main software (version: 5.93) was used for data acquisition**.** The LC-MS analysis was conducted on a high-resolution Agilent 6540 Q-ToF mass spectrometer with a dual Agilent Jet Stream electrospray ionization mode. The LC-separation was achieved using a C18 HPLC column with a mobile phase of 100% Acetonitrile containing 0.1% formic acid at a flow

rate of 0.2 mL/min. The MS parameters were set at: capillary voltage 3.5 KV, Fragmentor voltage 150 V, desolvation temperature 350°C and data analysis was performed using Agilent Mass Hunter Software Version B.05.01 (B5125).

ESI-MS: The hemin solutions with concentrations of 10^{-5} – 10^{-6} mol/L were injected using a syringe pump into the inlet of an ion trap mass spectrometer (LTQ Orbitrap XL (Thermo)) Fisher Scientific GmbH, Bremen, Germany) or Bruker Esquire 3000 (Bruker Daltonik, Bremen, Germany) at a flow rate of 3 µL/min. The Collision-induced dissociation (CID) experiments were performed by isolating the ion of interest and subsequently fragmenting it by applying an adjustable AC voltage. Helium was used as collision gas at a typical activation time of 30 ms. Ions with a specific m/z value were isolated using a narrow isolation window of around 1 m/z to unambiguously determine the mass of a neutral loss in a CID spectrum. Adduct formation with background gases within the instruments (e.g. water) were studied by isolating the ions in the ion trap for times up to one second.

Characterization of the magnetic properties: The magnetic measurements were carried out on grained powders or diluted DMSO solutions at room temperature and 10 K with an X-band spectrometer Bruker ELEXYS E580 (~9.3 GHz). Each sample was placed in a gel capsule, with a Quantum Design MPMS-XL SQUID magnetometer (Quantum Design, Inc, San Diego, CA, USA) under an applied field of 1000 Oe at <5 K/min temperature scan rates. Experimental susceptibilities were corrected for a diamagnetic contribution from the sample and sample holder. This contribution was experimentally obtained by fitting the low temperature data (< 40 K) of each sample to a Curie-Weiss law with addition of a diamagnetic term **(equation 1)**:

$$\chi = \frac{C}{(T-\theta)} + \chi_{dia} \qquad \textbf{(Equation 1)}$$

where χ is the magnetic susceptibility, C the Curie constant, T is temperature, θ is the Weiss constant and χ_{DIA} the diamagnetic contribution. The corresponding χ_{DIA} term was corrected for all the data. The $\chi_m T$ data was successfully fitted to a proper a Curie-Weiss law in the whole temperature range:

$$\chi T = \frac{g^2 S(S+1)T}{8(T-\theta)} \qquad \textbf{(Equation 2)}$$

Computational details: All computational calculations were carried out with the Gaussian 16 package[2] using quantum mechanics Density Functional Theory (DFT). This was supported by the computational facilities of the Irish Centre for High-End Computing (ICHEC). The starting

geometry for hemin was obtained from the crystal structure of **Koenig,**[3] and further modified to get the structure of the other derivatives. This was made using Gaussview 6.0.16 software package,[4] which was also used for analysis of the data in addition to Avogadro[5], GaussSum[6], and Chemcraft[7] software packages**.** To eliminate errors during calculations, the molecular geometries of the main hemin and H-Styr structures were optimized *in vacuo* first using the B3LYP hybrid functional[8] together with the 6-31+G(d,p) basis set on all atoms**.** This was followed by another *in vacuo* optimization stage, where iron was described with the LANL2DZ basis set. Here, carbon, oxygen, chloride and nitrogen atoms were described by 6-311+G(d,p) basis set; and 6-31+G(d,p) basis set described the hydrogen atoms. The RMS force criterion was set to 1 x 10^{-5} and the maximum number of SCF cycles was 250, with ultrafine integration grids. Next, the stationary points were confirmed to be minima by calculating their harmonic vibrational frequencies at the same level of theory. Subsequently, the geometries of all compounds were optimized following the exchange of Cl with $^{\bullet}$NO. $^{\bullet}$NO bonded to Fe via Fe-N=O bonds, and the same previous calculation conditions were applied. Following that, single point energy (SPE) calculations were performed at B3LYP/cc-PVTZ levels both *in vacuo* and in water via Polarizable Continuum Model (PCM) using the integral equation formalism variant (IEFPCM). Next, the natural bond orbitals (NBO) calculations for finding the natural atomic charges in all compounds were performed at the B3LYP/cc-PVTZ using the Gaussian NBO Version 3.1.

NO-procedures: The $^{\bullet}$NO-scavenging by the different compounds in deoxygenated FBS-containing DMEM culture medium was evaluated according to our previously detailed procedures.[19] The $^{\bullet}$NO-donor employed was DETA-NO and this was tested at a concentration of 300 μM. Each tested compound, dissolved initially in DMSO, was injected after 20 minutes of DETA-NO injection in medium and the voltage reading was monitored over time. A freshly prepared solution of the $^{\bullet}$NO donor, SNAP containing 0.1 M $CuSO_4$ was employed for the weekly calibration of the $^{\bullet}$NO-sensor as recommended by WPI. The specificity of the voltage signal to $^{\bullet}$NO in solution was validated by two methods: (1) injection of each tested compound without the $^{\bullet}$NO-donor and over time recording of the signal and (2) injection of oxygen at certain time-points with monitoring the drop in voltage signal. To evaluate the effects of aggregation and temperature on the $^{\bullet}$NO-scavenging efficiency of each compound, they were diluted in the culture medium and incubated for 16 hours at 37 ^{o}C. This was followed by the injection of each one in the testing DETA-NO-containing medium to a final concentration of 8 μM with over time recording of the

voltage signal. The real-time monitoring of •NO was recorded on a ISO-NOP007 micro-sensor using (a) TBR 1025 Free Radical Analyser (World Precision Instruments (WPI) Ltd, USA). (A) PowerLab hardware device (Model: ML870) was employed as the data acquisition system, and data were analysed using LabChart 7 software (ADInstruments, Colorado Springs, USA).

Metalloporphyrin-BSA interactions: Each compound solution was diluted in phosphate buffer (50 mM, pH 7.4) containing 8 μM bovine serum albumin (BSA) and incubated in the dark at 37 °C for 20 min. This was followed by recording both the fluorescence spectra within the range 300-500 nm following excitation at 279 nm, and the accompanying UV-Vis spectra. Next, the reaction mixtures contained 8 μM of both BSA and each compound, in addition to 320 μM H_2O_2 in the presence/absence of 3.2 mM phenol as detailed previously,[19] with recording the UV-Vis spectra**.** Finally, and for comparison, these procedures were repeated in the absence of BSA, with the same previous measurements.

Detection of intracellular •NO: The two cell lines MDA-MB-231 and iNOS-transduced HCC1806 cells were employed for these studies. The detection of intracellular •NO and its effects on cell migration followed the main procedures of **Alsharabasy et al.,**[20]**.** In brief, MDA-MB-231 were cultured at a density of 25,000 cells/well in a 48-multiwell plate for 24 hours in phenol red-free FBS-containing RPMI for attachment. The media was then discarded, the cells were washed twice with PBS and 200 μL of FBS-containing medium containing 10 μM of DAF-FM was added to each well, followed by one hour of incubation at 37 °C in 5% CO_2. The probe solution was then discarded, the cells were washed three times with PBS and then fresh phenol red-free, FBS-free RPMI medium containing 300 μM DETA-NO with/without different concentrations of each tested compound was added to each well. The cells were directly imaged every one hour using the IncuCyte® S3 Automated Live-Cell Analysis System for a period of 24 hours, with the random choice of four positions per well for imaging. The assessment of fluorescence corresponding to intracellular •NO was performed automatically by IncuCyte ZOOM via measurement of the green object count for all cells stained green with DAF-FM and their intensity with normalizing the results to the total object count per image. The same procedures were repeated in the case of iNOS-transduced HCC1806 cells without the use of DETA-NO.

Evaluation of cell migration via transwell assay: After overnight incubation of cells in FBS-free RPMI, they were trypsinized, counted and 80,000 cells were seeded onto the upper chamber of each transwell insert in FBS-free RPMI, and placed in a well of a 24-multiwell plate. The cell

migration conditions were optimized, and the final study involved diluting DETA-NO in the FBS-containing medium, which were placed into the bottom well at a final concentration of 300 μM with/without different concentrations of the tested compounds. After 24 hours of culturing, the cells were fixed in PFA, washed and the non-migrated cells were cleaned with a cotton swab. The migrated cells in the lower surface of the membrane were stained with 1 μg/mL DAPI. After washing with PBS, these cells were imaged using an Olympus IX81 Inverted Fluorescence Phase Contrast microscope and counted using ImageJ software.

Evaluation of cell migration via scratch assay*:* 50,000 cells were added to each well of a sterile 24-multiwell plate with a silicone insert placed in the centre of the well and left to attach for 24 hours. Next, the inserts were removed, and the cells were washed with PBS and treated with 300 μM DETA-NO diluted in phenol red-containing and FBS-free RPMI with/without different concentrations of the tested compounds. Each well was then imaged each per hour using the IncuCyte system for a period of 24 hours. The analysis of the wound healing rate used ImageJ software and MiToBo toolbox.[21] Three samples were tested per group, the experiment was performed two times, and the averages of all measurements were taken.

Quantification of cellular uptake of the different compounds: MDA-MB231 cells were seeded in T-75 flasks until a confluency of 80% was reached. These were then treated with FBS-free RPMI containing 4 or 8 μM of each tested compound with/without 300 μM DETA-NO, and cultured for 12 hours. After that, the media were removed, the cells were detached, centrifuged, counted and washed by PBS. Iron quantification was performed using the iron assay kit. For that, the separated cells were vigorously homogenized in the iron Assay buffer, centrifuged at 13,000 g for 15 min at 4 ◦C, and ferric iron in the supernatant was reduced by the supplied iron reducer for 30 min. Following this, an iron probe was added to each tested solution, which was incubated for 60 min at room temperature after which the absorbance of solution was measured at 593 nm using the microplate reader. The total cellular iron concentration was normalized to the cell count under each treatment.

Evaluation of cell viability: The cells were cultured at a density of 25,000 cells/well in 48 multiwell plates for 24 hours. Next, the media were exchanged with FBS-free RPMI containing one concentration of the tested compounds or DETA-NO and the cells were cultured for 72 hours. To measure the metabolic activity of cells after 24, 48 and 72 hours of culture, the media were removed and kept at -20 ^{o}C. Each well was then washed with PBS, and 200 μL of fresh 10%

AlamarBlue™ Reagent in PBS was added to each one, followed by incubation for 3 hours. Using the microplate reader, the fluorescence intensity was measured at an excitation of 550 nm and emission of 590 nm, and the percentage of metabolic activity was calculated by normalizing the fluorescence at each treatment to that of untreated cells. Three samples were tested for each concentration at each time point. For cell counting, the same cell treatment procedures were applied. At specific time intervals, the cells were trypsinized and counted after staining with Trypan Blue. Cell viability was assessed using a live/dead viability assay following the same treatments. After washing, the cells were then incubated with PBS containing 1 μM calcein AM and 2 μM EthD-1 for 20 minutes at 37 °C. Imaging was performed using the Operetta high-content imaging system (PerkinElmer, Waltham, MA). Calcein AM was excited at 494 nm and detected using the Alexa Fluor™ 488 filter, while EthD-1-stained cells were detected using the propidium iodide filter.

Statistical analysis: The results were analysed using the SPSS Computer program (Version: 26). All data were expressed as the means ± S.D. Data were analysed using t-test and the differences were considered Statistically Significant at ($p < 0.05$).

2. Results

H-Tyra derivative

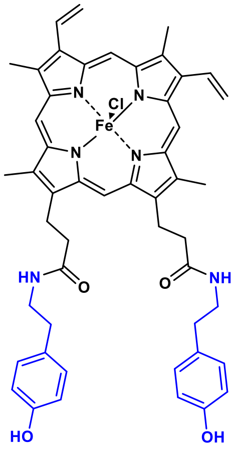

- ^{1}H NMR (500 MHz, DMSO-d6, 25 ^oC, TMS): δ = 9.16 (d, 2H, Ar-OH), 7.89 (d, 2H, 2 =CH- (methine hydrogens)), 6.95 (dd, 4H, Ar-H (meta)), 6.65 (d, 4H, Ar-H (ortho)). The bands corresponding to -CH=CH_2 and -CH=CH_2 are difficult to be distinguished: 5.89 (t, 6H), 5.17 (s, 4H), 2.66 (s, 1H), 2.52 (d, 2H), 2.38 (s, 1H), 2.15 (t, 3H), 2.07 (s, 1H), 1.75 (s, 1H), 1.22 (s, 3H), 1.13 (s, 1H), 0.96 (t, 3H), 0.83 (s, 1H). The solvent traces between 0.7-1.2 refer to diethyl ether
- IR(FTIR): ν`= 3377, 3280, 3009, 2917, 1738, 1642,1551, 1511, 1438, 1373, 1233, 1119, 1015, 947, 830, 704 cm^{-1}
- UV-vis (DMSO): λ_{max}(Ɛ)= 398 (221,029 $cm^{-1}.M^{-1}$)

H-Tyros derivative

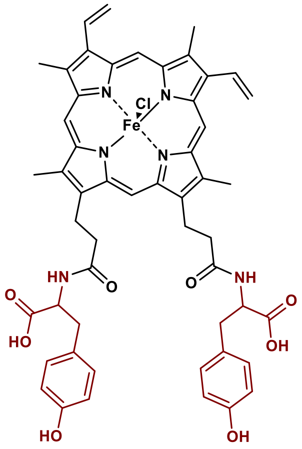

- ^{1}H NMR (500 MHz, DMSO-d6, 25 ^oC, TMS): δ = 10.30 (s, 2H), 9.44 (d, 2H), 8.35 (d, 7H), 7.71 (s, 4H, =CH- (methine hydrogens)), 7.05 (dd, 4H, Ar-H (meta)), 6.71 (p, 4H, Ar-H (ortho)). The bands corresponding to $-CH=CH_2$ and $-CH=CH_2$ are difficult to be distinguished: 5.22 (s, 6H), 4.02 (s, 6H), 3.46 (s, 164H), 3.03 (p, 15H), 2.51 (dd, 99H), 1.90 (dd, 9H), 1.34 – 1.08 (m, 15H). The solvent traces between 0.7-1.2 refer to diethyl ether
- IR(FTIR): ν`= 3420, 3000, 2915, 1742, 1667, 1517, 1431, 1370, 1312, 1218, 1020, 950 cm^{-1}
- UV-vis (DMSO): λ_{max}(Ɛ)= 398 (236,767 $cm^{-1}.M^{-1}$).

H-Dityros derivative

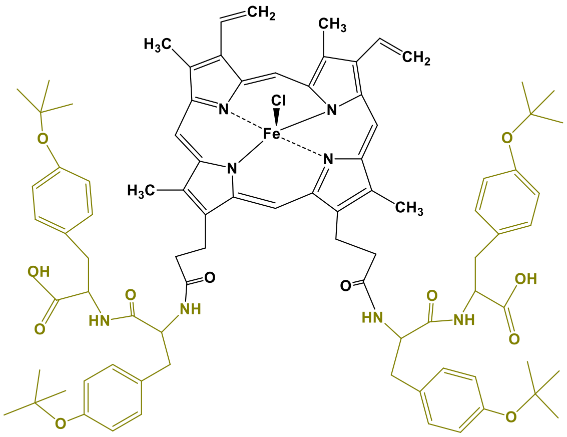

- ^{1}H NMR (500 MHz, DMSO-d6, 25 ^oC, TMS): δ = 7.26 (s, 4H, =CH- (methine hydrogens)), 7.05 (dd, 4H, Ar-H (meta)), 6.71 (p, 4H, Ar-H (ortho)). The bands corresponding to $-CH=CH_2$ and $-CH=CH_2$ are difficult to be distinguished: 5.22 (s, 6H), 4.4 (s, 6H), 1.34 – 1.08 (m, 15H).
- IR(FTIR): ν`= 3280, 2929, 1728, 1641, 1514, 1444, 1367, 1226, 1136, 1021, 942, 830 cm^{-1}
- UV-vis (DMSO): λ_{max}(Ɛ)= 396 (236,767 $cm^{-1}.M^{-1}$).

Tyramine

IR(FTIR): ν`= 3071, 2918, 1744, 1611,1591, 1517, 1492, 1461, 1435, 1354, 1217, 1140, 946 cm^{-1}.

Hemin-N-oxysuccinimide ester

IR(FTIR): ν`= 2931, 2373, 2313, 1783, 1731, 1647.5, 1527, 1426, 1367, 1287, 1201, 1068, 992, 932, 844, 711, 644 cm^{-1}.

Tyrosine

IR(FTIR): ν`= 3615**,** 2935, 2078, 1743, 1579, 1512, 1432, 1364, 1324, 1241, 1157, 1012, 941 cm^{-1}.

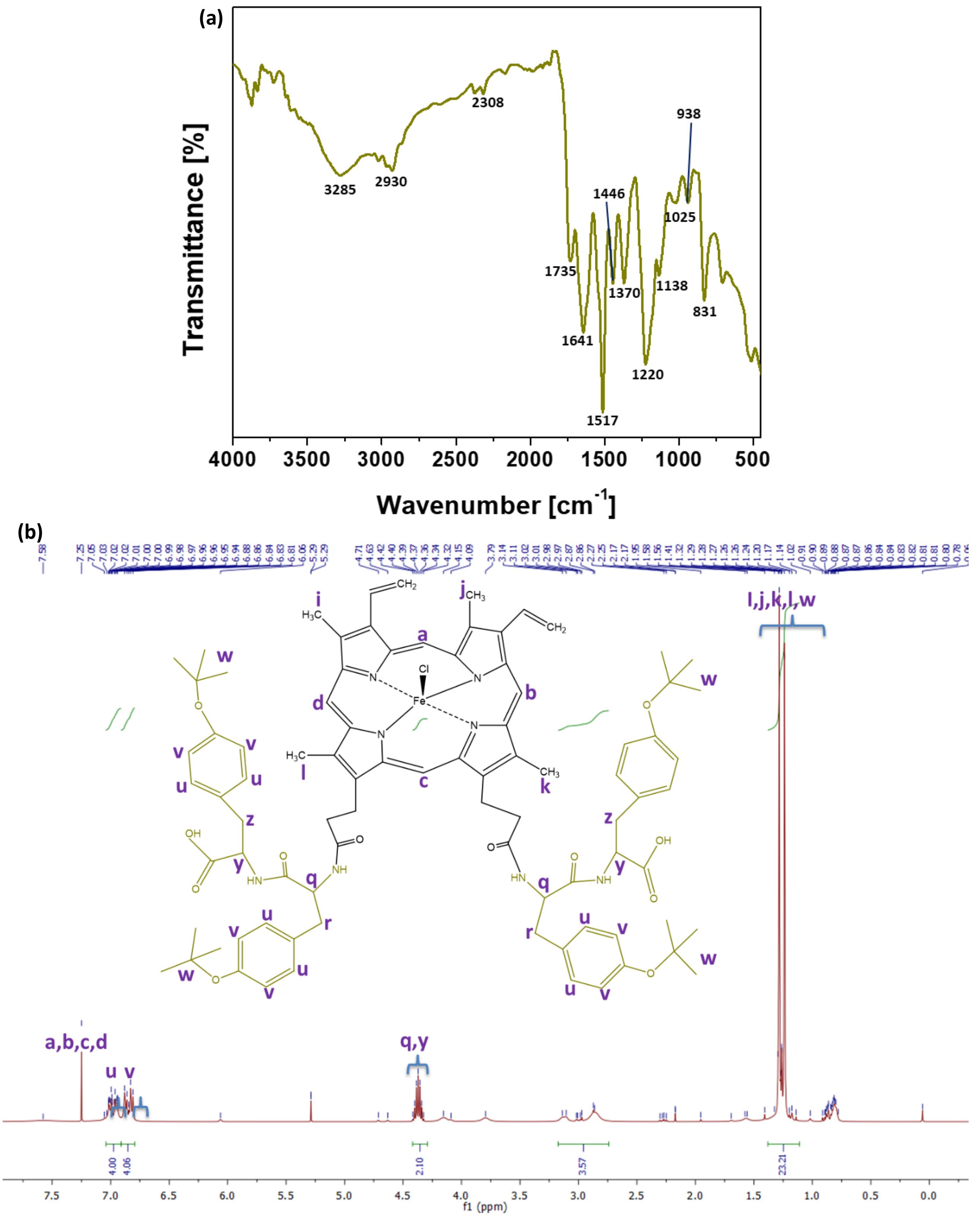

Figure S1. a) ATR-FTIR spectra for H-Dityros. **b)** ^{1}H NMR (500 MHz, DMSO-d6, 25 ^{o}C) spectrum of H-Dityros.

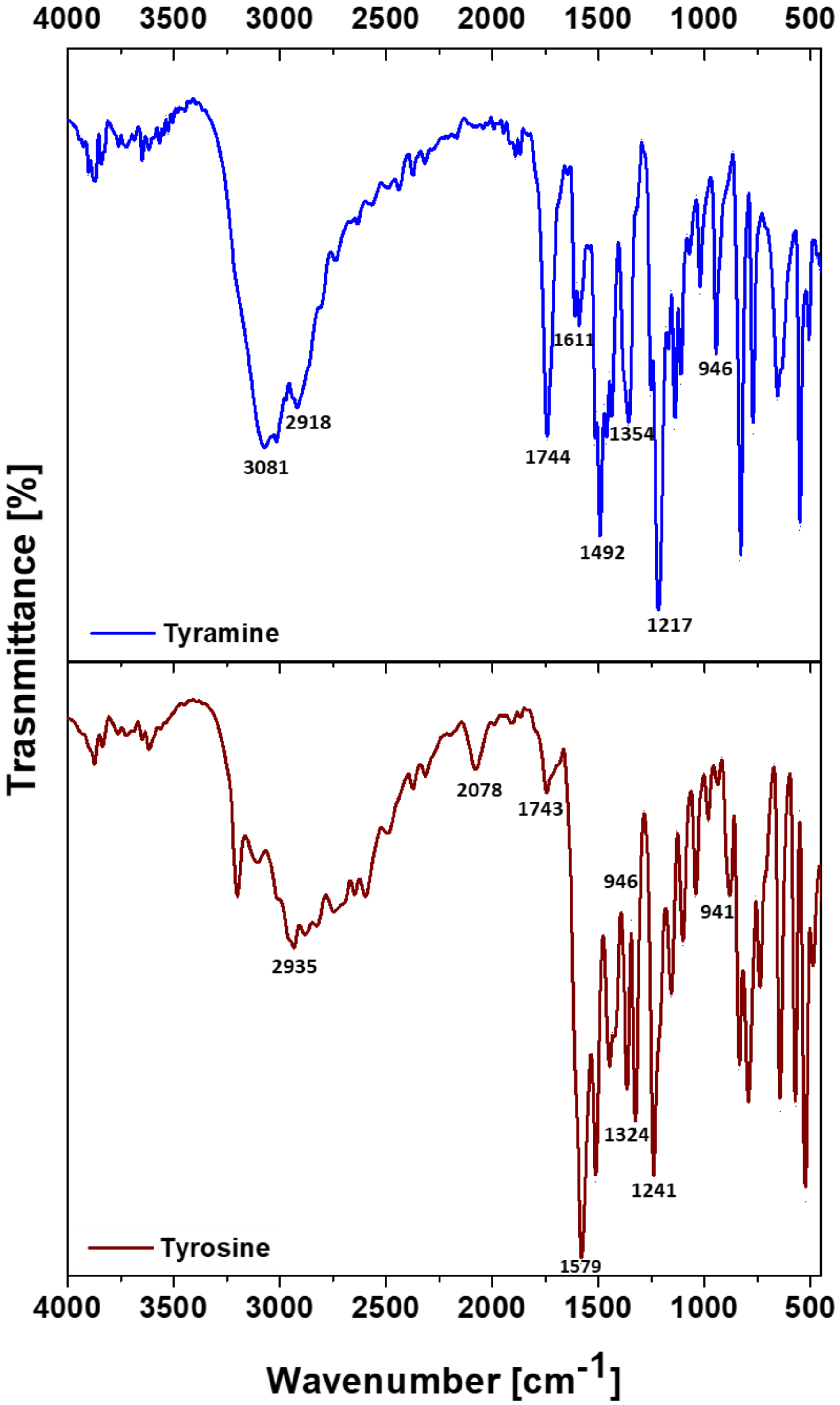

Figure S2. ATR-FTIR spectra for tyramine and tyrosine.

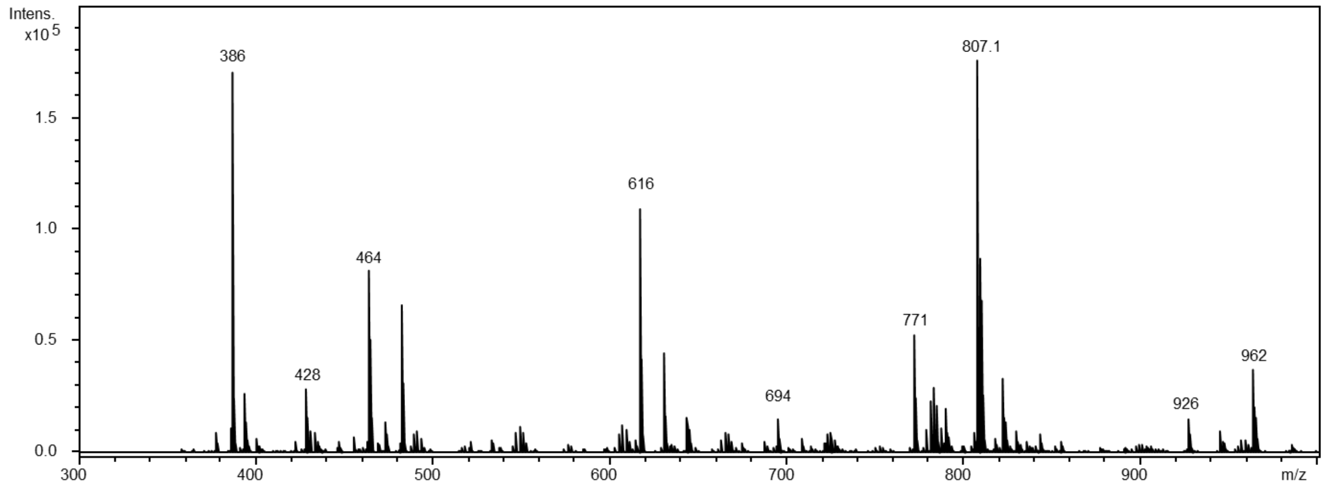

Figure S3. ESI mass spectrum of the H-Tyros sample. The corresponding m/z of H-Tyros was not detected, but instead multiple smaller ions. Their assignment remains unclear.

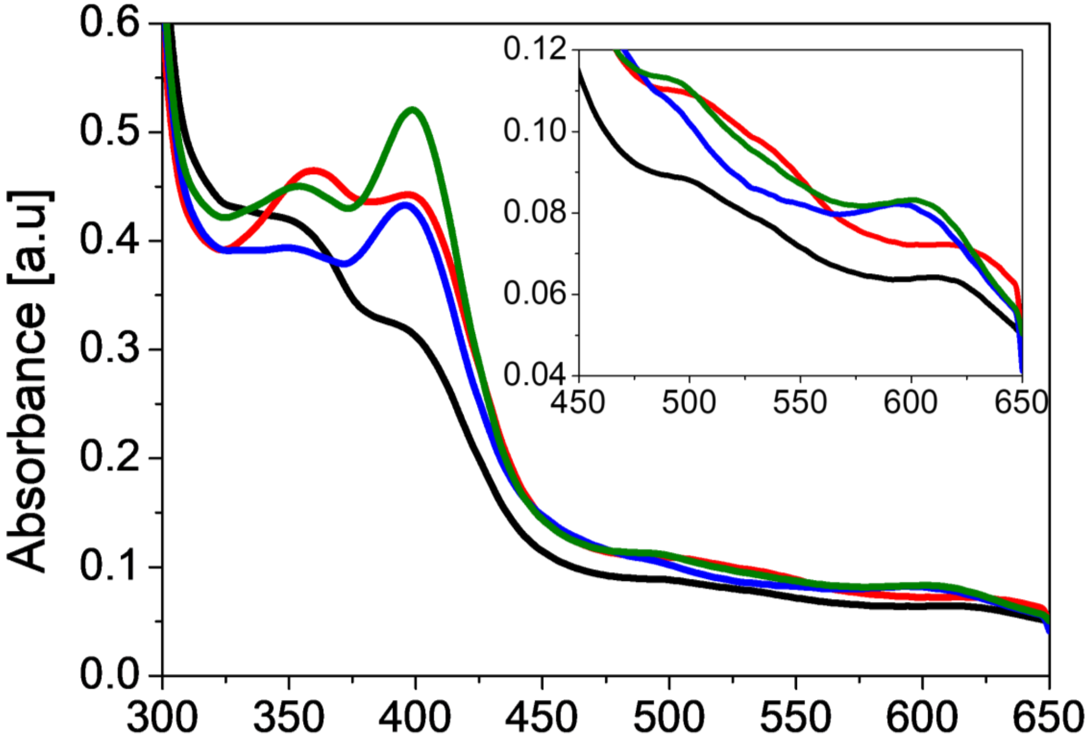

Figure S4. a) UV-vis spectra of H-Dityros in 100% DMSO (green line), FBS-free RPMI (black line), phosphate buffer (red line) and pyridine/NaOH solutions (blue line). Inset: the spectra within the range of 450-650 nm. All measurements were taken after incubation of mixtures at 37 ºC for 20 min. Results are presented as mean, n = 3.

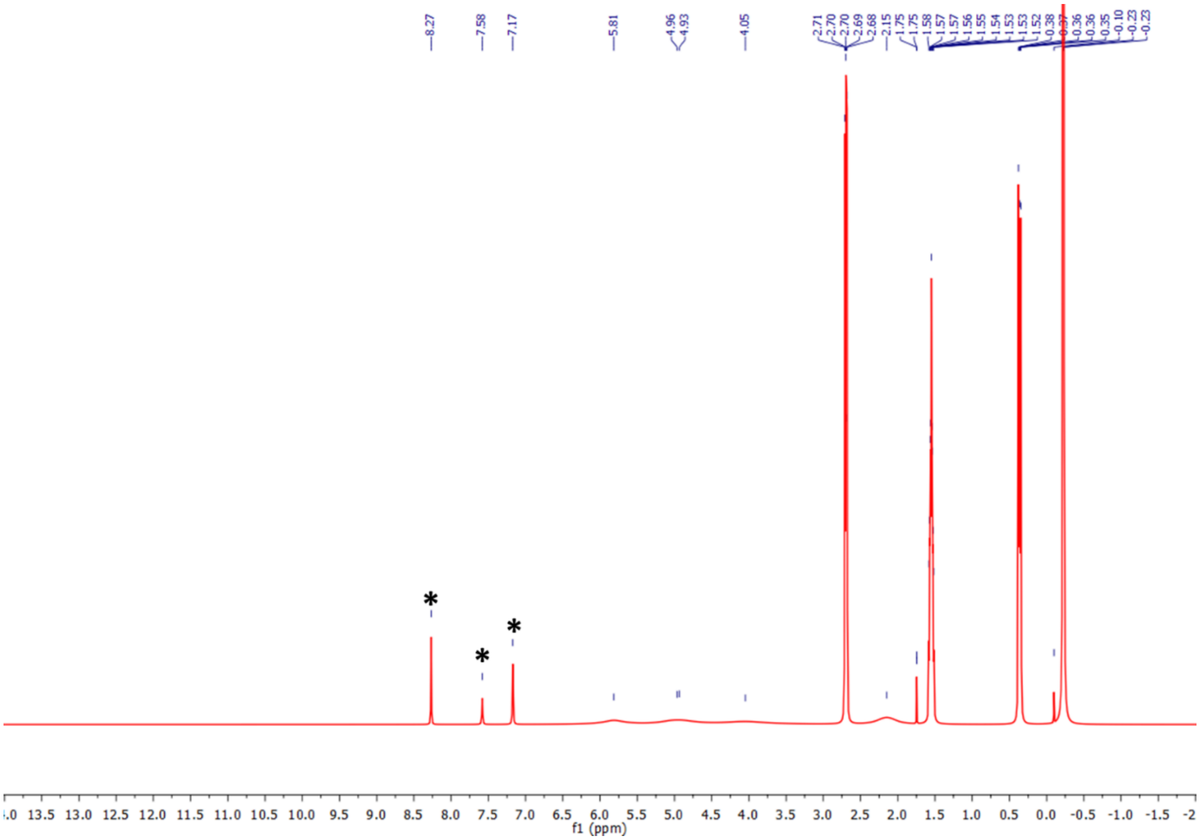

Figure S5. ^{1}H NMR (500 MHz, NaOD/pyridine-d_5/D_2O, 298K) of hemin. D_2O peak was subtracted. Asterisks indicate pyridine-d_5 peaks.

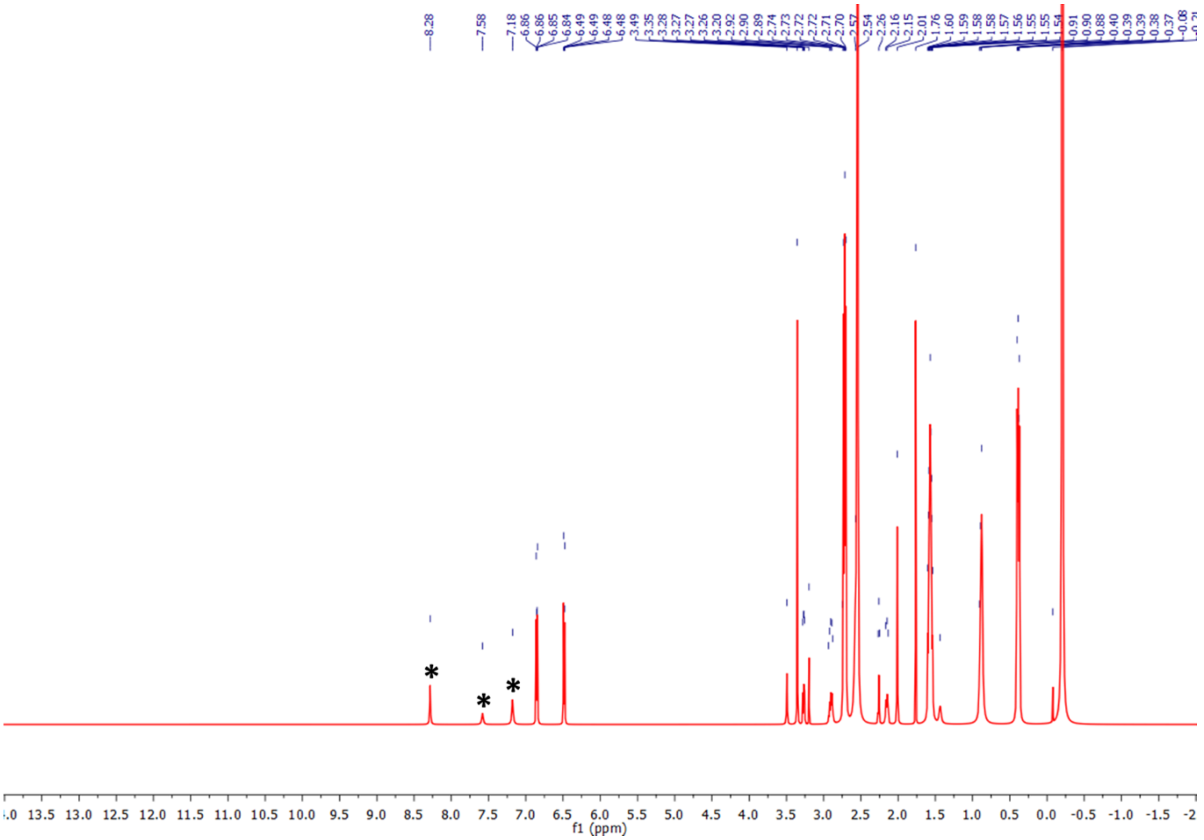

Figure S6. ^{1}H NMR (500 MHz, NaOD/pyridine-d_5/D_2O, 298K) of H-Tyros. D_2O peak was subtracted. Asterisks indicate pyridine-d_5 peaks.

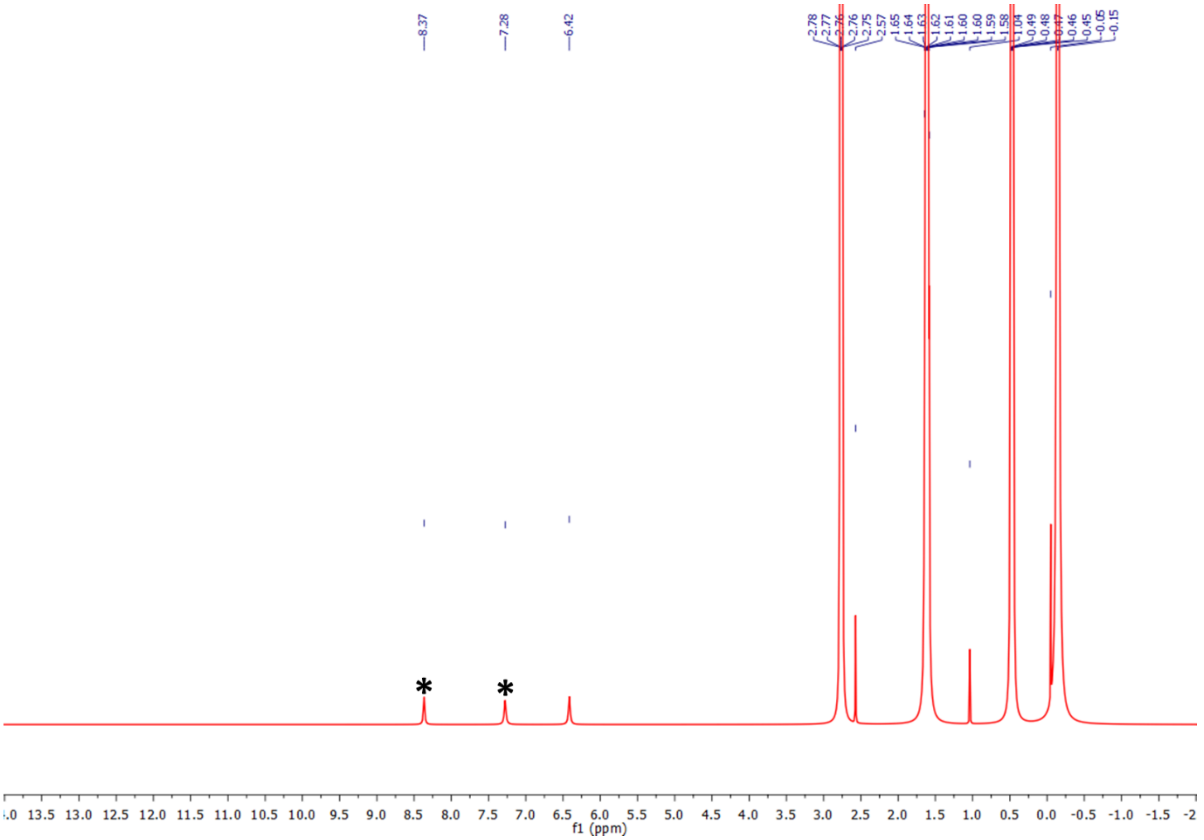

Figure S7. ^{1}H NMR (500 MHz, NaOD/pyridine-d_5/D_2O, 298K) of H-Dityros. D_2O peak was subtracted. Asterisks indicate pyridine-d_5 peaks.

Table S1. XYZ-files of optimized geometry of H-Tyra ($FeC_{50}N_6H_{50}O_4Cl$) (B3LYP, GenECP, S = 3/2). The basis sets: LANL2DZ for Fe atom; 6-31+G(d,p) for H atom**,** 6-311+G(d,p) for C, O, N and Cl atoms.

A summary of the calculation outputs can be accessed on this link:

https://iochem-bd.bsc.es/browse/handle/100/278914

	X	Y	Z
Fe	-3.201067000	0.072884000	-0.032049000
Cl	-2.821683000	-1.674469000	-1.512104000
O	4.596572000	0.305540000	-2.227471000
O	4.281487000	-0.521723000	1.870857000
O	4.724869000	-6.521037000	-3.857599000
O	4.241741000	6.121628000	3.784485000
N	-4.588818000	1.040970000	-1.130320000

N	-1.812016000	1.372604000	-0.715109000
N	-1.903215000	-0.506782000	1.385723000
N	-4.692542000	-0.732718000	1.069207000
N	4.658734000	-2.663847000	1.205926000
N	4.639755000	2.433053000	-1.432176000
C	-5.915748000	0.701288000	-1.238536000
C	0.123671000	0.577122000	0.550472000
C	-0.545786000	-0.304589000	1.377569000
C	0.102212000	-1.118144000	2.384882000
C	-0.891645000	-1.813937000	3.017308000
C	-2.134721000	-1.416285000	2.391190000
C	-3.377852000	-1.864539000	2.789962000
C	-4.576940000	-1.502642000	2.199151000
C	-5.880409000	-1.980001000	2.623210000
C	-6.778654000	-1.535888000	1.680948000
C	-6.027023000	-0.756044000	0.732866000
C	-6.538981000	1.434214000	-2.325416000
C	-6.592841000	-0.120439000	-0.357897000
C	-5.688354000	3.242174000	-3.964133000
C	-0.482521000	3.938235000	-3.116162000
C	1.734107000	2.501168000	-1.223557000
C	2.477975000	1.389832000	-1.997358000
C	1.583277000	-1.149349000	2.621399000
C	-5.571963000	2.271171000	-2.830123000
C	2.356122000	-1.938810000	1.545871000
C	-0.777647000	-2.801533000	4.137539000
C	-8.260280000	-1.748256000	1.640574000
C	-4.364941000	1.993601000	-2.095937000
C	-3.146767000	2.597983000	-2.359240000
C	-1.959831000	2.297887000	-1.722041000
C	-0.685636000	2.896036000	-2.059633000

C	0.245103000	2.304795000	-1.250452000
C	-0.473119000	1.365008000	-0.415651000
C	4.000102000	1.320769000	-1.883190000
C	3.853233000	-1.631192000	1.566165000
C	6.100150000	-2.534659000	1.018439000
C	6.511994000	-2.316408000	-0.451924000
C	6.045689000	-3.418683000	-1.379298000
C	5.131278000	-5.478828000	-3.064675000
C	6.594167000	-4.707281000	-1.314872000
C	5.034720000	-3.183902000	-2.315843000
C	4.577364000	-4.202644000	-3.152316000
C	6.148315000	-5.731510000	-2.142751000
C	-7.917922000	1.322698000	-2.798586000
C	-8.632496000	0.197616000	-2.917138000
C	-6.196475000	-2.777076000	3.806940000
C	-5.645292000	-2.667825000	5.021605000
C	6.082189000	2.491528000	-1.223477000
C	6.494689000	2.264575000	0.245054000
C	5.899730000	3.273371000	1.204683000
C	4.759842000	5.159686000	2.955800000
C	6.319644000	4.611104000	1.205713000
C	4.899566000	2.901052000	2.107702000
C	4.331117000	3.833513000	2.976892000
C	5.761720000	5.550017000	2.065884000
H	1.972399000	3.479488000	-1.651346000
H	1.192256000	0.667238000	0.681374000
H	-3.420963000	-2.564024000	3.611087000
H	-7.654682000	-0.241067000	-0.510428000
H	-0.725588000	3.552393000	-4.111381000
H	0.553219000	4.279569000	-3.142010000
H	-1.113854000	4.814924000	-2.942497000

H	-1.139128000	-3.789736000	3.836740000
H	0.257811000	-2.913291000	4.461335000
H	-1.363902000	-2.492367000	5.008577000
H	2.080130000	2.522254000	-0.185093000
H	1.986445000	-0.134138000	2.644632000
H	1.796079000	-1.578635000	3.603472000
H	-3.119842000	3.343257000	-3.141868000
H	2.112878000	0.408301000	-1.690150000
H	2.247779000	1.467406000	-3.065049000
H	2.173855000	-3.011839000	1.652802000
H	1.991779000	-1.663452000	0.550699000
H	4.221613000	-3.507559000	0.866994000
H	6.571771000	-3.439776000	1.411843000
H	7.605541000	-2.240358000	-0.475878000
H	6.112226000	-1.360346000	-0.796947000
H	7.393341000	-4.916740000	-0.610568000
H	4.612989000	-2.188620000	-2.404786000
H	3.793590000	-3.996376000	-3.875042000
H	6.579298000	-6.723592000	-2.093271000
H	4.025727000	-6.225226000	-4.450577000
H	6.427621000	-1.692150000	1.627709000
H	-8.381241000	2.253700000	-3.116172000
H	-9.648081000	0.224546000	-3.292668000
H	-8.221003000	-0.775987000	-2.680111000
H	-4.891047000	-1.924997000	5.250837000
H	-5.965136000	-3.309625000	5.833298000
H	-6.988847000	-3.509948000	3.676317000
H	4.090377000	3.190983000	-1.059719000
H	6.435851000	3.468105000	-1.567048000
H	7.589364000	2.304400000	0.288227000
H	6.193513000	1.257883000	0.543291000

H	7.106423000	4.927514000	0.528043000
H	4.570669000	1.868140000	2.143070000
H	3.560436000	3.520797000	3.675293000
H	6.094905000	6.580248000	2.067363000
H	3.576480000	5.730752000	4.361068000
H	6.528347000	1.726711000	-1.858890000
H	-5.320198000	4.233706000	-3.684621000
H	-5.110697000	2.910335000	-4.833205000
H	-6.724274000	3.350622000	-4.286745000
H	-8.581711000	-2.442722000	2.417203000
H	-8.580748000	-2.153523000	0.676292000
H	-8.802812000	-0.810243000	1.796946000

Table S2. XYZ-files of optimized geometry of H-Tyra(NO) ($FeC_{50}N_7H_{50}O_5$) (B3LYP/GenECP) based on the $[Fe(II)\text{-}NO]^+$ (S = 0) electronic state. The basis sets: LANL2DZ for Fe atom; 6-31+G(d,p) for H atom, 6-311+G(d,p) for C, O, N and Cl atoms.

A summary of the calculation outputs can be accessed on this link:

https://iochem-bd.bsc.es/browse/handle/100/278912

	X	Y	Z
Fe	-3.303760000	-0.081143000	0.008694000
O	4.484153000	-3.405685000	0.660916000
O	4.167270000	3.603601000	-0.287812000
O	5.360292000	-2.198897000	5.471594000
O	4.728390000	2.266421000	-4.960052000
O	-2.813873000	-0.157601000	2.699214000
N	-4.722932000	-1.495511000	-0.032656000
N	-1.991749000	-1.425533000	-0.657538000
N	-1.984254000	1.343152000	-0.450150000
N	-4.752170000	1.284435000	-0.077094000
N	4.344388000	2.943668000	1.885191000
N	4.334384000	-3.141557000	-1.590023000

N	-3.017424000	-0.128556000	1.584481000
C	-6.024554000	-1.350233000	0.407047000
C	0.014675000	-0.024909000	-0.728595000
C	-0.613381000	1.188682000	-0.541844000
C	0.055106000	2.470223000	-0.545673000
C	-0.929985000	3.418349000	-0.473394000
C	-2.184144000	2.707179000	-0.407812000
C	-3.421073000	3.316421000	-0.363033000
C	-4.623504000	2.650548000	-0.246154000
C	-5.911725000	3.303952000	-0.140350000
C	-6.814413000	2.327565000	0.205069000
C	-6.085721000	1.088699000	0.238592000
C	-6.670489000	-2.641675000	0.537114000
C	-6.664987000	-0.134594000	0.516789000
C	-5.922417000	-5.057381000	0.001572000
C	-0.825608000	-4.903877000	-1.494812000
C	1.483291000	-2.647834000	-1.464902000
C	2.313431000	-2.897020000	-0.188261000
C	1.538421000	2.686771000	-0.608576000
C	-5.758400000	-3.573193000	0.104195000
C	2.186591000	2.732047000	0.786039000
C	-0.792854000	4.909202000	-0.475573000
C	-8.286206000	2.465306000	0.438436000
C	-4.552912000	-2.852078000	-0.209811000
C	-3.379221000	-3.446888000	-0.631713000
C	-2.187103000	-2.785926000	-0.829404000
C	-0.954356000	-3.443831000	-1.188933000
C	0.018236000	-2.482030000	-1.178772000
C	-0.633923000	-1.239812000	-0.834025000
C	3.815810000	-3.162537000	-0.336021000
C	3.667739000	3.126144000	0.723882000

C	5.746826000	3.301748000	2.083603000
C	6.707637000	2.100716000	1.982847000
C	6.353423000	0.950754000	2.901709000
C	5.671224000	-1.192730000	4.597848000
C	6.499410000	1.051445000	4.292783000
C	5.857930000	-0.250988000	2.390708000
C	5.517454000	-1.320187000	3.218019000
C	6.162096000	-0.000868000	5.136646000
C	-8.033731000	-2.903417000	0.999275000
C	-8.655647000	-2.289207000	2.011257000
C	-6.197995000	4.723127000	-0.350114000
C	-5.669896000	5.503174000	-1.299306000
C	5.753383000	-3.354548000	-1.871813000
C	6.606830000	-2.074654000	-1.778694000
C	6.098909000	-0.921629000	-2.618610000
C	5.166613000	1.246517000	-4.159590000
C	6.056271000	-0.990657000	-4.018645000
C	5.660906000	0.261216000	-2.019029000
C	5.199291000	1.342797000	-2.768973000
C	5.593234000	0.072891000	-4.785775000
H	1.607927000	-3.489846000	-2.150765000
H	1.088518000	-0.017736000	-0.847817000
H	-3.453876000	4.395821000	-0.384080000
H	-7.712940000	-0.145997000	0.777547000
H	-1.044918000	-5.518851000	-0.616343000
H	0.184893000	-5.151808000	-1.819037000
H	-1.511030000	-5.209350000	-2.290378000
H	-1.162031000	5.348118000	0.456391000
H	0.248895000	5.207772000	-0.590402000
H	-1.356977000	5.362631000	-1.296000000
H	1.854732000	-1.762718000	-1.990887000

H	2.021015000	1.911835000	-1.208847000
H	1.757758000	3.625510000	-1.118775000
H	-3.387123000	-4.517763000	-0.779239000
H	2.219011000	-2.052405000	0.500331000
H	1.914906000	-3.761853000	0.349210000
H	1.672011000	3.472785000	1.407894000
H	2.076629000	1.770478000	1.298362000
H	3.882988000	2.454601000	2.637258000
H	5.834334000	3.770559000	3.067471000
H	7.715072000	2.469153000	2.205606000
H	6.723944000	1.752076000	0.947240000
H	6.900513000	1.961123000	4.728188000
H	5.745638000	-0.373131000	1.319613000
H	5.150637000	-2.240661000	2.774380000
H	6.290405000	0.077365000	6.208845000
H	5.118496000	-2.993002000	4.982032000
H	6.000621000	4.047614000	1.330382000
H	-8.560533000	-3.699971000	0.481657000
H	-9.664714000	-2.565178000	2.289818000
H	-8.174456000	-1.526716000	2.612582000
H	-4.971534000	5.127629000	-2.037873000
H	-5.959190000	6.542917000	-1.384567000
H	-6.940683000	5.154937000	0.314756000
H	3.763618000	-2.812031000	-2.351049000
H	5.821391000	-3.785625000	-2.873273000
H	7.626532000	-2.342438000	-2.077942000
H	6.659640000	-1.769428000	-0.731359000
H	6.409983000	-1.882411000	-4.526244000
H	5.695634000	0.356198000	-0.939791000
H	4.886873000	2.252440000	-2.264585000
H	5.577671000	0.018941000	-5.867031000

H	4.569688000	3.054157000	-4.427862000
H	6.124550000	-4.093743000	-1.161331000
H	-5.712289000	-5.415320000	-1.010566000
H	-5.247094000	-5.582331000	0.684345000
H	-6.937713000	-5.359435000	0.256141000
H	-8.592375000	3.510461000	0.410663000
H	-8.580287000	2.057156000	1.409750000
H	-8.861520000	1.937167000	-0.328152000

Table S3. XYZ-files of optimized geometry of H-Tyros ($FeC_{52}N_6H_{50}O_8Cl$) (B3LYP, GenECP, S = 3/2). The basis sets: LANL2DZ for Fe atom; 6-31+G(d,p) for H atom**,** 6-311+G(d,p) for C, O, N and Cl atoms.

A summary of the calculation outputs can be accessed on this link:

https://iochem-bd.bsc.es/browse/handle/100/278911

	**X**	**Y**	**Z**
Fe	-3.829209000	-0.260849000	-0.230239000
Cl	-3.817079000	-0.687020000	-2.478474000
O	3.073648000	4.645119000	-1.833672000
O	3.782675000	-0.383699000	-0.939644000
O	5.936113000	-3.134290000	-4.038637000
O	5.847119000	-3.746403000	4.444821000
O	6.555236000	-1.009834000	-3.839755000
O	7.124175000	2.828470000	-1.975425000
O	2.179435000	5.479602000	4.495764000
O	5.843191000	1.013954000	-2.000748000
N	-5.469765000	0.918601000	-0.131998000
N	-2.693604000	1.407204000	-0.229915000
N	-2.190488000	-1.325756000	0.275485000
N	-4.962798000	-1.825709000	0.367413000
N	4.359476000	-2.464951000	-1.655454000
N	3.693396000	2.604767000	-1.024393000

C	-6.772840000	0.516842000	0.028652000
C	-0.480216000	0.411408000	0.064514000
C	-0.886345000	-0.899029000	0.243505000
C	0.019166000	-2.011516000	0.431985000
C	-0.759023000	-3.125118000	0.597351000
C	-2.131934000	-2.682516000	0.495746000
C	-3.222255000	-3.524013000	0.606780000
C	-4.544594000	-3.123576000	0.534656000
C	-5.682723000	-4.003232000	0.731436000
C	-6.796581000	-3.197802000	0.733363000
C	-6.333912000	-1.857845000	0.477217000
C	-7.681358000	1.633150000	-0.158153000
C	-7.175764000	-0.769293000	0.333329000
C	-9.134209000	1.533085000	-0.065979000
C	-9.979762000	2.500419000	0.310820000
C	-7.329881000	4.111614000	-0.812675000
C	-2.090495000	5.052499000	-0.918817000
C	0.561429000	3.293487000	-0.342981000
C	1.357987000	2.971223000	-1.633477000
C	2.782129000	3.500381000	-1.529014000
C	1.514455000	-1.915055000	0.357758000
C	-6.897326000	2.721568000	-0.466639000
C	2.026353000	-2.002804000	-1.104262000
C	3.466370000	-1.550108000	-1.230385000
C	-0.326752000	-4.541567000	0.822139000
C	-5.669445000	-5.455925000	0.901636000
C	-4.900347000	-6.333042000	0.246566000
C	-8.229171000	-3.601818000	0.894170000
C	-5.531148000	2.260316000	-0.432689000
C	-4.433581000	3.080417000	-0.633402000
C	-3.116238000	2.686023000	-0.510867000

C	-1.989739000	3.588812000	-0.618486000
C	-0.869214000	2.840504000	-0.382167000
C	-1.323535000	1.482737000	-0.160321000
C	5.791528000	-2.222194000	-1.830979000
C	6.098063000	-2.163043000	-3.344889000
C	6.635127000	-3.341694000	-1.179352000
C	6.422097000	-3.470752000	0.311720000
C	6.013415000	-3.694515000	3.088143000
C	6.919957000	-2.506567000	1.198939000
C	5.723160000	-4.551898000	0.851554000
C	5.516293000	-4.670788000	2.225795000
C	6.721723000	-2.608210000	2.569661000
C	5.064401000	2.980601000	-0.729880000
C	6.101871000	2.302021000	-1.637922000
C	5.454338000	2.710380000	0.750357000
C	4.578164000	3.430849000	1.750131000
C	2.948037000	4.771120000	3.611187000
C	4.604310000	4.827457000	1.862584000
C	3.719629000	2.724942000	2.594261000
C	2.908653000	3.380867000	3.520713000
C	3.802282000	5.496109000	2.777687000
H	0.599317000	4.372692000	-0.180459000
H	0.581078000	0.613557000	0.093600000
H	-3.031683000	-4.569764000	0.798368000
H	-8.234910000	-0.945738000	0.457555000
H	-2.560407000	5.227739000	-1.891712000
H	-1.105855000	5.520436000	-0.943873000
H	-2.688352000	5.578243000	-0.167472000
H	-0.598738000	-5.184386000	-0.021612000
H	0.754933000	-4.608686000	0.952264000
H	-0.792143000	-4.966999000	1.716222000

H	1.069124000	2.843397000	0.516073000
H	0.889517000	3.462783000	-2.487276000
H	1.858150000	-0.972302000	0.788662000
H	1.977455000	-2.709807000	0.948700000
H	1.889563000	-3.016172000	-1.489860000
H	1.431270000	-1.335589000	-1.733668000
H	-4.621261000	4.120200000	-0.862508000
H	4.013438000	-3.364983000	-1.956983000
H	6.013994000	-1.276269000	-1.344339000
H	7.686180000	-3.123119000	-1.395180000
H	6.408649000	-4.283208000	-1.686183000
H	7.485341000	-1.663066000	0.814582000
H	5.343304000	-5.328193000	0.194865000
H	4.977307000	-5.526214000	2.622640000
H	7.115841000	-1.863977000	3.251035000
H	5.376314000	-4.550329000	4.688411000
H	6.510264000	-0.272364000	-3.198116000
H	3.368453000	1.683815000	-0.758998000
H	5.142460000	4.046439000	-0.942370000
H	6.499141000	3.013505000	0.869997000
H	5.412704000	1.633179000	0.942225000
H	5.261129000	5.407047000	1.222166000
H	3.685007000	1.641371000	2.540261000
H	2.252525000	2.809386000	4.170982000
H	3.826020000	6.575966000	2.859525000
H	1.634368000	4.875698000	5.010870000
H	5.013345000	0.644431000	-1.616284000
H	-9.560669000	0.569215000	-0.332382000
H	-11.048897000	2.324512000	0.328534000
H	-9.642136000	3.478213000	0.631534000
H	-6.654477000	4.573667000	-1.535636000

H	-8.331044000	4.103633000	-1.248210000
H	-7.361910000	4.761615000	0.069239000
H	1.352770000	1.894537000	-1.823735000
H	-6.394005000	-5.848673000	1.612012000
H	-4.982222000	-7.395853000	0.442261000
H	-4.198686000	-6.028359000	-0.521222000
H	-8.312908000	-4.639276000	1.220266000
H	-8.776341000	-3.514472000	-0.050641000
H	-8.744092000	-2.977980000	1.630438000

Table S4. XYZ-files of optimized geometry of H-Tyros(NO) ($FeC_{52}N_7H_{50}O_9$) (B3LYP/GenECP) based on the $[Fe(II)\text{-}NO]^+$ (S = 0) electronic state. The basis sets: LANL2DZ for Fe atom; 6-31+G(d,p) for H atom, 6-311+G(d,p) for C, O, N and Cl atoms.

A summary of the calculation outputs can be accessed on this link:

https://iochem-bd.bsc.es/browse/handle/100/278909

	**X**	**Y**	**Z**
Fe	3.810559000	-0.552982000	-0.062175000
O	-4.304642000	-1.179835000	0.025534000
O	-1.362113000	5.958504000	1.059933000
O	-6.327222000	0.714148000	-0.379322000
O	-6.065275000	-7.673475000	2.739323000
O	3.476530000	-0.236012000	-2.758831000
O	-5.322976000	-1.074814000	-3.087098000
O	-7.498818000	-1.526763000	-2.738581000
O	-3.632132000	8.693930000	0.437114000
O	-2.074327000	8.195662000	-1.104111000
N	4.806574000	-2.289564000	-0.099419000
N	2.147951000	-1.548001000	0.398751000
N	2.890227000	1.109144000	0.521400000
N	5.563078000	0.356963000	0.192060000
N	-2.557246000	5.526672000	-0.813446000

N	-4.502875000	-3.013854000	-1.316344000
N	3.615511000	-0.374866000	-1.642143000
C	6.111155000	-2.462670000	-0.519094000
C	0.578056000	0.325614000	0.462033000
C	1.522549000	1.331206000	0.525741000
C	1.230235000	2.731099000	0.736730000
C	2.438126000	3.363149000	0.870800000
C	3.453714000	2.354076000	0.711096000
C	4.809140000	2.608948000	0.764193000
C	5.796569000	1.674428000	0.535295000
C	7.213459000	1.973522000	0.484870000
C	7.830413000	0.846343000	-0.001158000
C	6.802184000	-0.145850000	-0.164202000
C	6.407777000	-3.869796000	-0.705717000
C	7.045925000	-1.449324000	-0.553455000
C	5.060120000	-6.034530000	-0.281858000
C	0.067868000	-4.642245000	0.883243000
C	-1.601645000	-1.920310000	0.687975000
C	-2.282804000	-2.291936000	-0.643770000
C	-0.120775000	3.372749000	0.855822000
C	5.281191000	-4.554641000	-0.319578000
C	-0.527998000	4.219255000	-0.371798000
C	2.699161000	4.809445000	1.158577000
C	9.289168000	0.620268000	-0.247134000
C	4.290094000	-3.563235000	0.008483000
C	2.982928000	-3.853583000	0.347532000
C	1.981221000	-2.917830000	0.493712000
C	0.592565000	-3.254113000	0.679670000
C	-0.109512000	-2.080516000	0.617435000
C	0.868103000	-1.026669000	0.456780000
C	-3.795993000	-2.105160000	-0.604208000

C	-1.523517000	5.307964000	0.036500000
C	-3.632243000	6.468878000	-0.516689000
C	-4.547262000	6.018261000	0.650345000
C	-5.065688000	4.614710000	0.446325000
C	-5.901644000	1.971764000	-0.081123000
C	-6.166062000	4.353048000	-0.379629000
C	-4.419189000	3.521574000	1.030426000
C	-4.824508000	2.213500000	0.776671000
C	-6.585879000	3.053262000	-0.642326000
C	7.666220000	-4.452123000	-1.171766000
C	8.445090000	-3.971992000	-2.146987000
C	7.862206000	3.227906000	0.866688000
C	7.549850000	3.989035000	1.920841000
C	-5.926532000	-2.921509000	-1.622660000
C	-6.856659000	-2.978866000	-0.382934000
C	-6.634089000	-4.215722000	0.458723000
C	-6.231885000	-6.519026000	2.026174000
C	-7.042454000	-5.479351000	0.010520000
C	-6.025736000	-4.134449000	1.712389000
C	-5.823817000	-5.270843000	2.495771000
C	-6.844432000	-6.621897000	0.775279000
C	-6.183129000	-1.722022000	-2.549726000
C	-3.118500000	7.895834000	-0.300288000
H	-1.997968000	-2.560124000	1.481755000
H	-0.462363000	0.614785000	0.492202000
H	5.122076000	3.626594000	0.945390000
H	8.058518000	-1.713454000	-0.819924000
H	0.160247000	-5.248099000	-0.024213000
H	-0.986137000	-4.626602000	1.161390000
H	0.607939000	-5.161505000	1.679555000
H	3.194678000	5.303498000	0.316595000

H	1.771419000	5.345915000	1.357234000
H	3.344208000	4.931743000	2.033459000
H	-1.888167000	-0.903384000	0.955445000
H	-0.895230000	2.628663000	1.052951000
H	-0.115278000	4.043210000	1.718932000
H	2.710208000	-4.895637000	0.438896000
H	-1.912652000	-1.641919000	-1.444611000
H	-2.026773000	-3.316614000	-0.925805000
H	0.347125000	4.741339000	-0.770339000
H	-0.917626000	3.588222000	-1.175003000
H	-2.688172000	4.899760000	-1.590492000
H	-4.236313000	6.521622000	-1.428679000
H	-5.366401000	6.736381000	0.719756000
H	-3.980421000	6.090669000	1.579071000
H	-6.709782000	5.179877000	-0.825886000
H	-3.589469000	3.696832000	1.707210000
H	-4.316766000	1.377342000	1.243731000
H	-7.437779000	2.860347000	-1.282337000
H	-5.643793000	0.061795000	-0.139296000
H	7.959946000	-5.381552000	-0.692322000
H	9.354300000	-4.485591000	-2.432478000
H	8.188987000	-3.082940000	-2.711383000
H	6.769713000	3.718144000	2.622423000
H	8.102148000	4.896139000	2.130866000
H	8.698845000	3.531631000	0.243846000
H	-3.974800000	-3.697719000	-1.833838000
H	-6.153152000	-3.802040000	-2.232263000
H	-7.881493000	-2.953487000	-0.757887000
H	-6.717953000	-2.083285000	0.220276000
H	-7.545257000	-5.575094000	-0.946864000
H	-5.714690000	-3.168089000	2.093520000

H	-5.362669000	-5.180162000	3.474411000
H	-7.174471000	-7.593594000	0.430738000
H	-5.715576000	-7.477102000	3.615287000
H	4.705089000	-6.362343000	0.699339000
H	4.317931000	-6.346941000	-1.023324000
H	5.980420000	-6.575650000	-0.499203000
H	9.855203000	1.543853000	-0.130612000
H	9.473709000	0.240807000	-1.256139000
H	9.703534000	-0.106223000	0.458867000
H	-7.605925000	-0.751008000	-3.310883000
H	-1.837263000	9.118529000	-0.923777000

Table S5. Comparison of the distribution of the NPA Charges among the optimised geometries of H-Tyra and H-Tyros before and after complexation with •NO based on the $[Fe(II)\text{-}NO]^+$ (S = 0) electronic states at the level of DFT/B3LYP/Gen ECP via Polarizable Continuum Model (PCM) using the integral equation formalism variant (IEFPCM). The basis sets LANL2DZ for the Fe atom, 6-31+G(d,p) for H atom**,** 6-311+G(d,p) for C, O, N and Cl atoms**.**

	**H-Tyra**		**H-Tyros**	
	**Fe(III)**	**$[Fe(II)\text{-}NO]^+$**	**Fe(III)**	**$[Fe(II)\text{-}NO]^+$**
**Fe**	0.833	0.261	0.833	0.258
**N***	-	0.593	-	0.593
**O***	-	-0.068	-	-0.069
**NO***	-	0.525	-	0.524
**N1**	-0.489	-0.406	-0.489	-0.407
**N2**	-0.493	-0.409	-0.490	-0.409
**N3**	-0.488	-0.402	-0.490	-0.403
**N4**	-0.490	-0.409	-0.489	-0.408
**Cl**	-0.484	-	-0.480	-

Table S6. The NEC in the iron atom of the main Fe-porphyrin complexes (S = 3/2) with the occupation of the orbitals in the 3d level.

		NEC	NEC of 3d orbital	Total occupation	T
**H-Tyra**	**α-spin**	$4S^{0.14}3d^{4.42}4p^{0.28}4d^{0.02}$	$(3d_{xy})^{0.783}(3d_{xz})^{0.709}(3d_{yz})^{0.982}(3d_{x2-y2})^{0.978}(3d_{z2})^{0.963}$	$(3d_{xy})^{1.014}$ $(3d_{xz})^{0.983}$ $(3d_{yz})^{1.429}$ $(3d_{x2-y2})^{1.563}$ $(3d_{z2})^{1.309}$	6.298
	**β-spin**	$4S^{0.14}3d^{1.88}4p^{0.27}4d^{0.02}$	$(3d_{xy})^{0.231}(3d_{xz})^{0.274}(3d_{yz})^{0.447}(3d_{x2-y2})^{0.585}(3d_{z2})^{0.346}$		
**H-Tyros**	**α-spin**	$4S^{0.14}3d^{4.42}4p^{0.28}4d^{0.02}$	$(3d_{xy})^{0.574}(3d_{xz})^{0.963}(3d_{yz})^{0.976}(3d_{x2-y2})^{0.919}(3d_{z2})^{0.984}$	$(3d_{xy})^{0.979}$ $(3d_{xz})^{1.116}$ $(3d_{yz})^{1.158}$ $(3d_{x2-y2})^{1.789}$ $(3d_{z2})^{1.257}$	6.299
	**β-spin**	$4S^{0.14}3d^{1.88}4p^{0.27}4d^{0.02}$	$(3d_{xy})^{0.405}(3d_{xz})^{0.153}(3d_{yz})^{0.182}(3d_{x2-y2})^{0.870}(3d_{z2})^{0.273}$		

Table S7. The NEC in the iron, nitrogen and oxygen atoms of Fe(II)-NO^+ (S = 0) with the occupation of the orbitals in the 3d level of iron and 2p level of nitrogen and oxygen.

	NEC in Fe	NEC in N*	NEC in O*
**$[H\text{-}Tyra(NO)]^+$**	[core] $4S^{0.27}3d^{6.85}4p^{0.60}4d^{0.03}$	$2S^{1.31}2p^{3.04}3S^{0.03}3p^{0.02}$	$2S^{1.70}2p^{4.34}sp^{0.01}3d^{0.02}$
	$(3d_{xy})^{0.977}(3d_{xz})^{1.555}(3d_{yz})^{1.554}(3d_{x2-y2})^{1.859}(3d_{z2})^{0.900}$	$(2p_x)^{0.999}(2p_y)^{0.987}(2p_z)^{1.057}$	$(2p_x)^{1.477}(2p_y)^{1.470}(2p_z)^{1.391}$
**$[H\text{-}Tyros(NO)]^+$**	[core] $4S^{0.27}3d^{6.85}4p^{0.60}4d^{0.03}$	$2S^{1.31}2p^{3.04}3S^{0.03}3p^{0.02}$	$2S^{1.70}2p^{4.34}sp^{0.01}3d^{0.02}$
	$(3d_{xy})^{1.174}(3d_{xz})^{1.530}(3d_{yz})^{1.536}(3d_{x2-y2})^{1.674}(3d_{z2})^{0.939}$	$(2p_x)^{1.003}(2p_y)^{0.984}(2p_z)^{1.057}$	$(2p_x)^{1.484}(2p_y)^{1.465}(2p_z)^{1.390}$

Table S8. The NEC in the nitrogen atoms of the four pyrrole rings in the main Fe-porphyrin complexes (S = 3/2) and after binding to •NO at Fe(II)-NO^+ (S = 0) with occupation of the orbitals in the 2p level (in parentheses).

		N1	N2	N3	N4
**H-Tyra**	**α-spin**	$(2p_x)^{0.668}(2p_y)^{0.688}(2p_z)^{0.722}$	$(2p_x)^{0.677}(2p_y)^{0.711}(2p_z)^{0.695}$	$(2p_x)^{0.668}(2p_y)^{0.711}(2p_z)^{0.701}$	$(2p_x)^{0.678}(2p_y)^{0.722}(2p_z)^{0.680}$
	**β-spin**	$(2p_x)^{0.676}(2p_y)^{0.683}(2p_z)^{0.717}$	$(2p_x)^{0.683}(2p_y)^{0.707}(2p_z)^{0.686}$	$(2p_x)^{0.676}(2p_y)^{0.699}(2p_z)^{0.703}$	$(2p_x)^{0.687}(2p_y)^{0.710}(2p_z)^{0.678}$
	**Total Occupation**	$\mathbf{(2p_x)^{1.344}(2p_y)^{1.371}(2p_z)^{1.439}}$ **[4.154]**	$\mathbf{(2p_x)^{1.36}(2p_y)^{1.418}(2p_z)^{1.381}}$ **[4.159]**	$\mathbf{(2p_x)^{1.344}(2p_y)^{1.410}(2p_z)^{1.404}}$ **[4.158]**	$\mathbf{(2p_x)^{1.365}(2p_y)^{1.432}(2p_z)^{1.358}}$ **[4.155]**
**[H-Tyra(NO)]$^+$**		$(2p_x)^{1.314}(2p_y)^{1.336}(2p_z)^{1.431}$ **[4.081]**	$(2p_x)^{1.332}(2p_y)^{1.331}(2p_z)^{1.415}$ **[4.078]**	$(2p_x)^{1.325}(2p_y)^{1.325}(2p_z)^{1.433}$ **[4.083]**	$(2p_x)^{1.322}(2p_y)^{1.327}(2p_z)^{1.425}$ **[4.074]**
**H-Tyros**	**α-spin**	$(2p_x)^{0.692}(2p_y)^{0.653}(2p_z)^{0.734}$	$(2p_x)^{0.649}(2p_y)^{0.692}(2p_z)^{0.738}$	$(2p_x)^{0.691}(2p_y)^{0.651}(2p_z)^{0.738}$	$(2p_x)^{0.650}(2p_y)^{0.692}(2p_z)^{0.737}$
	**β-spin**	$(2p_x)^{0.704}(2p_y)^{0.658}(2p_z)^{0.713}$	$(2p_x)^{0.656}(2p_y)^{0.704}(2p_z)^{0.716}$	$(2p_x)^{0.703}(2p_y)^{0.655}(2p_z)^{0.718}$	$(2p_x)^{0.656}(2p_y)^{0.702}(2p_z)^{0.718}$
	**Total Occupation**	$\mathbf{(2p_x)^{1.396}(2p_y)^{1.311}(2p_z)^{1.447}}$ **[4.154]**	$\mathbf{(2p_x)^{1.305}(2p_y)^{1.396}(2p_z)^{1.454}}$ **[4.155]**	$\mathbf{(2p_x)^{1.344}(2p_y)^{1.410}(2p_z)^{1.404}}$ **[4.158]**	$\mathbf{(2p_x)^{1.306}(2p_y)^{1.394}(2p_z)^{1.455}}$ **[4.155]**
**[H-Tyros(NO)]$^+$**		$(2p_x)^{1.276}(2p_y)^{1.368}(2p_z)^{1.438}$ **[4.082]**	$(2p_x)^{1.375}(2p_y)^{1.278}(2p_z)^{1.428}$ **[4.081]**	$(2p_x)^{1.269}(2p_y)^{1.388}(2p_z)^{1.425}$ **[4.082]**	$(2p_x)^{1.368}(2p_y)^{1.299}(2p_z)^{1.409}$ **[4.076]**

Table S9. The orbital occupancy, hybrids and their percentage in important molecular orbitals of H-Tyramine(NO)$^+$ at the [Fe(II)-NO]$^+$ (S = 0) electronic state, with their energies calculated at B3LYP/cc-PVTZ

**Occupancy (electrons)**	**NBO Type**	**No.**	**Energy (a.u)**	**Atoms**	**Percentage of NBO (%)**	**Polarization coefficient**	**Percentage of character**		
							**S**	**p**	**d**
**1.968**	**BD (1)**	77	-0.884	**Fe**	25.02	0.50	26.71	19.29	53.99
				**N**	74.98	0.87	64.12	35.85	0.02
**1.802**	**BD (2)**	78	-0.349	**Fe**	64.05	0.80	0	19.43	80.54
				**N**	35.95	0.56	0	99.88	0.06
**1.805**	**BD (3)**	79	-0.350	**Fe**	61.70	0.79	0	20.22	79.75
				**N**	38.30	0.62	0	99.89	0.06
**1.979**	**LP**	222	-0.319	**-**	-	-	0	0	100
**0.154**	**BD* (1)**	2563	0.183	**Fe**	74.98	0.87	26.71	19.29	53.99
				**N**	25.02	-0.5	64.12	35.84	0.02
**0.518**	**BD* (2)**	2564	-0.139	**Fe**	35.95	0.60	0	19.43	80.54
				**N**	64.05	-0.8	0	99.88	0.06
**0.503**	**BD* (3)**	2565	-0.131	**Fe**	38.30	0.62	0	20.22	79.75
				**N**	62.44	-0.79	0	99.88	0.06
**0.253**	**LP***	223	0.099	**-**	-	-	4.24	79.87	15.81

Table S10. The orbital occupancy, hybrids and their percentage in important molecular orbitals of H-Tyrosine(NO)$^+$ at the [Fe(II)-NO]$^+$ (S = 0) electronic state, with their energies calculated at B3LYP/cc-PVTZ.

**Occupancy (electrons)**	**NBO Type**	**No.**	**Energy (a.u)**	**Atoms**	**Percentage of NBO (%)**	**Polarization coefficient**	**Percentage of character**		
							**S**	**p**	**d**
**1.967**	**BD (1)**	77	-0.884	**Fe**	25.07	0.50	26.72	19.41	53.86
				**N**	74.93	0.87	64.13	35.84	0.02

**1.795**	**BD (2)**	78	-0.347	**Fe**	63.59	0.80	0	20	79.97
				**N**	36.41	0.60	0	99.88	0.06
**1.811**	**BD (3)**	79	-0.351	**Fe**	62.03	0.79	0	19.76	80.21
				**N**	37.97	0.62	0	99.89	0.06
**1.979**	**LP**	236	-0.319	**-**	-	-	0	0	100
**0.153**	**BD* (1)**	2735	0.185	**Fe**	74.93	0.87	26.72	19.41	53.86
				**N**	25.07	-0.5	64.13	35.84	0.02
**0.516**	**BD* (2)**	2736	-0.136	**Fe**	36.41	0.60	0	20	79.97
				**N**	63.59	-0.80	0	99.88	0.06
**0.504**	**BD* (3)**	2737	-0.132	**Fe**	37.97	0.62	0	19.76	80.21
				**N**	62.03	-0.79	0	99.88	0.06
**0.251**	**LP***	237	0.1	**-**	-	-	4.32	79.76	15.85

Table S11. The NAO coefficients in the bonding and antibonding MOs of Fe-N* following binding of •NO to the different complexes at the $[Fe(II)\text{-}NO]^+$ (S = 0) electronic state.

**Complex**	NBO	**3d-level of iron center**					**2p-level of N* atom**		
		**d_{xy}**	**d_{xz}**	**d_{yz}**	**d_{x2-y2}**	**d_{z2}**	**p_x**	**p_y**	**p_z**
**[H-Tyra(NO)]$^+$**	**BD (1)**	0.077	0.002	0.002	0.012	0	0.042	0	-0.106
	**BD (2)**	0.157	0.006	0.003	0.001	0	0	0	0.370
	**BD (3)**	0.405	-0.017	-0.006	0.003	0	0	0	0.911
	**BD* (1)**	0.077	0.002	0.002	0.002	0	0.042	0	-0.106
	**BD* (2)**	-0.157	0.006	0.003	-0.001	0	0	0	-0.370
	**BD* (3)**	-0.405	0.017	0.006	-0.003	0	0	0	-0.911
**[H-Tyros(NO)]$^+$**	**BD (1)**	0.052	0.002	0.002	0.001	0	0.042	0	-0.073
	**BD (2)**	-0.052	0.002	0	0	0	0	0	-0.105
	**BD (3)**	0.439	-0.018	-0.007	0.003	0	0	0	0.986
	**BD* (1)**	0.052	0.002	0.002	0.001	0	0.042	0	-0.073
	**BD* (2)**	0.052	-0.002	0	0	0	0	0	0.105
	**BD* (3)**	-0.439	0.019	0.007	-0.003	0	0	0	-0.986

Table S12. The NAO coefficients in the bonding MOs of N*-O* of all compounds following binding to •NO based on the [Fe(II)-NO]$^+$ (S = 0) and Fe(III)-NO (S = 3/2) electronic states

**Complex**		**2p-level of N* atom**			**2p-level of O* atom**		
		**p_x**	**p_y**	**p_z**	**p_x**	**p_y**	**p_z**
**[H-Tyra(NO)]$^+$**	**BD**	-0.125	0	0.142	0	0	-0.148
**[H-Tyros(NO)]$^+$**	**BD**	-0.125	0	0.097	0	0	-0.101

Table S13. Estimation of the (bonding-antibonding) interactions between selected molecular orbitals of H-Tyra(NO)$^+$ (S = 0), and determined in the NBO basis at B3LYP/cc-PVTZ:

**Donor (i)**	**Type**	**Acceptor (j)**	**Type**	**E(2) (kcal/mol)**	**E(j)-E(i) (a.u)**	**F(i,j) (a.u)**
**Fe-N***	**BD (2 & 3)**	**Fe-N***	**BD* (2 & 3)**	47.1 & 46.95	0.21 & 0.22	0.097 & 0.098
**Fe-N***	**BD (2)**	**Fe-N1**	**BD***	44.12	0.51	0.134
		**Fe-N2**	**BD***	7.98	0.5	0.057
		**Fe-N3**	**BD***	44.42	0.51	0.135
		**Fe-N4**	**BD***	8.23	0.5	0.057
**Fe-N***	**BD (3)**	**Fe-N1**	**BD***	6.71	0.51	0.052
		**Fe-N2**	**BD***	44.75	0.51	0.134
		**Fe-N3**	**BD***	6.89	0.51	0.053
		**Fe-N4**	**BD***	44.12	0.5	0.133
**Fe-N1**	**BD***	**Fe-N***	**BD* (1)**	94.48	0.02	0.097
**Fe-N2**	**BD***			73.35	0.03	0.095
**Fe-N3**	**BD***			99.5	0.02	0.095
**Fe-N4**	**BD***			72.37	0.03	0.097
**Fe-N***	**BD* (2)**	**Fe-N1**	**BD***	14.73	0.3	0.099
		**Fe-N2**	**BD***	2.66	0.29	0.042
		**Fe-N3**	**BD***	14.48	0.3	0.099
		**Fe-N4**	**BD***	2.69	0.29	0.042

**Fe-N***	**BD* (3)**	**Fe-N1**	**BD***	2.5	0.29	0.041
		**Fe-N2**	**BD***	16.6	0.29	0.104
		**Fe-N3**	**BD***	2.54	0.29	0.041
		**Fe-N4**	**BD***	16.4	0.28	0.103
**Fe**	**LP* (2)**	**Fe-N1**	**BD***	13.75	0.06	0.056
		**Fe-N2**	**BD***	17.37	0.06	0.059
		**Fe-N3**	**BD***	12.98	0.06	0.055
		**Fe-N4**	**BD***	19.55	0.06	0.062
		**Fe-N***	**BD***	49.16	0.08	0.127

Table S14. Estimation of the (bonding-antibonding) interactions between selected molecular orbitals of H-Tyros(NO)$^{+}$ (S = 0), and determined in the NBO basis at B3LYP/cc-PVTZ

**Donor (i)**	**Type**	**Acceptor (j)**	**Type**	**E(2) (kcal/mol)**	**E(j)-E(i) (a.u)**	**F(i,j) (a.u)**
**Fe-N***	**BD (2 & 3)**	**Fe-N***	**BD* (2 & 3)**	49 & 45.5	0.21 & 0.22	0.099 & 0.097
**Fe-N***	**BD (2)**	**Fe-N1**	**BD***	35.13	0.51	0.12
		**Fe-N2**	**BD***	17.93	0.5	0.085
		**Fe-N3**	**BD***	35.53	0.51	0.12
		**Fe-N4**	**BD***	18.38	0.5	0.086
**Fe-N***	**BD (3)**	**Fe-N1**	**BD***	15.65	0.51	0.08
		**Fe-N2**	**BD***	34.77	0.51	0.119
		**Fe-N3**	**BD***	15.97	0.51	0.081
		**Fe-N4**	**BD***	34.17	0.51	0.118
**Fe-N1**	**BD***	**Fe-N***	**BD*(1)**	88.24	0.02	0.096
**Fe-N2**	**BD***			74.37	0.03	0.096
**Fe-N3**	**BD***			91.47	0.02	0.097
**Fe-N4**	**BD***			74.14	0.03	0.098
**Fe-N***	**BD* (2)**	**Fe-N1**	**BD***	12.15	0.3	0.09

		**Fe-N2**	**BD***	6.19	0.29	0.064
		**Fe-N3**	**BD***	11.76	0.3	0.089
		**Fe-N4**	**BD***	6.12	0.29	0.063
**Fe-N***	**BD* (3)**	**Fe-N1**	**BD***	5.79	0.29	0.062
		**Fe-N2**	**BD***	12.68	0.29	0.091
		**Fe-N3**	**BD***	5.79	0.29	0.062
		**Fe-N4**	**BD***	12.61	0.29	0.091
**Fe**	**LP* (2)**	**Fe-N1**	**BD***	14.27	0.06	0.056
		**Fe-N2**	**BD***	17.05	0.06	0.059
		**Fe-N3**	**BD***	14.69	0.06	0.057
		**Fe-N4**	**BD***	19.29	0.06	0.062
		**Fe-N***	**BD***	47.91	0.08	0.127

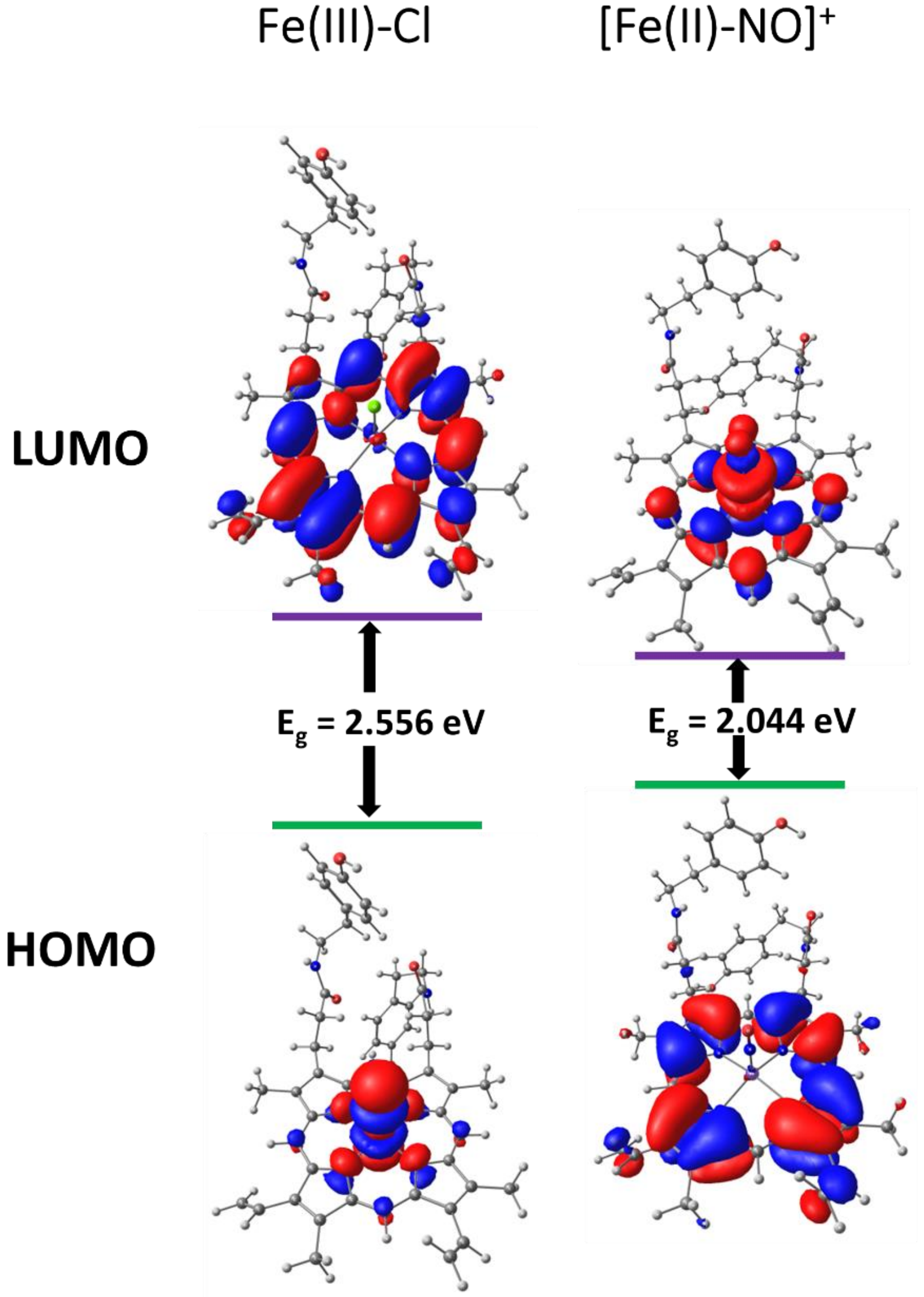

Figure S8. The calculated molecular orbital isosurfaces for different orbitals of the main structure of H-Tyra and following binding to •NO at the $[Fe(II)\text{-}NO]^{+}$ electronic state (isovalue 0.02), and the accompanied E_g using the B3LYP functional.

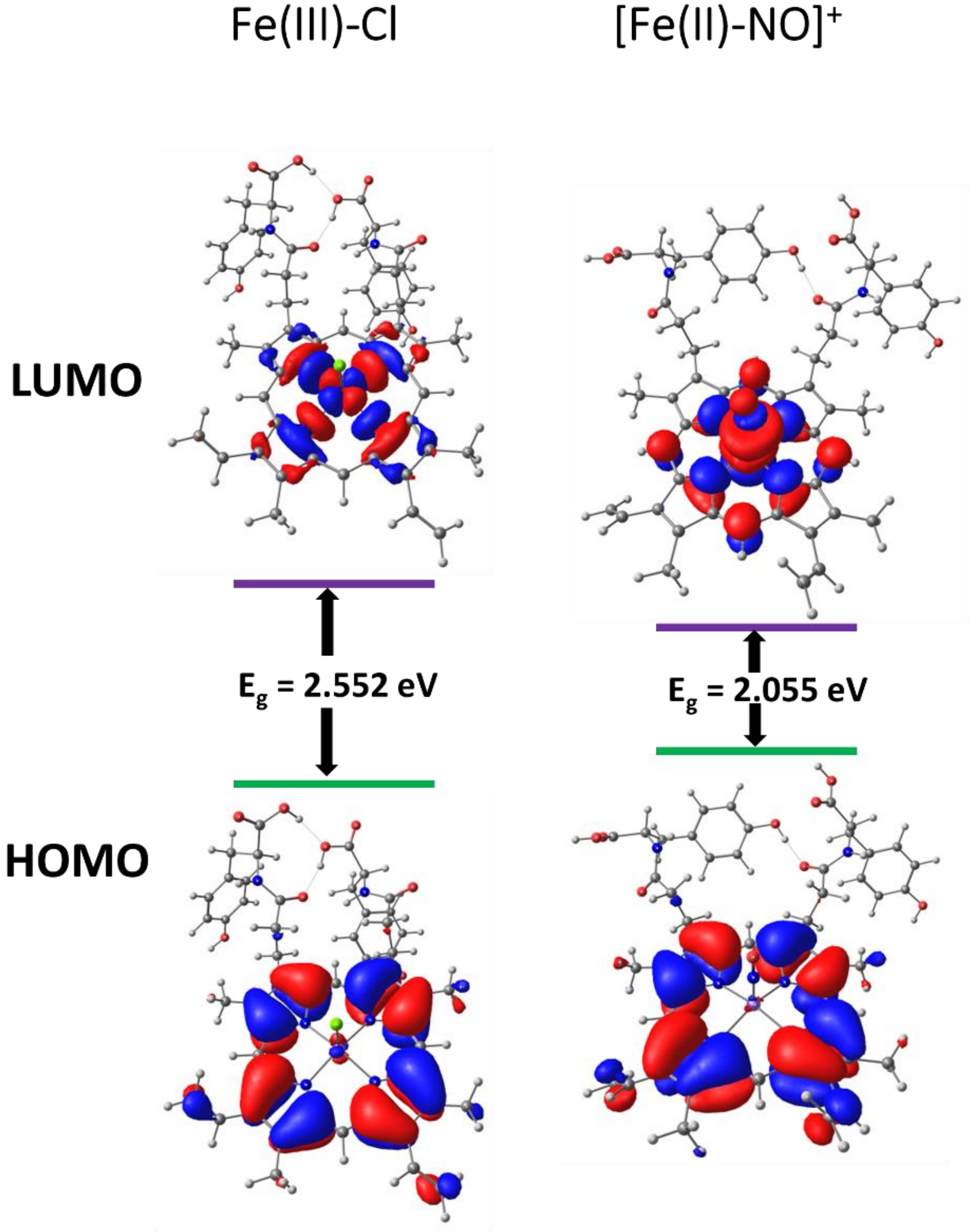

Figure S9. The calculated molecular orbital isosurfaces for different orbitals of the main structure of H-Tyros and following binding to •NO at the $[Fe(II)\text{-}NO]^+$ electronic state (isovalue 0.02), and the accompanied E_g using the B3LYP functional.

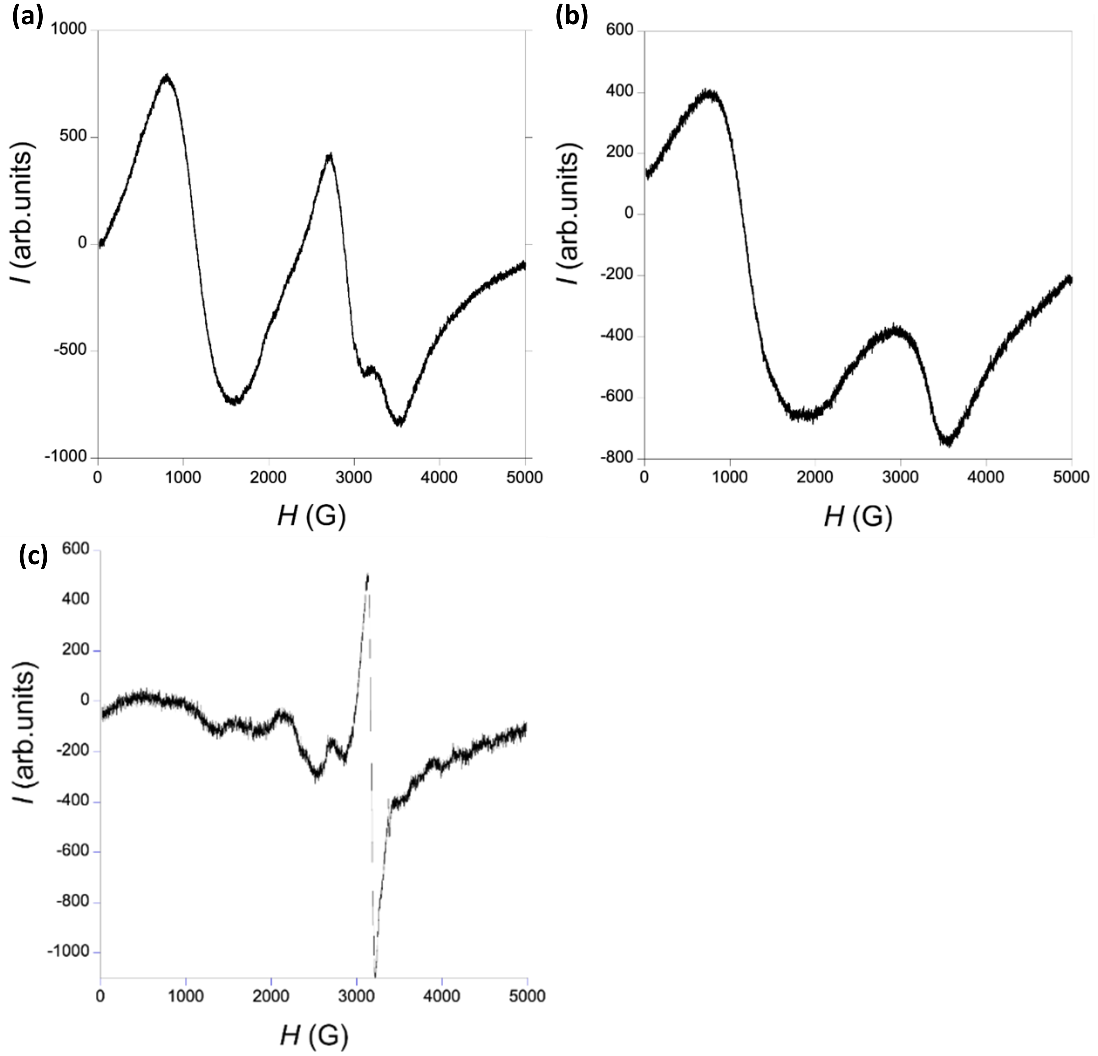

Figure S10. X-band ESR spectra of all hemin complexes as powder samples. Data collected at RT: a) Hemin; b) activated hemin; c) H-Dityros.

Table S15. ESR main parameters

**Compound**	**300 K**		**10 K**	
	**ESR parameters**	**assignment**	**ESR parameters**	**assignment**
**Hemin**	*g* = 5.71; *g* = 2.21	Fe(III) HS	*g* = 5.77; *g* = 2.04	Fe(III) HS
**Activated hemin**	*g* = 5.28; *g* = 1.94	Fe(III) HS	*g* = 5.90; *g* = 2.03	
**H-Tyra**	–		*g* = 5.93; *g* = 2.03	
**H-Tyros**	–		g = 5.92; *g* = 2.03	

**H-Dityros**	*g* = 2.17	Fe(II) LS + organic radical	*g* = 5.92; *g* = 2.03	

Table S16. The % decrease in the mathematical area under the •NO curve following the injection of each compound in the DETA-NO-containing culture medium. Results are presented as mean ± s.d, n = 3.

Compound	Concentration (µM)	% decrease in area under the curve
**Hemin**	4	75.78 ± 0.58
	8	83.75 ± 0.27
**H/Tyra**	4	67.09 ± 0.56
	8	80.28 ± 0.43
**H/Tyros**	4	86.85 ± 0.45
	8	67.10 ± 4.96
**H/Dityros**	4	66.85 ± 0.59
	8	61.10 ± 3.15

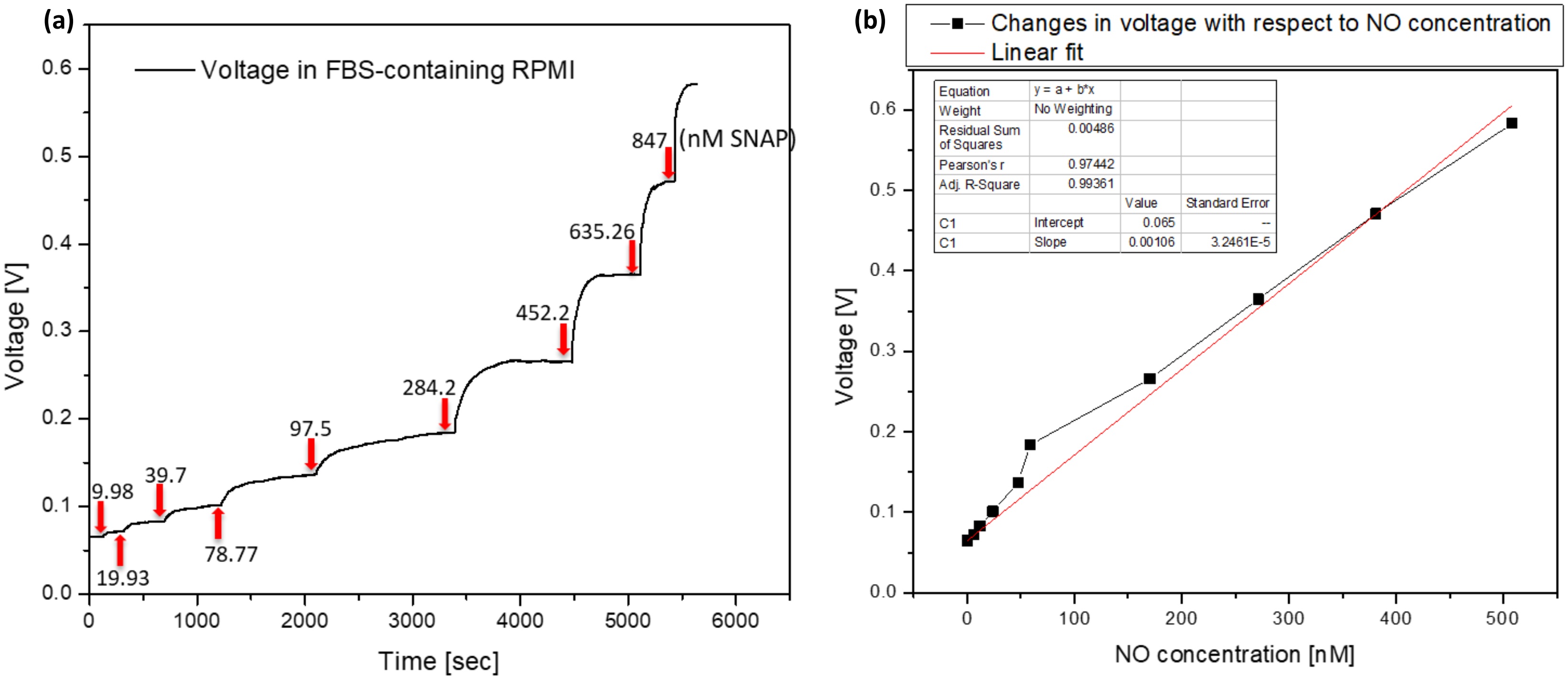

Figure S11. a) The change in voltage reading following the injection of different concentrations of SNAP in FBS-containing RPMI, and **b)** the accompanied standard curve.

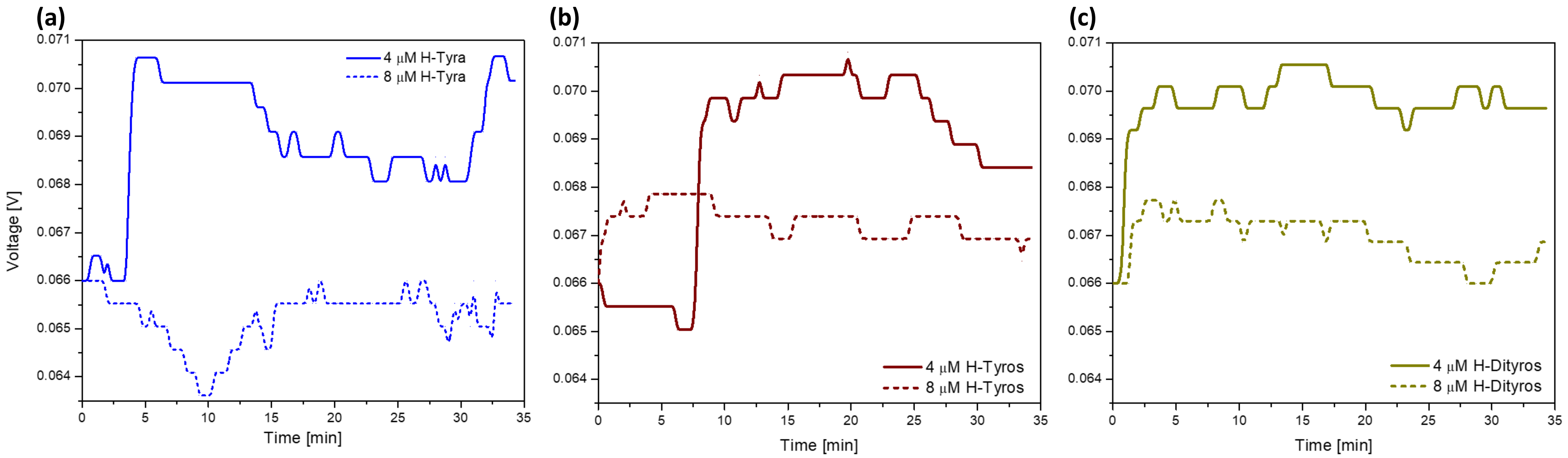

Figure S12. Temporal changes in the voltage signal following the injection of H-Tyra **(a)**, H-Tyros **(b)** and H-Dityros **(c)** at the concentrations 4 and 8 μM into FBS-containing medium. Results are presented as mean values, n = 3.

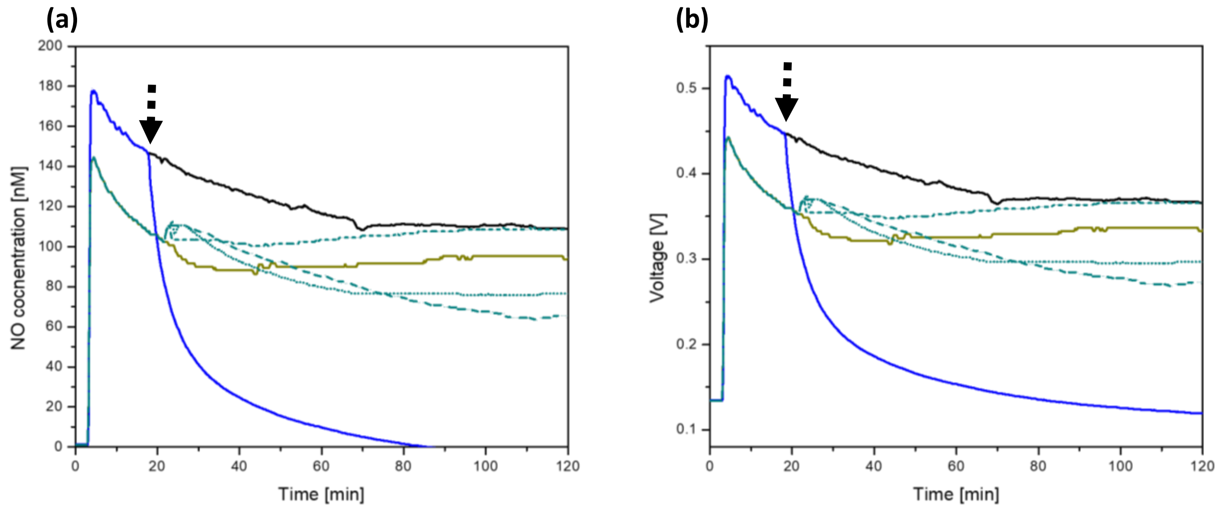

Figure S13. Temporal changes in the •NO levels following the injection of 300 μM DETA-NO only into FBS-containing medium (Black colour) and medium incubated for 16 hours at 37 °C (dark yellow colour), or after the subsequent addition of 8 μM **H-Tyra** diluted freshly to the testing medium (blue colour), or previously diluted in culture medium at the concentrations 1000 μM (dark cyan colour, dashed line), 100 μM (dark cyan colour, short dashed line), or 20 μM (dark cyan colour, short dotted line) **(a)**, and the accompanied voltage signal **(b).** Results are presented as mean values, n = 3.

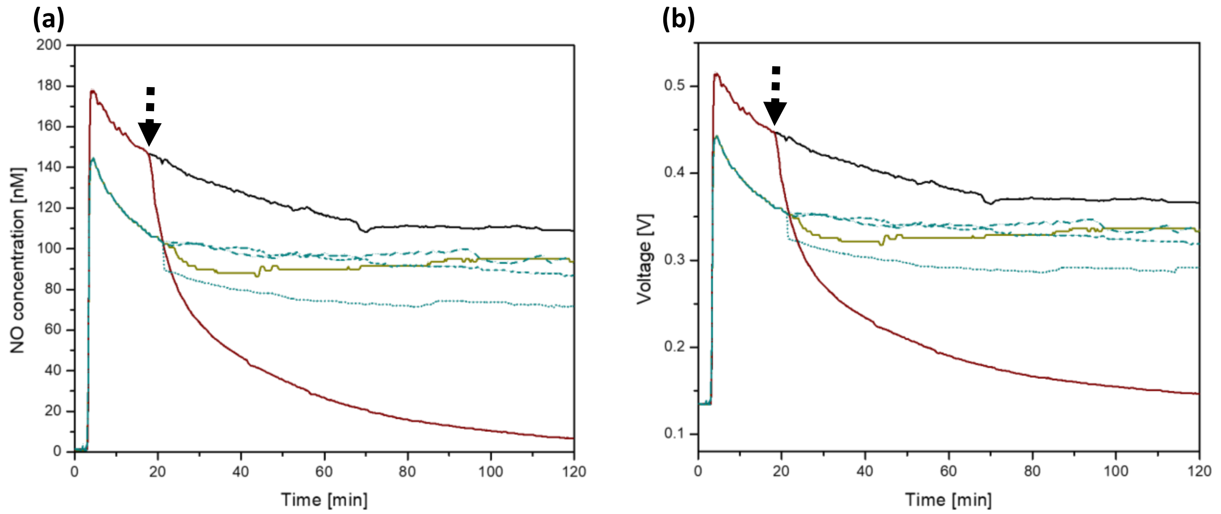

Figure S14. Temporal changes in the •NO levels following the injection of 300 μM DETA-NO only into FBS-containing medium (Black colour) and medium incubated for 16 hours at 37 °C (dark yellow colour), or after the subsequent addition of 8 μM **H-Tyros** diluted freshly to the

testing medium (wine colour), or previously diluted in culture medium at the concentrations 1000 μM (dark cyan colour, dashed line), 100 μM (dark cyan colour, short dashed line), or 20 μM (dark cyan colour, short dotted line) **(a)**, and the accompanied voltage signal **(b).** Results are presented as mean values, n = 3.

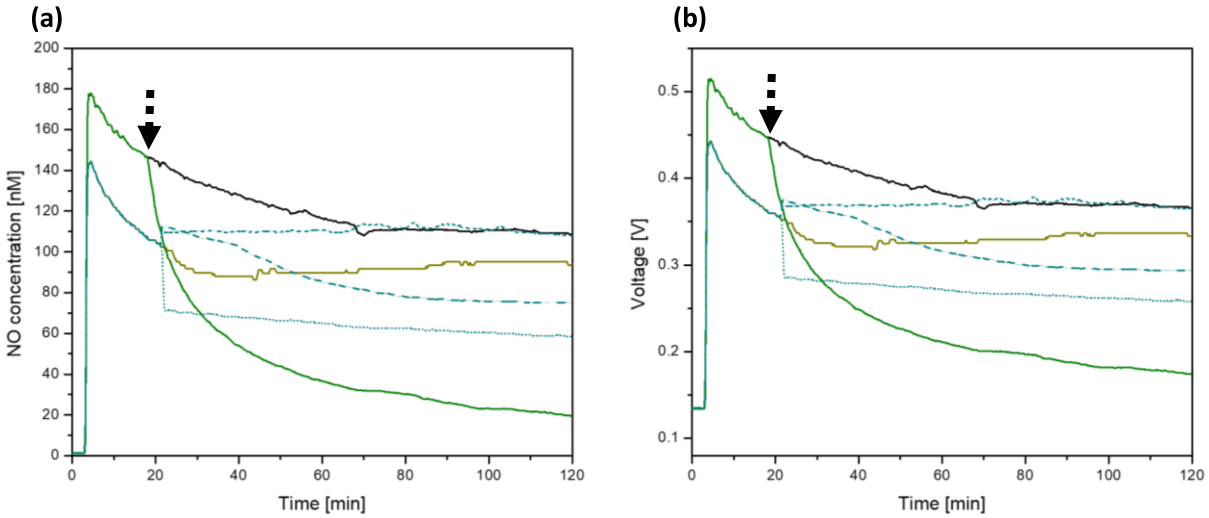

Figure S15. Temporal changes in the •NO levels following the injection of 300 μM DETA-NO only into FBS-containing medium (Black colour) and medium incubated for 16 hours at 37 °C (dark yellow colour), or after the subsequent addition of 8 μM **H-Dityros** diluted freshly to the testing medium (green colour), or previously diluted in culture medium at the concentrations 1000 μM (dark cyan colour, dashed line), 100 μM (dark cyan colour, short dashed line), or 20 μM (dark cyan colour, short dotted line) **(a)**, and the accompanied voltage signal **(b).** Results are presented as mean values, n = 3.

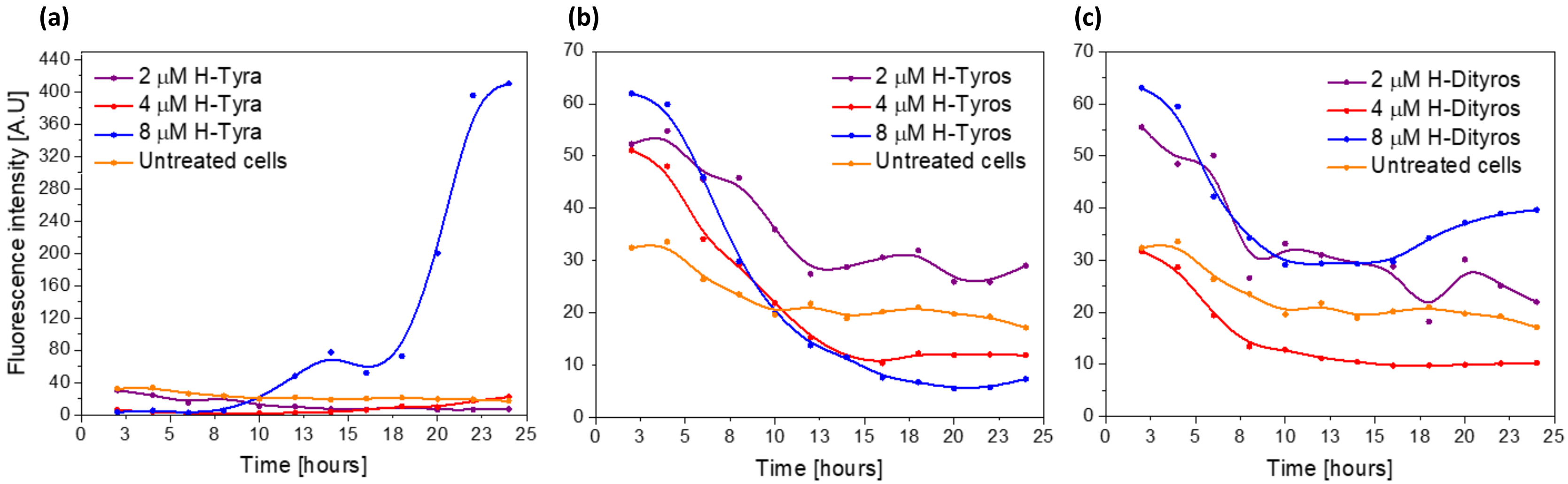

Figure S16. Kinetics of the changes in levels of intracellular •NO, detected by DAF-FM- fluorescence in MDA-MB-231 cells treated with either H-Tyra **(a)**, H-Tyros **(b)** or H-Styr **(c)** at the concentrations 2 (purple colour), 4 (red colour) or 8 μM (blue colour). The DAF-FM fluorescence in the untreated cells is expressed by the orange colour. Results are presented as mean values, n = 3.

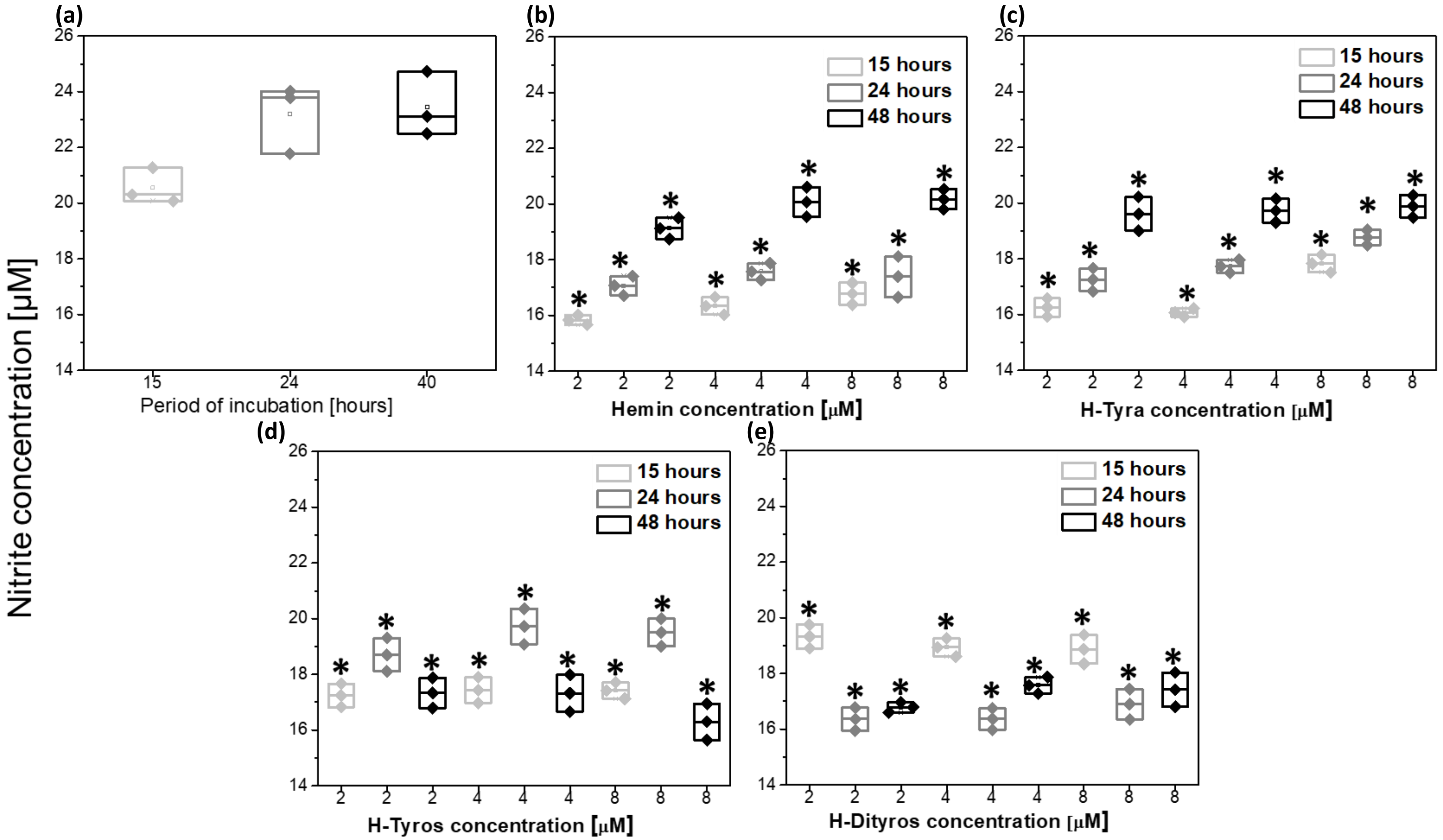

Figure S17. The changes in nitrite concentration in FBS-free medium following the treatment of MDA-MB-231 cells with either 300 μM DETA-NO only **(a)**, or in combination with hemin **(b),** H-Tyra **(c),** H-Tyros **(d),** or H-Dityros **(e)** at the concentrations 2, 4 or 8 μM and incubation for 15 (light gray colour), 24 (gray colour) and 40 hours (black colour). Results are presented as mean ± s.d, n = 3. *P < 0.05 versus the cells treated with DETA-NO only, using a two-tailed unpaired student t-test.

Table S17. Quantification of total iron extracted from MDA-MB-231 cells following treatment with H-Tyra, H-Tyros, or H-Dityros at the concentrations 4 and 8 μM in the presence/absence of DETA-NO. Results are presented as mean (in pmol/10000 cells) ± s.d, n = 3. $^{\#,*,\S}$, $P < 0.05$ versus the untreated cells, DETA-NO only-treated cells and for cells treated with combination of a compound and DETA-NO versus those without DETA-NO treatment, respectively using a two-tailed unpaired student t-test.

		**4 μM**		**8 μM**	
		**Without DETA-NO**	**With DETA-NO**	**Without DETA-NO**	**With DETA-NO**
**Untreated cell**	1.33 ± 0.05				
**DETA-NO only**	1.07 ± 0.06				
**H-Tyra**		6.92 ± 0.55$^{\#}$	9.31 ± 0.87*,§	4.23 ± 0.35$^{\#}$	4.82 ± 0.55*,§
**H-Tyros**		5.11 ± 0.46$^{\#}$	6.58 ± 0.51*,§	5.25 ± 0.51$^{\#}$	10.89 ± 0.95*,§
**H-Dityros**		5.66 ± 0.64$^{\#}$	6.33 ± 0.35*,§	5.91 ± 0.45$^{\#}$	8.64 ± 0.44*,§

Table S18. Quantification of total iron extracted from iNOS-transduced cells following treatment with hemin, H-Tyra, H-Tyros, or H-Styr at the concentrations 4 and 8 μM Results are presented as mean (in pmol/10000 cells) ± s.d, n = 3. $^{\#}$, $P < 0.05$ versus the untreated cells using a two-tailed unpaired student t-test.

		**4 μM**	**8 μM**
**Untreated cell**	1.91 ± 0.59		
**Hemin**		5.74 ± 2.24$^{\#}$	3.33 ± 0.78$^{\#}$
**H-Tyra**		9.27 ± 1.98$^{\#}$	8.57 ± 1.08$^{\#}$
**H-Tyros**		5.33 ± 0.48$^{\#}$	5.38 ± 0.85$^{\#}$
**H-Dityros**		5.7 ± 0.58$^{\#}$	5.7 ± 0.22$^{\#}$

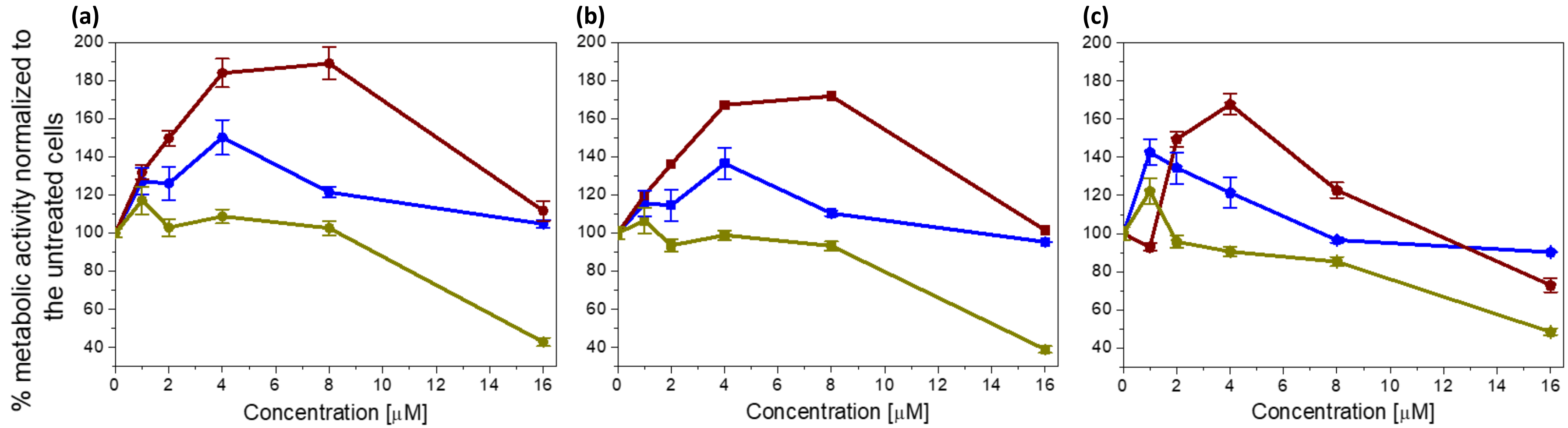

Figure S18. Metabolic activity of MDA-MB-231 cells following culture with different concentrations of H-Tyra (blue colour), H-Tyros (wine colour), or H-Dityros (dark yellow colour) for 24 **(a)**, 48 **(b)**, or 72 hours **(c)**. Results are presented as mean ± s.d, n = 3.

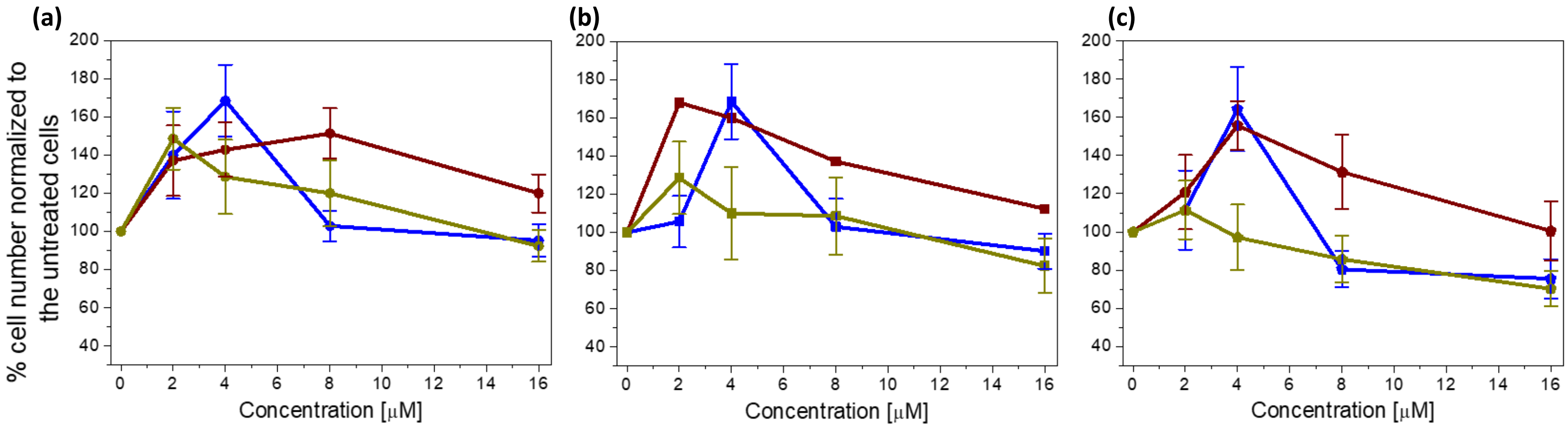

Figure S19. Counting of MDA-MB-231 cells following culture with different concentrations of H-Tyra (blue colour), H-Tyros (wine colour), or H-Dityros (dark yellow colour) for 12 **(a)**, 24 **(b)**, or 48 hours **(c)**. Results are presented as mean ± s.d, n = 3.

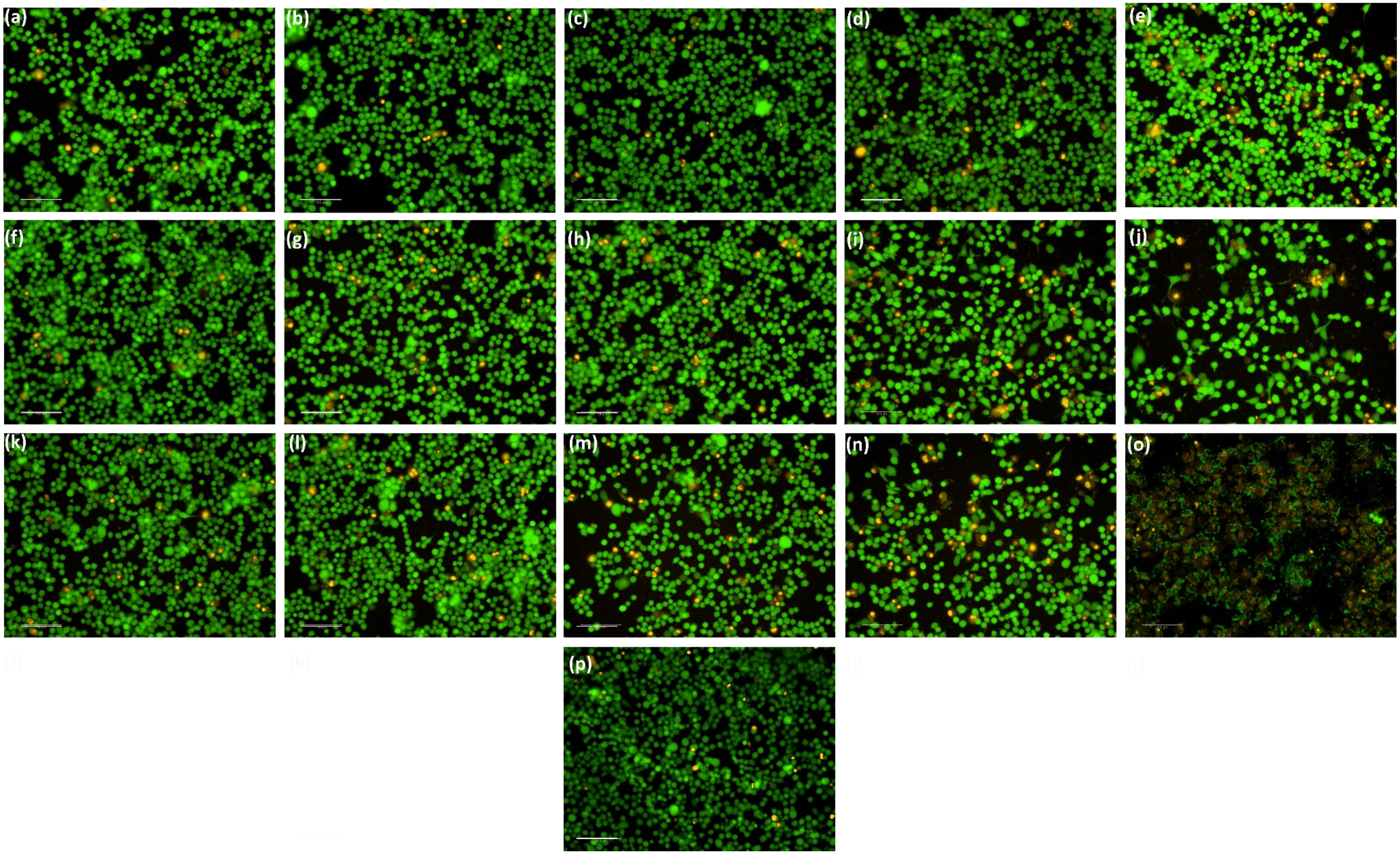
(a)
(b)
(c)
(d)
(e)
(f)
(g)
(h)
(i)
(j)
(k)
(l)
(m)
(n)
(o)
(p)

Figure S20. Calcein AM and EthD-1 live/dead staining of MDA-MB-231 following treatment with H-Tyra **(a-e)**, H-Tyros **(f-j)**, and H-Dityros **(k-o)** for 24 hours at the concentrations 1 **(a,f,k)**, 2 **(b,g,l)**, 4 **(c,h,m)**, 8 **(d,i,n)** and 16 μM **(e,j,o)**, or without any additives in control cells **(p)** for 24 hrs. Scale bar: 100 μm.

Video S1 The over time changes in levels of intracellular $^{\bullet}$NO, in DAF-FM-treated MDA-MB-231 cells, without $^{\bullet}$NO-donor. Scale bar: 400 µm.

Insert video 1.mp4 here

Video S2 The over time changes in levels of intracellular $^{\bullet}$NO, in DAF-FM-labelled MDA-MB-231 cells, after treatment with 300 µM DETA-NO only. Scale bar: 400 µm.

Insert video 2.mp4 here

Video S3 The over time changes in levels of intracellular $^{\bullet}$NO, in DAF-FM-labelled MDA-MB-231 cells, after treatment with 2 µM H-Tyros and 300 µM DETA-NO. Scale bar: 400 µm.

Insert video 3.mp4 here

Video S4 The over time changes in levels of intracellular $^{\bullet}$NO, in DAF-FM-labelled MDA-MB-231 cells, after treatment with 4 µM H-Tyros and 300 µM DETA-NO. Scale bar: 400 µm.

Insert video 4.mp4 here

Video S5 The over time changes in levels of intracellular $^{\bullet}$NO, in DAF-FM-labelled MDA-MB-231 cells, after treatment with 8 µM H-Tyros and 300 µM DETA-NO. Scale bar: 400 µm.

Insert video 5.mp4 here

Video S6 The over time changes in levels of intracellular $^{\bullet}$NO, in DAF-FM-labelled MDA-MB-231 cells, after treatment with 2 µM H-Tyra and 300 µM DETA-NO. Scale bar: 400 µm.

Insert video 6.mp4 here

Video S7 The over time changes in levels of intracellular $^{\bullet}$NO, in DAF-FM-labelled MDA-MB-231 cells, after treatment with 4 μM H-Tyra and 300 μM DETA-NO. Scale bar: 400 μm.

Insert video 7.mp4 here

Video S8 The over time changes in levels of intracellular $^{\bullet}$NO, in DAF-FM-labelled MDA-MB-231 cells, after treatment with 8 μM H-Tyra and 300 μM DETA-NO. Scale bar: 400 μm.

Insert video 8.mp4 here

Video S9 The over time changes in levels of intracellular $^{\bullet}$NO, in DAF-FM-labelled MDA-MB-231 cells, after treatment with 2 μM H-Dityros and 300 μM DETA-NO. Scale bar: 400 μm.

Insert video 9.mp4 here

Video S10 The over time changes in levels of intracellular $^{\bullet}$NO, in DAF-FM-labelled MDA-MB-231 cells, after treatment with 4 μM H-Dityros and 300 μM DETA-NO. Scale bar: 400 μm.

Insert video 10.mp4 here

Video S11 The over time changes in levels of intracellular $^{\bullet}$NO, in DAF-FM-labelled MDA-MB-231 cells, after treatment with 8 μM H-Dityros and 300 μM DETA-NO. Scale bar: 400 μm.

Insert video 11.mp4 here

Video S12 The over time changes in levels of intracellular $^{\bullet}$NO, in DAF-FM-treated iNOS-HCC1806 cells only. Scale bar: 400 μm.

Insert video 12.mp4 here

Video S13 The over time changes in levels of intracellular •NO, in DAF-FM-treated Vc-HCC1806 cells only. Scale bar: 400 μm.

Insert video 13.mp4 here

Video S14 The over time changes in levels of intracellular •NO, in DAF-FM-labelled iNOS-HCC1806 cells, after treatment with 2 μM H-Tyra. Scale bar: 400 μm.

Insert video 14.mp4 here

Video S15 The over time changes in levels of intracellular •NO, in DAF-FM-labelled iNOS-HCC1806 cells, after treatment with 4 μM H-Tyra. Scale bar: 400 μm.

Insert video 15.mp4 here

Video S16 The over time changes in levels of intracellular •NO, in DAF-FM-labelled iNOS-HCC1806 cells, after treatment with 8 μM H-Tyra. Scale bar: 400 μm.

Insert video 16.mp4 here

Video S17 The over time changes in levels of intracellular •NO, in DAF-FM-labelled iNOS-HCC1806 cells, after treatment with 2 μM H-Tyros. Scale bar: 400 μm.

Insert video 17.mp4 here

Video S18 The over time changes in levels of intracellular •NO, in DAF-FM-labelled iNOS-HCC1806 cells, after treatment with 4 μM H-Tyros. Scale bar: 400 μm.

Insert video 18.mp4 here

Video S19 The over time changes in levels of intracellular $^{\bullet}$NO, in DAF-FM-labelled iNOS-HCC1806 cells, after treatment with 8 μM H-Tyros. Scale bar: 400 μm.

Insert video 19.mp4 here

Video S20 The over time changes in levels of intracellular $^{\bullet}$NO, in DAF-FM-labelled iNOS-HCC1806 cells, after treatment with 2 μM H-Dityros. Scale bar: 400 μm.

Insert video 20.mp4 here

Video S21 The over time changes in levels of intracellular $^{\bullet}$NO, in DAF-FM-labelled iNOS-HCC1806 cells, after treatment with 4 μM H-Dityros. Scale bar: 400 μm.

Insert video 21.mp4 here

Video S22 The over time changes in levels of intracellular $^{\bullet}$NO, in DAF-FM-labelled iNOS-HCC1806 cells, after treatment with 8 μM H-Dityros. Scale bar: 400 μm.

Insert video 22.mp4 here

Video S23 The over time changes in levels of intracellular $^{\bullet}$NO, in DAF-FM-labelled iNOS-HCC1806 cells, after treatment with 5 mM aminoguanidine. Scale bar: 400 μm.

Insert video 23.mp4 here

References

[1] S. A. Okorochenkov, G. A. Zheltukhina, V. A. Roginsky, N. N. Nossik, S. L. Zheltukhin, V. E. Nebolsin, *J. Porphyr. Phthalocyanines* **2012**, *16*, 297–309.

[2] Frisch, M. J. *et al.* Gaussian 16, Revision C.01. (2016). Wallingford, CT.

[3] Koenig, D. F. The structure of α-chlorohemin. *Acta Crystallogr.* **18**, 663–673 (1965).

[4] Dennington, R., Keith, T. A., Millam, J. M. GaussView, Version 6.0.16. Semichem Inc., Shawnee Mission, KS (2016).

[5] Avogadro: an open-source molecular builder and visualization tool. Version 1.2.0. http://avogadro.cc/.

[6] O'boyle, N. M., Tenderholt, A. L. & Langner, K. M. cclib: A library for package-independent computational chemistry algorithms. *J. Comput. Chem.* **29**, 839–845 (2008).

[7] Zhurko, G. A. Chemcraft - graphical program for visualization of quantum chemistry computations. Ivanovo, Russia, 2005. https://chemcraftprog.com. (2005).

[8] Lee, C., Yang, W. & Parr, R. G. Development of the Colle-Salvetti correlation-energy formula into a functional of the electron density. *Phys. Rev. B* **37**, 785–789 (1988).

[9] Hay, P. J. & Wadt, W. R. Ab initio effective core potentials for molecular calculations. Potentials for K to Au including the outermost core orbitals. *J. Chem. Phys.* **82**, 299–310 (1985).

[10] Feller, D. The role of databases in support of computational chemistry calculations. *J. Comput. Chem.* **17**, 1571–1586 (1996).

[11] Schuchardt, K. L. *et al.* Basis set exchange: a community database for computational sciences. *J. Chem. Inf. Model.* **47**, 1045–1052 (2007).

[12] Pritchard, B. P., Altarawy, D., Didier, B., Gibsom, T. D. & Windus, T. L. A new basis set exchange: an open, up-to-date resource for the molecular sciences community. *J. Chem. Inf. Model.* **59**, 4814–4820 (2019).

[13] Hehre, W. J., Ditchfield, R. & Pople, J. A. Self-consistent molecular orbital methods. xii. further extensions of Gaussian-type basis sets for use in molecular orbital studies of organic molecules. *J. Chem. Phys.* **56**, 2257–2261 (1972).

[14] Hariharan, P. C. & Pople, J. A. The influence of polarization functions on molecular orbital hydrogenation energies. *Theor. Chim. Acta* **28**, 213–222 (1973).

[15] Rassolov, V. A., Pople, J. A., Ratner, M. A. & Windus, T. L. 6-31G* basis set for atoms K through Zn. *J. Chem. Phys.* **109**, 1223–1229 (1998).

[16] Dunning, T. H. Gaussian basis sets for use in correlated molecular calculations. I. The atoms boron through neon and hydrogen. *J. Chem. Phys.* **90**, 1007–1023 (1989).

[17] Balabanov, N. B. & Peterson, K. A. Systematically convergent basis sets for transition metals. I. All-electron correlation consistent basis sets for the 3d elements Sc-Zn. *J. Chem. Phys.* **123**, 64107 (2005).

[18] Glendening, E. D., Reed, A. E., Carpenter, J. E. & Weinhold, F. NBO Version 3.1.

[19] Alsharabasy, A. M., Glynn, S., Farràs, P. & Pandit, A. Protein nitration induced by Hemin/NO: A complementary mechanism through the catalytic functions of hemin and NO-scavenging. *Nitric Oxide* **124**, 49–67 (2022).

[20] Alsharabasy, A. M., Glynn, S., Farràs, P. & Pandit, A. Interactions between nitric oxide and hyaluronan implicate the migration of breast cancer cells. *Biomacromolecules **23**,* 3621–3647 (2022).

[21] Möller, B., Glaß, M., Misiak, D. & Posch, S. MiToBo - A Toolbox for image processing and analysis. *J. Open Res. Softw.* **4**, 17 (2016).
